# Supramolecular Guest Exchange in Cucurbit[7]uril for Bioorthogonal Fluorogenic Imaging across the Visible Spectrum

**DOI:** 10.1021/acscentsci.4c01080

**Published:** 2024-10-08

**Authors:** Ranjan Sasmal, Arka Som, Pratibha Kumari, Resmi V. Nair, Sushanta Show, Nisha Sanjay Barge, Meenakshi Pahwa, Nilanjana Das Saha, Sushma Rao, Sheeba Vasu, Rachit Agarwal, Sarit S. Agasti

**Affiliations:** † New Chemistry Unit, Chemistry & Physics of Materials Unit, and School of Advanced Materials (SAMat), Jawaharlal Nehru Centre for Advanced Scientific Research (JNCASR), Bangalore, Karnataka 560064, India; ‡ Department of Bioengineering, Indian Institute of Science, Bengaluru 560012, Karnataka India; § Evolutionary and Integrative Biology Unit and Neuroscience Unit, Jawaharlal Nehru Centre for Advanced Scientific Research (JNCASR), Bangalore, Karnataka 560064, India

## Abstract

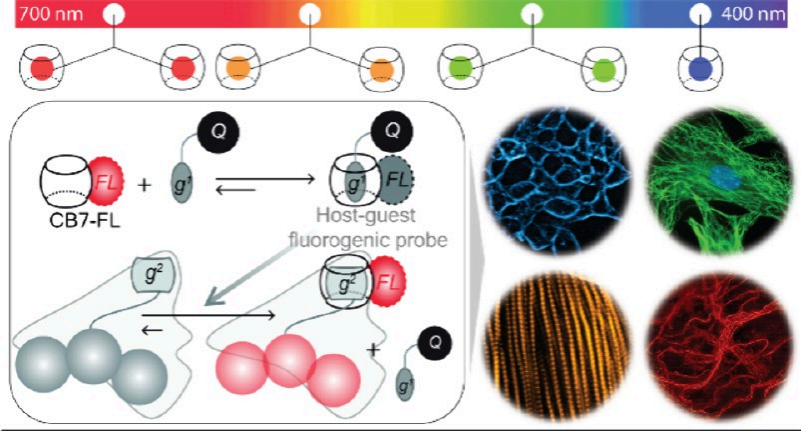

Fluorogenic probes that unmask fluorescence signals in response to bioorthogonal reactions are a powerful new addition to biological imaging. They can significantly reduce background fluorescence and minimize nonspecific signals, potentially enabling real-time, high-contrast imaging without the need to wash out excess fluorophores. While diverse classes of highly refined synthetic fluorophores are now readily available, integrating them into a bioorthogonal fluorogenic scheme still requires extensive design efforts and customized structural alterations to optimize quenching mechanisms for each specific fluorophore scaffold. Herein, we present a highly generalizable strategy that can produce an efficient bioorthogonal fluorogenic response from essentially any readily available fluorophore without further structural alterations. We designed this strategy based on the macrocyclic cucurbit[7]uril (CB7) host, where a fluorogenic response is achieved by programming a guest exchange reaction within the macrocyclic cavity. We employed this strategy to rapidly create fluorogenic probes across the visible spectrum from diverse fluorophore scaffolds, which enabled no-wash imaging in live cells and tissues with minimal background signal. Finally, we demonstrated that this strategy can be combined with metabolic labeling for fluorogenic detection of metabolically tagged mycobacteria under no-wash conditions and paired with covalently clickable probes for high-contrast super-resolution and multiplexed imaging in cells and tissues.

## Abstract 2

We introduce an easy-to-implement and highly generalizable supramolecular strategy to create bioorthogonal fluorogenic probes by programming guest exchange in the cucurbit[7]uril macrocyclic host.

## Introduction

Understanding living systems and unraveling their fundamental biological processes critically relies on the ability to observe specific biomolecules with a high spatiotemporal resolution in cells and tissues. Such efforts are greatly benefited by combining advanced fluorescence microscopy techniques with appropriate labeling strategies.^1−8^ In recent years, with the advent of bioorthogonal reactions that are robust and compatible with living systems, bioorthogonal strategies have emerged as attractive new labeling tools for advanced biological imaging.^9−14^ It offered an exquisite reactivity-based chemical labeling tool to biology, enabling specific tagging of a diverse set of biomolecules in their native environment with highly refined synthetic fluorophores. However, a major challenge that initially held back its use in a variety of advanced imaging applications is attributed to fluorescence background from unbound or nonspecifically bound synthetic fluorophores postlabeling. Although one could utilize rigorous washing steps to remove excess unbound probes, nonspecifically bound fluorescent probes are even harder to eliminate by washing. In addition, in the case of live cell or *in vivo* conditions, it is impossible to implement a washing step that can possibly clear excess or nonspecifically bound probes. Moreover, washing steps are not well tolerated for staining of scant cell populations (e.g., circulating tumor cells) in a point-of-care microfluidic device or in precise pulse-chase experiments where shorter time interval measurements are desired.^15^ These challenges can be elegantly tackled by utilizing a bioorthogonal fluorogenic labeling scheme, which provides an appealing solution for real-time background-free imaging without washing or clearance steps.^16−19^ The fluorescent probes used for such an approach exhibit an increase in fluorescence upon reacting to their specific biorthogonal counterpart, alleviating the problem of a background signal from unbound or nonspecifically bound probes. Although vast libraries of highly refined synthetic fluorophore families are readily available now, incorporating them into a fluorogenic labeling scheme remains a significant challenge, requiring additional customized structural design and synthesis efforts. Additionally, expanding the spectrum of fluorogenic bioorthogonal transformations by incorporating motifs with unique orthogonal reactivity profiles is highly desirable to enhance the scope of fluorogenic imaging for simultaneous probing of multiple cellular targets (i.e., multiplexed imaging).

**Scheme 1:**
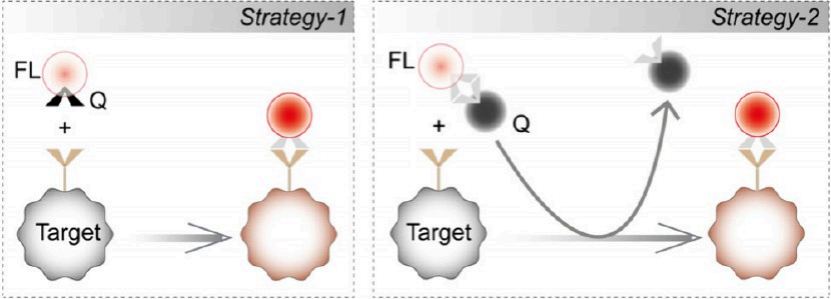
General Design Strategies for Bioorthogonal Fluorogenic Imaging

The most common design principle for a bioorthogonal fluorogenic probe is to strategically quench the intrinsic fluorescence of a dye until a specific bioorthogonal reaction eliminates this quenching effect, restoring the latent fluorescence.^20^ In a few instances, dye scaffolds have also been generated using bioorthogonal reactions.^21−26^ Conceptually quenching of the dyes can be achieved in two ways (Scheme 1): fluorophores can be armed with a suitable quencher moiety that can be either chemically converted to a spectroscopically nonperturbing functionality or eliminated during the labeling process.^20^ In the first concept, bioorthogonal reactive groups (azide/alkyne or tetrazine) are strategically incorporated into the dye skeleton such that they can act as a quencher moiety for the dye.^27−32^ In this case, the quenching effect is eliminated upon bioorthogonal conversion of the quenching moiety. Arguably, the most important example of this concept is tetrazine (Tz) probes, which gained popularity due to their fast reactivity via inverse-electron-demand Diels–Alder cycloaddition reaction.^33−43^ Tz has been shown to quench fluorescence via various photophysical mechanisms, including Forster resonance energy transfer (FRET),^44,45^ through-bond energy transfer (TBET),^31,32^ Dexter-type electron exchange,^46^ and photoinduced electron transfer (PET).^47^ Over the past few years, a range of design strategies has been investigated in collaboration with different fluorophore scaffolds to harness various Tz-mediated quenching mechanisms and achieve fluorogenic tetrazine probes extending up to the far-red/NIR range.^31,32,35,38−47^ While such fluorogenic probes have been promising, the quenching strategies are not generalizable to diverse libraries of fluorophore families, requiring extensive design efforts to customize quenching mechanisms for each fluorophore scaffold. In addition, access to these fluorogenic probes often requires direct alteration of the core fluorophore skeleton, demanding reoptimization of the laborious fluorophore synthesis scheme and tackling critical synthetic challenges. As an alternative way of designing fluorogenic probes for labeling, one might consider adapting the second concept where bioorthogonal reaction can lead to the release of the quenching group.^48,49^ This can take advantage of the readily/commercially available and highly optimized dye-quencher pairs to generate a fluorogenic response. This strategy is highly generalizable, and it can essentially incorporate any fluorophores into a fluorogenic labeling scheme by simply pairing them with a suitable quencher molecule. However, designing an appropriate bioorthogonal reaction scheme that can harness the benefit of this concept to achieve fluorogenic labeling of target molecules in cells remains challenging. It has been adapted only in rare instances for fluorogenic labeling of biomolecules in cells; however, even in such cases, it employs a sluggish Staudinger ligation reaction of phosphene probe.^48^ Herein, we demonstrate a fast and readily generalizable supramolecular guest exchange strategy that can easily integrate any highly optimized dye-quencher pairs for rapid fluorogenic labeling of biological targets. In addition, capitalizing on the unique/orthogonal reactivity profile of this supramolecular motif, we bolster efforts to track multiple biomolecule targets simultaneously via multiplexed bioorthogonal fluorogenic labeling.

**Scheme 2:**
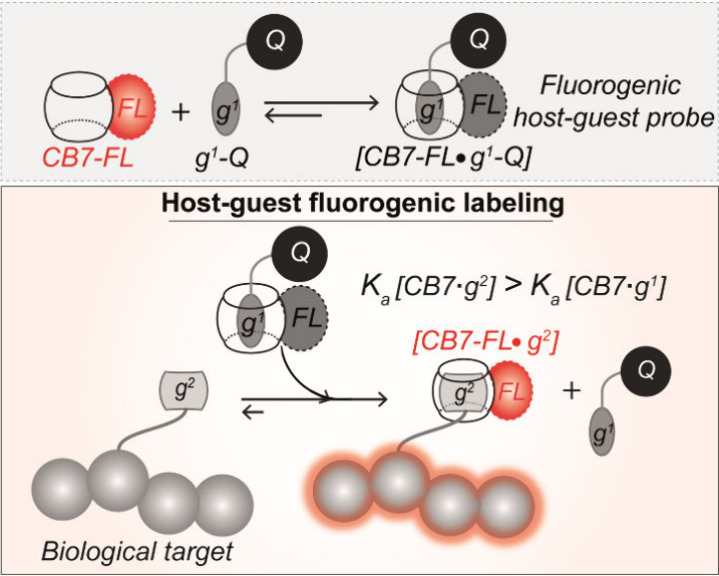
Schematic Showing the Working Principle of Bioorthogonal Supramolecular Fluorogenic Imaging CB7-conjugated fluorophores (CB7-FLs) are complexed with complementary guest-conjugated dark quenchers (g^1^-Qs) via host–guest recognition to generate fluorogenic probes. Guest exchange reaction between the quenched CB7-FL·g^1^-Q host–guest complex and a high-affinity guest g^2^ eliminates the quenching effect, restoring the latent fluorescence. This forms a fluorescently active reporter complex with the target biomolecule.

Besides covalent chemistry, an alternative approach toward target labeling is to leverage synthetic host–guest assembly by employing a pair of complementary molecular recognition partners. Most recently, noncovalent host–guest binding pairs based on macrocyclic CB7 host have emerged as a promising bioorthogonal imaging tool for visualizing biomolecules both in cells and *in vivo*, often termed as “non-covalent click chemistry”.^50−57^ This is largely fueled by the exceptional molecular recognition property of CB7 with an ability to form ultrastable (K_a_ up to 10^17^ M^–1^) and highly chemo-selective 1:1 host–guest complexes in biological complexities.^58−70^ In addition, their high association kinetics, small size, chemical tractability, and robust chemical structures further boost their potential for advanced imaging applications. Importantly, with the recent demonstrations of the enzymatic,^50^ metabolic,^71^ and genetic^72^ incorporation ability of host–guest elements into biomolecular structures, it is now positioned to offer a powerful alternative to covalent reactions with many complementary benefits. Herein, building on the foundation of the CB7-based host–guest chemistry, we now demonstrate an easily generalizable strategy for designing noncovalent fluorogenic probes across the visible spectrum for bioorthogonal imaging. We designed this strategy by programming guest exchange reactions in the synthetic supramolecular host–guest system based on CB7. Scheme 2 illustrates the intrinsic design principle for generating the supramolecular fluorogenic response based on fluorophore-conjugated CB7 reporter probes (CB7-FLs). To suppress the fluorescence of the reporter probes, CB7-FLs are complexed with complementary guest-conjugated dark quenchers (g^1^-Qs) via host–guest recognition. Our fluorogenic labeling scheme strategically leverages a dynamic guest exchange reaction involving the quenched CB7-FL·g^1^-Q host–guest complex and a high-affinity guest g^2^ covalently linked to the specific biological target of interest (depicted in Scheme 2). The incubation of the quenched CB7-FL·g^1^-Q complex with the g^2^-tagged biomolecules induces a guest exchange reaction, resulting in the formation of a new energetically favorable CB7-FL·g^2^ host–guest complex. The guest exchange event activates the fluorescence of CB7-FL, selectively generating fluorescently active reporter complexes (CB7-FL·g^2^) that are exclusively bound to the biomolecule of interest. This facilitates the visualization of target molecules while keeping the excess probes in a quenched state, thereby enabling background-free imaging under a no-wash condition. To date, CB7-FLs were employed either directly for imaging experiments,^50,73,74^ or Forster Resonance Energy Transfer (FRET) pair was developed for monitoring biological processes;^75,76^ however, to the best of our knowledge, fluorogenic labeling where fluorescence activation occurs upon specific target engagement has not been demonstrated in biological complexities. Furthermore, our investigation revealed the excellent performance of this supramolecular fluorogenic probe in complex biological samples, effectively visualizing target molecules within the intracellular environment of live cells and tissue samples. It also proved to be effective across various labeling platforms, including antibody-based methods, small molecule-based techniques, and even strategies that exploit the cell’s own synthesis machinery (metabolic labeling). Overall, our current strategy significantly expands the scope of noncovalent chemistry in bioorthogonal imaging, generating a fluorogenic effect that can substantially improve the signal-to-background ratio in various fluorescence microscopy applications and facilitating rapid, no-wash imaging of a myriad of target analytes from complex biological specimens. The use of synthetic host–guest supramolecular labels for fluorogenic imaging offers several advantages over covalent click labels and well-known protein-based binding pairs, such as streptavidin–biotin.^77−80^ For instance, host–guest complexation kinetics are typically diffusion-controlled and occur at a much faster rate (*k*_on_ ∼ 10^9^–10^10^ M^–1^ s^–^^1^) compared to their covalent counterparts, which are kinetically slower (typically *k*_on_∼ 1–10^4^ M^–^^1^ s^–^^1^). This rapid labeling is particularly beneficial for precise pulse-chase assays, where a high labeling rate is crucial. Unlike covalent reactive groups, which often compromise stability for reactivity, synthetic host–guest labels (e.g., CB7 and 1-adamantylamine) utilize a stable chemical structure that is robust in physiological environments. Additionally, these small synthetic host–guest pairs avoid common issues associated with biotin–streptavidin pairs, such as the large size and potential immunogenicity of proteins, as well as interference from endogenous biotin. The smaller size of synthetic host–guest recognition pairs, compared to protein-based binding pairs, also facilitates efficient cellular uptake. Given these advantages, we believe that these host–guest fluorogenic probes will be a valuable tool for biological imaging and will create new opportunities for microscopic and nanoscopic investigations *in vitro*, *in vivo*, and in diagnostic settings.

## Results and Discussion

**Figure 1:**
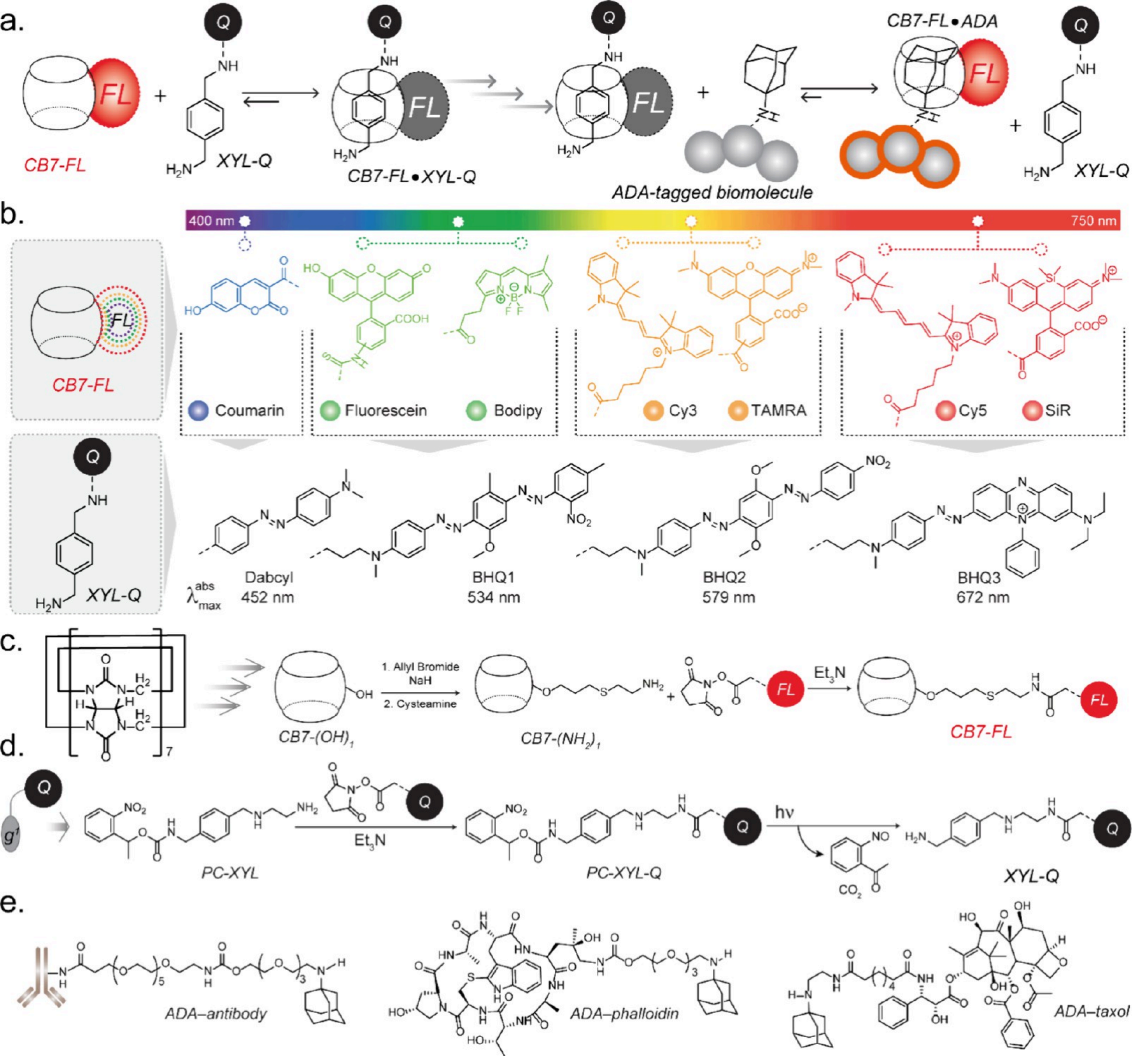
Molecular design for host–guest-based fluorogenic imaging. (a) Selected guests (XYL and ADA) and schematic depicting guest exchange reactions for fluorogenic labeling of biological targets. (b) Molecular structures of CB7-conjugated fluorophores (CB7-FLs) and XYL-conjugated quenchers (XYL-Qs). XYL-Qs complex with spectrally matching CB-FLs to generate fluorogenic probes. (c) Synthetic scheme for producing CB7-FLs. (d) Functionalization scheme of XYL with quenchers. (e) ADA-conjugated targeting agents were employed in the fluorogenic imaging study for specific targeting of biomolecules in cells and tissues.

The success of this supramolecular fluorogenic imaging strategy was critically dependent on the integration of a suitable host–guest pair that permits selective monovalent complexation (1:1) under physiologically relevant conditions. For this purpose, we relied on CB7 as it stands apart from other synthetic host molecules because of its most striking ability to display exceptionally strong monovalent host–guest molecular recognition (K_a_ up to 10^17^ M^–1^) toward specific and bioorthogonal guest molecules in biological medium.^58−70,81,82^ In addition, depending on the structure of the guest molecules, the affinities can also be tailored from 10^6^ M^–1^ to 10^18^ M^–1^, thereby not only permitting selective complex formation at a biologically viable concentration (typically below μM) but also allowing us to program guest exchange reactions based on the affinity gradient of various CB7-guest complexes. Specifically, for the design of our exchange-based fluorogenic probes, we choose two guests- g^1^: *p*-xylylenediamine (XYL, K_a_ ∼ 10^8^ M^–1^) and g^2^: 1-adamantylamine (ADA, K_a_ ∼ 10^14^ M^–1^) (Figure 1a).^61^ XYL (g^1^) was tethered to the quencher, while ADA (g^2^) served as the label for the target biomolecule. Given their considerable binding energy difference, we hypothesized that the change in free energy resulting from the XYL to ADA exchange in the CB7 cavity would effectively promote the forward progression of the guest exchange reaction. This, in turn, would lead to the selective accumulation of the CB7-FL•ADA complex on the target biomolecule. Furthermore, the choice of XYL as g^1^ was guided by its high affinity, exceeding that of CB7′s promiscuous binding to native biological motifs (e.g., 10^5^–10^6^ M^–1^ for certain amino acids on proteins).^83,84^ This strategic choice was incorporated to ensure minimal activation of the CB7-FL•XYL-Q complex by native biological motifs, thereby preventing nonspecific signal generation from untargeted (non-ADA triggered) activation of CB7-FLs.

To generate fluorogenic probes across the visible spectrum, CB7 is directly attached with a library of well-established fluorophores (CB7-FLs), spanning emissions from 450 to 675 nm (Figure 1b). Matching with the fluorophore’s emission, XYL moiety is armed with appropriate dark quenchers (XYL-Qs) to generate quenched host–guest probes (Figure 1b). In addition to Forster Resonance Energy Transfer (FRET) based quenching, these highly optimized quenchers can take advantage of the static quenching, thereby providing excellent fluorescence quenching without the residual background signal. To synthesize CB7-FLs, we first used a photochemical derivatization method, described by Bardelang and Ouari, to synthesize monohydroxylated CB7 (CB7(OH)_1_).^85−87^ CB7(OH)_1_ was then converted in two steps to amine derivative (CB7-(NH_2_)_1_), which was subsequently attached to the fluorophores via amide linkage (Figure 1c).^88^ On the other hand, XYL-Qs were synthesized from a nitrobenzyl-protected XYL derivative (Figure 1d). To decorate the protein/biomolecule of interest with high-affinity ADA guest, we prepared two types of ADA conjugated targeting agents (Figure 1e): 1) ADA conjugated antibody and 2) ADA conjugated small molecule binders, phalloidin (for actin targeting) and taxol (for microtubule targeting).

**Figure 2:**
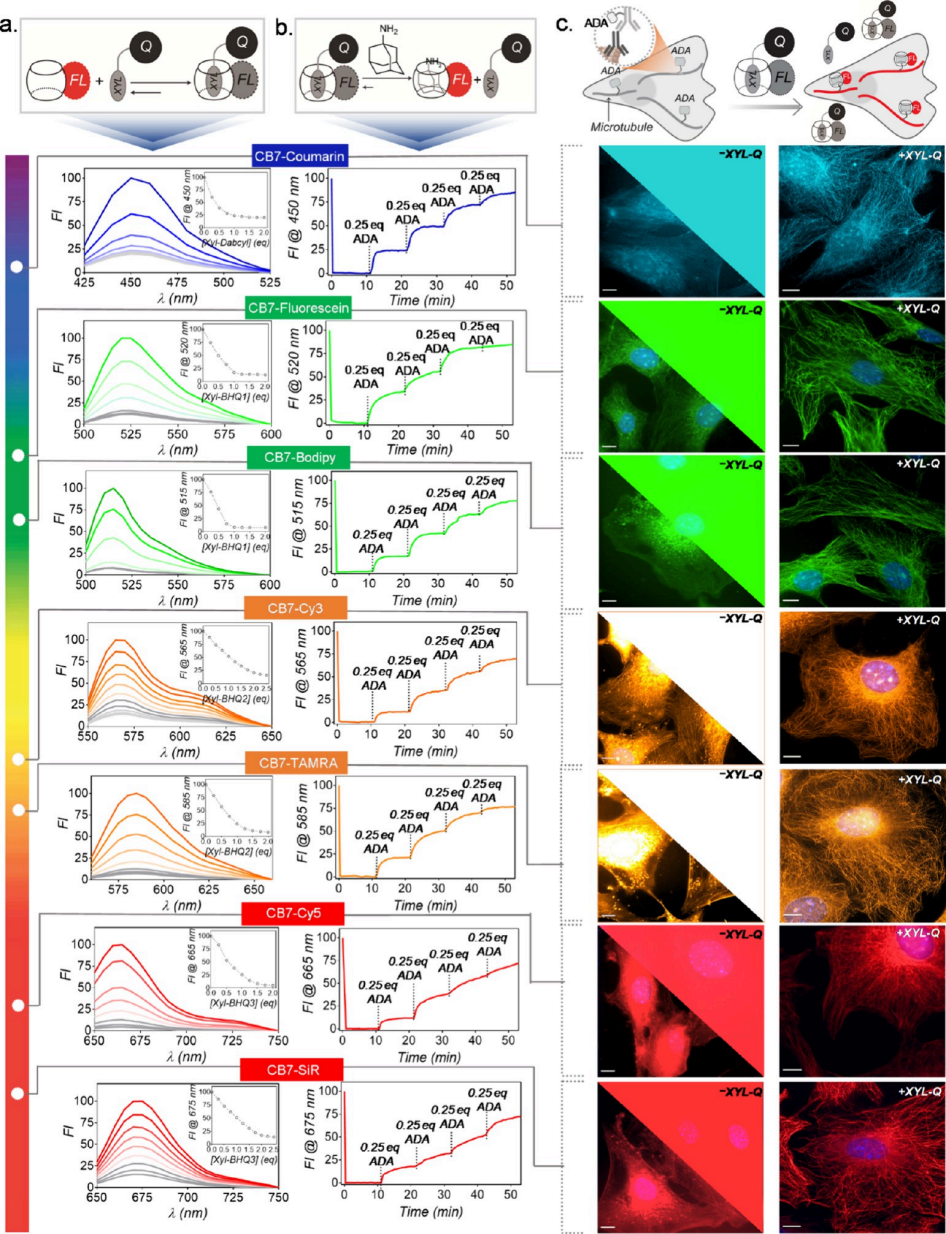
Characterization of guest exchange reaction and demonstration of fluorogenic imaging in fixed cells. (a) Fluorescence titration of CB7-FL with XYL-Q showing fluorescence quenching upon host–guest complex formation. All the CB7-FLs exhibited excellent quenching upon the addition of respective XYL-Q. (b) Fluorescence recovery upon addition of ADA to the quenched probes. Stepwise addition of the substoichiometric equivalent of ADA showed an efficient fluorescence recovery from the quenched probes. (c) Fluorogenic imaging of microtubules from fixed cells using ADA-Abs and CB7-FL·XYL-Q quenched probes. Right panel: High-contrast microtubule images were observed from fluorogenic probes without removing excess unbound probes. Left panel (top right corner and bottom left corner): Control studies with only CB7-FLs (without XYL-Qs) did not lead to an appreciable microtubule visualization due to high background fluorescence from the unbound fluorophores. The top right corner shows images that are represented with the same brightness contrast as that of the fluorogenic images. The bottom left corner presents images with adjusted brightness contrast, best suited to visualize signals from cells. Scale bar: 10 μm (c).

We first studied the quenching of CB7-FLs by titrating 1 μM solution of CB7-FLs with XYL-Qs. Seven different CB7-FLs were assayed against their spectrally matching XYL-Q molecules (Figure 2a). Notably, all the CB7-FLs exhibited excellent quenching upon the addition of equimolar or slightly higher stoichiometry of respective quencher-conjugated XYL guests. The slight variation can be attributed to the structural diversity of the fluorophores, which can have a moderate influence on the CB7•XYL binding affinity. To establish the role of host–guest recognition in the observed fluorescence quenching, we titrated CB7-FLs with a control quencher derivative (EtA-Q) that does not contain any recognition motif for CB7. As shown in Supporting Figure S1, we did not observe any significant quenching upon equivalent addition of EtA-Qs, indicating that the recognition-mediated CB7•XYL complexation is the critical mechanism that brings the fluorophore near to the quencher moiety for effective suppression of fluorescence. Additionally, the formation of a 1:1 complex between CB7-FL and XYL-Q was confirmed via Matrix-assisted laser desorption/ionization mass spectrometry (MALDI-MS) analysis (Supporting Figure S2), where monovalent complexes are detected in the mass signature. Next, we evaluated the fluorescence activation characteristics of the quenched CB7-FL•XYL-Q complex by introducing the ADA guest with a relatively higher binding affinity (Figure 2b). The addition of ADA to the solution containing the quenched complex resulted in an immediate increase in fluorescence intensity (Figure 2b and Supporting Figure S3). All the quenched complexes underwent significant fluorescent enhancement with rapid response kinetics (Supporting Figure S3). This consequence can be directly attributed to the elimination of the quenching effect through guest exchange in the CB7 cavity, leading to the formation of the fluorescently active CB7-FL•ADA complex and the release of XYL-Q from the CB7 cavity. Mechanistically, the guest exchange occurs via dynamic supramolecular interactions, wherein the ADA guest, with a higher binding affinity, seizes an empty CB7 during the dynamic exchange of the CB7-FL•XYL-Q complex.^89−91^ Marked by significantly greater affinity and a longer half-life of complexation, the addition of ADA guest ultimately results in the preferential accumulation of CB-FL in the fluorescently active CB7-FL•ADA complex state. MALDI-MS analysis also supported the formation of the CB7-FL•ADA complexes upon addition of ADA to the quenched complex (Supporting Figure S3). We also observed a stepwise fluorescence recovery from the quenched complex upon addition of substoichiometric equivalent of ADA (Figure 2b), indicating a free energy-driven displacement reaction rather than being a concentration-driven one. Overall, these spectroscopic studies clearly demonstrate the fluorogenic nature of the host–guest complex, which is efficiently triggered by the guest exchange reaction with a high-affinity bioorthogonal guest molecule.

We next evaluated the ability of this noncovalent guest exchange strategy for fluorogenic imaging of a specific biological target in cellular settings. Before target imaging, we first probed the stability of the quenched CB7-FL•XYL-Q complex in the cellular environment. For this purpose, quenched CB7-TAMRA•XYL-BHQ2 complex was incubated with the mouse embryonic fibroblast (MEF) cells and time-lapse fluorescence microscopy images were recorded from the surrounding media to observe any cell mediated dissociation of the quenched complex. Quantification of these time-lapse microscopy images showed a negligible increase in fluorescence intensity over time (Supporting Figure S4), indicating minimal nonspecific activation/dissociation of CB7-FL•XYL-Q quenched complex by native cellular components. Additionally, no appreciable fluorescence signal or nonspecific staining was observed from the cells upon incubation with the CB7-FL•XYL-Q quenched complex (Supporting Figure S5). We next investigated whether the higher affinity ADA guest covalently linked to a biological target molecule could be utilized for target-specific fluorogenic activation and visualization of the cellular entities under no-wash imaging conditions. To evaluate our hypothesis, we conjugated ADA with antibodies (ADA-Ab), utilizing the traditional amine NHS ester reaction, where ADA was attached to the lysine residues of the antibodies (Supporting for detailed protocol). Through MALDI-MS analysis, we determined that on average, ∼ 2.6 molecules of ADA were attached per antibody (Supporting Figure S6). The ADA conjugated antibody was then employed to target microtubules in MEF cells. After immunolabeling using ADA-Ab, MEF cells were incubated with the quenched CB7-FL•XYL-Q probes and subsequently imaged without performing additional washing steps required to remove unbound probes. Traditional labeling experiments were additionally conducted, wherein immunolabeled cells were stained with only CB7-FLs under identical conditions. This approach was aimed to assess the impact of unbound fluorescent probes on target visualization. In all cases, cells treated with the fluorogenic probes, consisting of complexed CB7-FL•XYL-Q probe, showed high-contrast staining of microtubules, whereas cells treated with only CB7-FL under identical conditions only led to barely visible microtubule structures (Figure 2c). Notably, with the host–guest fluorogenic probes, microtubules were clearly visible even under the widefield epi-fluorescence mode of microscopy, where the presence of any background fluorescence is known to significantly impact the image quality (Figure 2c). This indicates suppressed fluorescence from the unbound host–guest probes played a critical role in high-contrast target visualization. Notably, specific, and background-free staining of microtubules were consistently observed from all the spectrally different host–guest quenched probes, demonstrating the advantage of this easily generalizable noncovalent guest exchange-based strategy for rapid generation of multicolor fluorogenic probes. A quantitative estimation from fluorescence intensity profiling also indicated dramatically enhanced signal-to-noise from the CB7-FL•XYL-Q probes compared to CB7-FL alone (Supporting Figure S7). The specificity of the host–guest labeling approach was also validated via colocalization experiment with a microtubule targeted direct-fluorophore antibody conjugate (Supporting Figure S8). In order to gain a quantitative view of the fluorogenic labeling kinetics, time-lapse imaging was performed on ADA-targeted cells using a quenched CB7-TAMRA•XYL-BHQ2 probe (Supporting Figure S9). Intensity profile over time showed completion of the fluorogenic labeling within minutes, highlighting the suitability of these probes for a fast pulse-chase labeling experiment or labeling under high flow *in vivo* conditions. Fluorescence intensity was also found to be stable over time after reaching saturation which could be potentially useful for fluorescence tracking experiments. The potential of employing this fluorogenic approach for imaging other targets of interest with low abundance has also been explored through the imaging of the nuclear pore complex (NPC). Fluorogenic imaging conducted using the CB7–SiR·XYL–BHQ3 complex clearly visualized the localization of NPCs within the cells, which appeared as distinct dotted puncta on the nuclear envelope (Supporting Figure S10). Overall, these results clearly establish the rapid fluorogenic imaging capability of the host–guest probe across the visible spectrum with the advantages of no-wash imaging and substantially improved signal-to-noise ratio for fluorescence microscopy. In further support of the guest exchange mechanism and to establish its generalizability with other guest-tagging situations, we investigated whether a XYL-tagged antibody (XYL-Ab) could display target imaging via fluorophore localization. Given the dynamic exchange within the CB7-FL•XYL-Q complex, it was hypothesized that, during this process, some fraction of CB7-FL molecules would also distribute and occupy XYL labels on the antibody, thereby facilitating target visualization. To validate this, we utilized XYL-Ab to target microtubules in cells and conducted imaging experiment after incubation with the CB7-FL•XYL-Q complex. As shown in the Supporting Figure S11, fluorescence microscopy images illustrated specific microtubule staining in XYL-labeled cells, confirming the guest exchange mechanism and indicating its generalizability across various guest systems. However, it is noteworthy to emphasize that tagging antibodies with high-affinity guests (i.e., ADA) will yield a much higher concentration of fluorophores bound to the target molecules, thereby enhancing the signal intensity, all while requiring a significantly lower number of guests to be anchored to the target molecules via antibody labeling.

**Figure 3:**
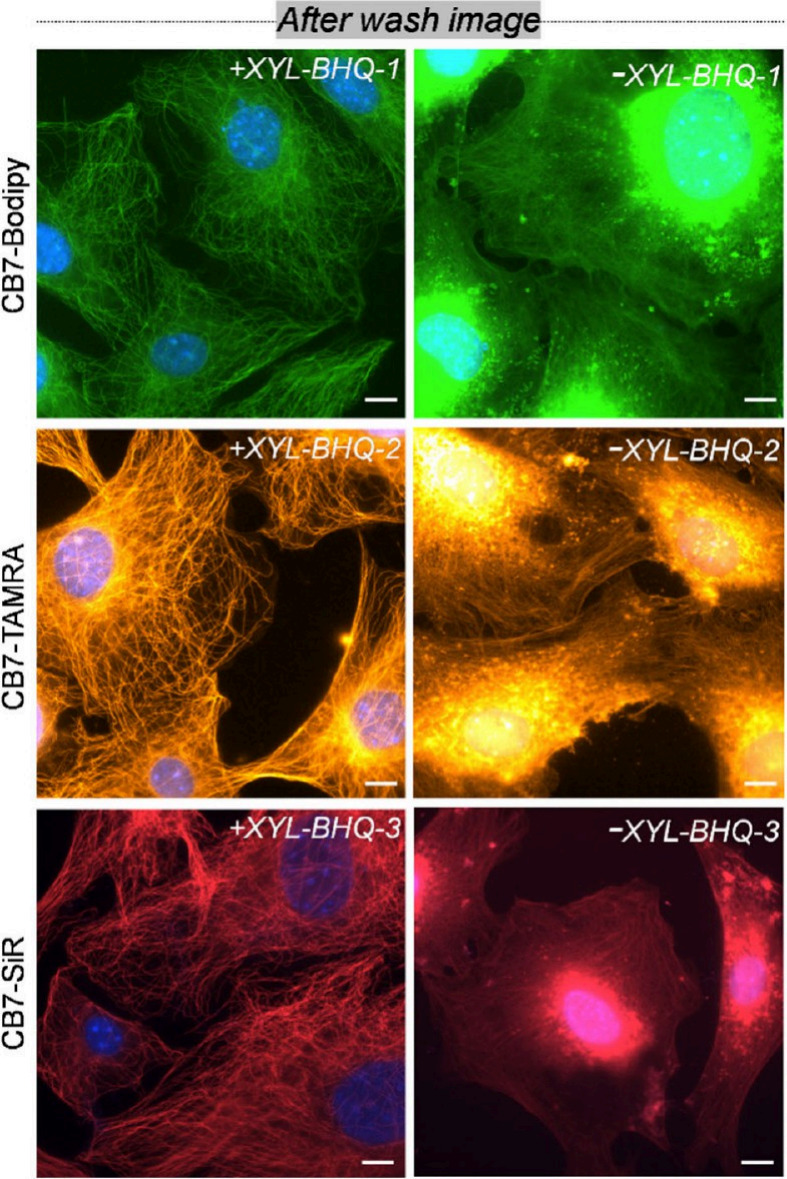
After-wash images from labeling with fluorogenic CB7 probes (+XYL-Q) and CB-FLs alone (−XYL-Q). A dramatically reduced off-target signal from the host–guest quenched probes (+XYL-Q) as compared to the CB7-FL (−XYL-Q) alone was observed due to the conditionally activable nature of the quenched probes. Scale bar: 10 μm.

In addition to suppressing background from unbound probes, we also explored whether our fluorogenic probe could reduce nonspecific signals by imaging microtubule-labeled cells after removing excess unbound probes. In this scenario, our fluorogenic probe was expected to offer two advantages: (1) The host–guest quenched probe only exhibits a fluorescence signal upon encountering the ADA counterpart, rendering nonspecifically bound probes ’invisible’ due to their nonfluorescent nature. (2) The affinity of the XYL guest quencher, exceeding CB7′s promiscuous binding to native biological motifs, would ensure minimal activation of the CB7-FL•XYL-Q complex by native biological motifs, thus preventing nonspecific signals during the untargeted (i.e., non-ADA bound) localization of free CB7-FLs. Accordingly, we tested the fluorogenic host–guest probe (+XYL-Q) with an included washing step and observed microtubule visualization with minimal off-target fluorescence signal. In contrast, CB7-FLs, when used alone (-XYL-Q), showed strong off-target signals even after repetitive washing (shown in Figure 3 and Supporting Figure S12). This contrasting effect further highlights the advantage of our fluorogenic probes in eliminating nonspecific signals over the direct use of CB7-FLs in bioorthogonal imaging. Furthermore, we also validated the effectiveness of the XYL guest in preventing CB7-FLs from adhering to the native biological system. To achieve this, we investigated whether washing cells with the free XYL guest (without the quencher) could rescue off-target bound CB7-FLs in the traditional imaging approach using CB7-FL alone and reduce the background. The results depicted in the Supporting Figure S13 clearly revealed enhanced microtubule visualization and reduced off-target signal after washing CB7-FL-stained cells with the XYL guest. This confirms the removal of off-target bound CB7-FL by XYL guest and supports our design choice of employing a guest with superior affinity, surpassing CB7′s broad binding to native biological motifs, for generating CB7 fluorogenic probes.

**Figure 4:**
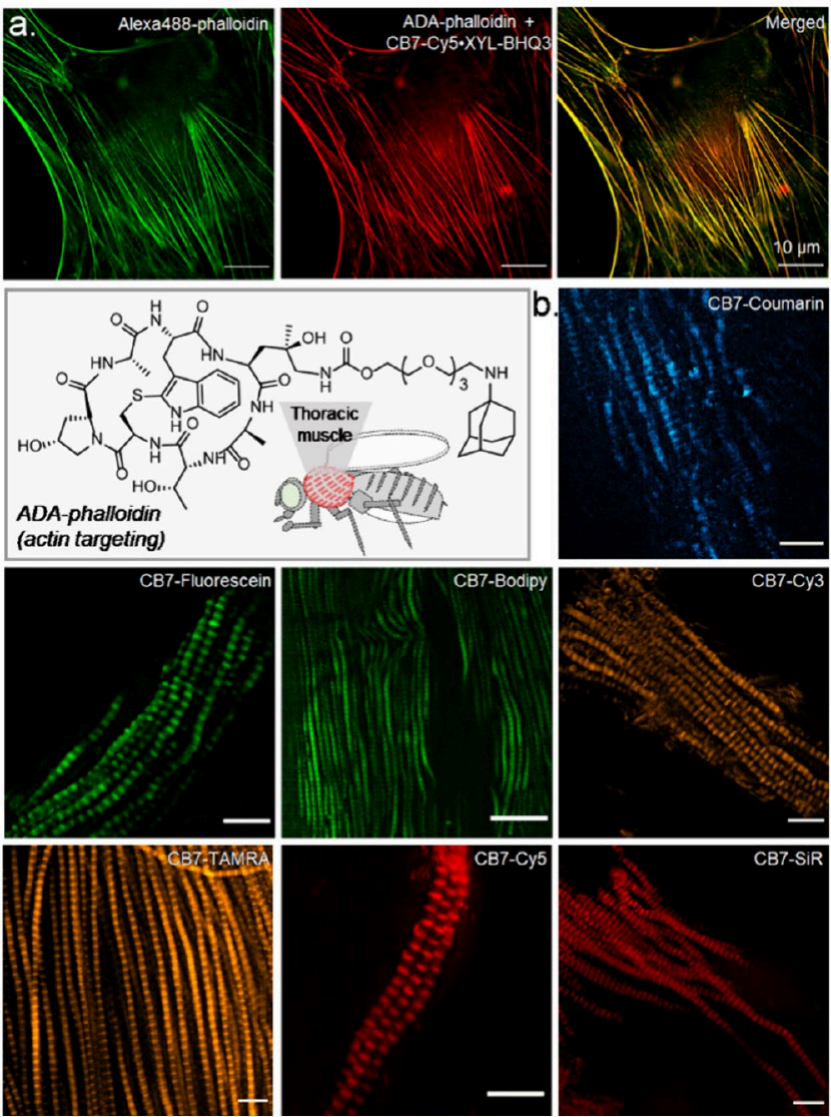
Host–guest fluorogenic imaging of the actin structures in fixed MEF cells and fixed thoracic muscle tissue of *Drosophila melanogaster*. (a) Two-color imaging of actin, comparing guest-modified targeting ligands to direct fluorophore-conjugated imaging agent. Additionally, the molecular structure of ADA-phalloidin that is used for targeting actin in muscle tissue is shown. (b) Epi-fluorescence images of the muscle tissue after incubation with spectrally diverse fluorogenic probes. We observed repeating band patterned actin distribution using a host–guest-based fluorogenic imaging strategy. Scale bar: 10 μm (a and b).

Compared to cells cultured on coverslips, labeling and imaging specific targets in tissue samples usually harbor a more significant challenge. Tissues possess a complex architecture, and with their inherently slow diffusion kinetics, it is even more challenging to rapidly remove unbound fluorophores or eliminate nonspecifically bound probes. We tested whether our noncovalent guest exchange strategy could be applicable for fluorogenic imaging in tissue samples to tackle these challenges. To test this, we attempted to label actin filaments in the thoracic muscle of *Drosophila melanogaster* using the fluorogenic probes. A small molecule binder of actin, namely, phalloidin, is attached with the ADA guest molecule and used for targeting the actin structure (Figure 4a). First, the target specificity of the phalloidin molecule after attachment with ADA guest was tested in cells, where actin filaments were simultaneously visualized with a traditional alexa488-phalloidin stain along with the ADA-phalloidin probe. Excellent colocalization of the two probes indicated the specificity of the ADA-phalloidin toward the intended actin target (Figure 4a). Next, we used ADA-phalloidin to target actin structures in the thoracic muscle of *Drosophila melanogaster* for fluorogenic imaging. Incubation of phalloidin-ADA conjugates with the tissue sample, and the subsequent addition of CB7-FL•XYL-Q fluorogenic probe resulted in specific and distinct visualization of a two-dimensional (2D) actin pattern in the muscle sample (Figure 4b). A repeating band patterned fluorescence, resembling intrinsic spatial organization actin in thoracic muscle, was prominent from the epi-fluorescence microscopy images. Notably, the visualization of actin patterns from the tissue section remained impressive when tested with the range of fluorogenic host–guest probes. We also performed three-dimensional (3D) imaging of the actin structure from the tissue samples to understand fluorogenic imaging capability inside a thick tissue sample. The 3D distribution of actin patterns in the ovary of *Drosophila melanogaster* was clearly visualized with minimal background over ∼80 μm axial direction (Supporting Figure S14). These results highlight the applicability of the host–guest fluorogenic probes in labeling and imaging complex tissue samples.

**Figure 5:**
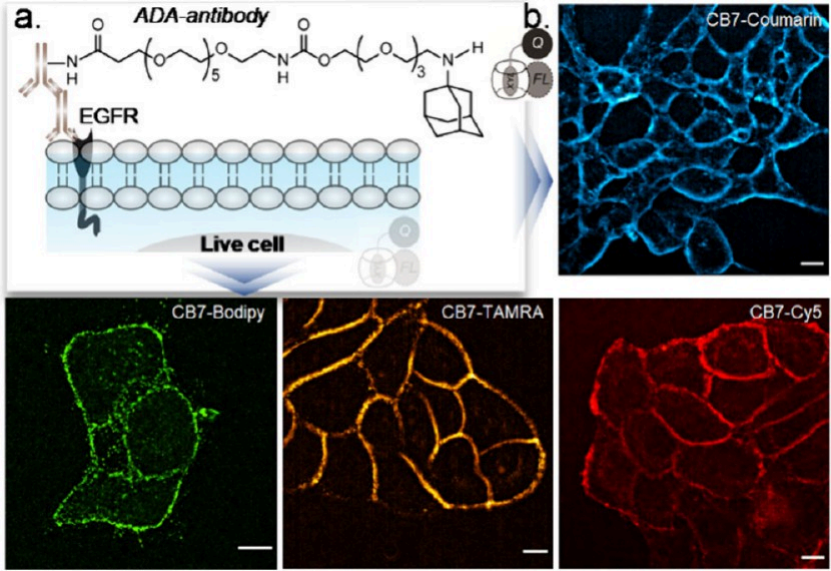
Live cell imaging of extracellular target using supramolecular fluorogenic probe. (a) Targeting strategy for EGFR on the live cell membrane. (b) Fluorescence images from the live cell after labeling EGFR with host–guest fluorogenic probes. The intense membrane staining in fluorescence images from EGFR overexpressing live A431 cells indicates successful fluorogenic labeling of EGFR on the live-cell surface. Scale bar: 10 μm (b).

**Figure 6:**
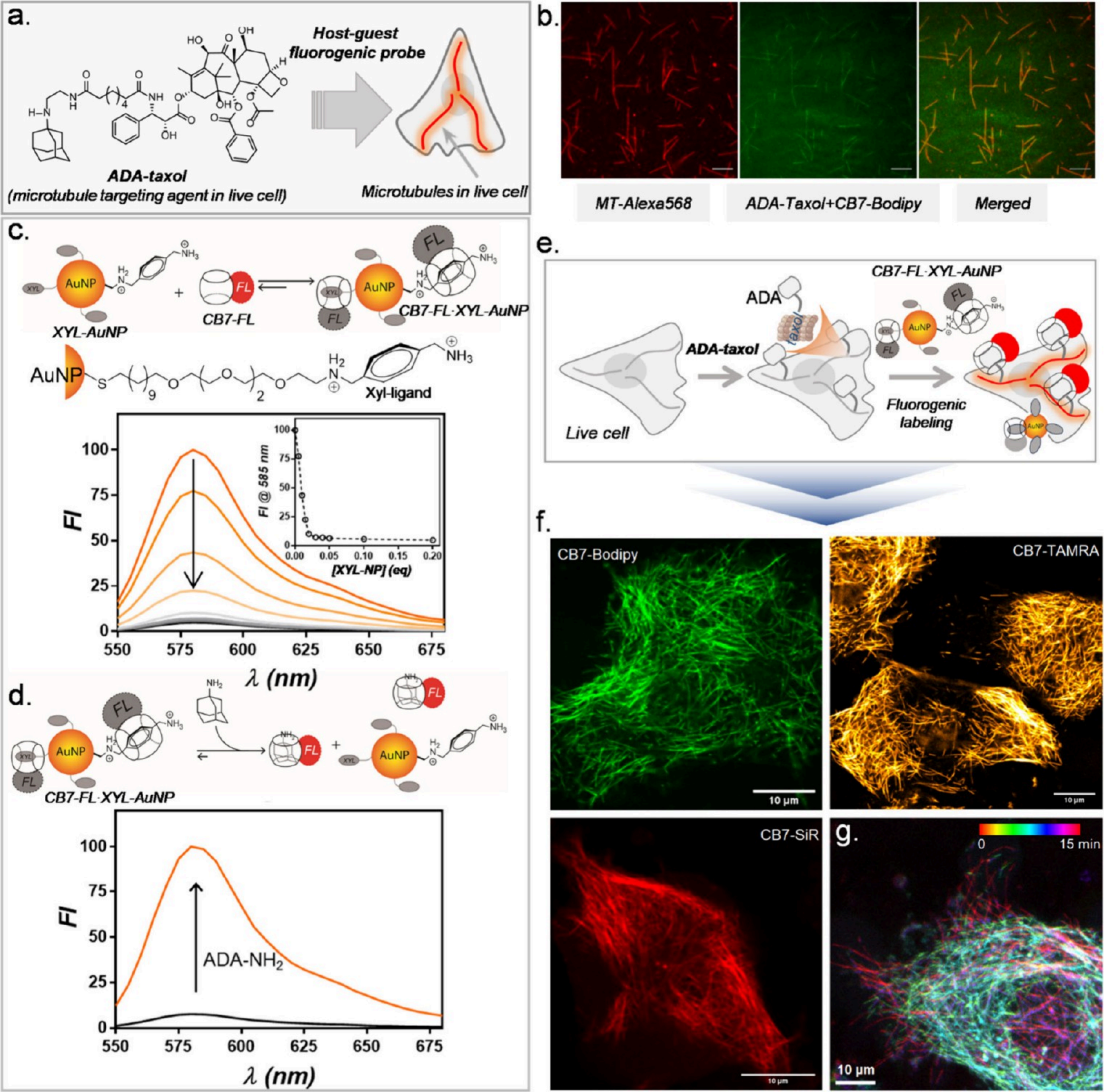
Live cell imaging of intracellular targets using supramolecular fluorogenic probes. (a) Schematic illustrating the utilization of ADA-conjugated taxol (ADA-taxol) for live cell imaging of microtubules. (b) Two-color imaging of *in vitro* polymerized microtubules, comparing imaging specificity with guest-modified targeting ligand (ADA-taxol) against direct fluorophore-conjugated microtubules. (c) Guest-modified AuNP-based quenching of CB-FLs. Fluorescence titration of CB7-TAMRA with XYL-AuNP demonstrating fluorescence quenching upon host–guest complex formation. (d) Fluorescence recovery upon addition of ADA to the AuNP-quenched CB7-FL probes. (e) Schematic illustrating the fluorogenic imaging strategy of microtubules in live HeLa cells using ADA-taxol and CB7-FL•XYL-AuNP quenched probes. (f) Live cell microscopy images demonstrating specific microtubule visualization using supramolecular fluorogenic probes. (g) Time-lapse microtubule imaging. Each time point image is color-coded and finally merged to demonstrate microtubule mobility. Scale bar: 10 μm (b and f), 5 μm (c).

We subsequently employed our noncovalent CB7-based fluorogenic probes for imaging of biological targets in living specimens. To achieve this goal, our initial aim was to image an extracellular target: specifically, the overexpression of epidermal growth factor receptor (EGFR) on the live A431 cell membrane (Figure 5a). After targeting EGFR with ADA-Ab, we incubated the A431 cells with the fluorogenic probes for imaging overexpressed EGFR receptors. Live A431 cells imaged without the washing, or excess probe removal step showed a strong fluorescence signal emanating from the membrane, indicating fluorogenic labeling of the EGFR on the live-cell surface (Figure 5b and Supporting Figure S15). Control experiments that are performed without adding ADA-Ab to the A431 cells or with an EGFR negative cell line (3T3 cells) showed negligible membrane fluorescence, indicating specificity of the host–guest probes for labeling targets in the living system (Supporting Figure S16). Next, we utilized our noncovalent fluorogenic labeling strategy for imaging of biological targets inside living cells. We used a small molecule-based microtubule binder, docetaxel, to specifically target polymeric microtubules in live cells. To afford fluorogenic microtubule imaging, we prepared an ADA derivative of docetaxel (ADA-taxol), where ADA is conjugated with docetaxel via a C6–linker (Figure 6a).

**Figure 7:**
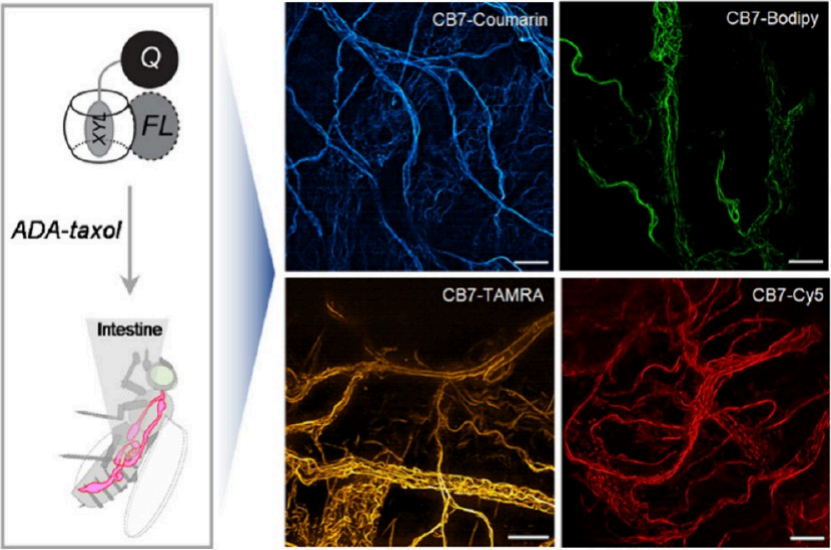
Host–guest fluorogenic imaging in live tissue. Fluorescence structured illumination microscopy (SIM) images captured from live intestine samples of *Drosophila melanogaster*. Live tissue samples were targeted with ADA-taxol and stained with fluorogenic host–guest probe, revealing the distribution of microtubules within the tissue specimen. Scale bar: 10 μm.

Initially, we assessed the specificity of docetaxel toward microtubules after derivatization with ADA through an *in vitro* experiment. In this experiment involving *in vitro* polymerized microtubules, the CB7-Bodipy•ADA-taxol complex was observed to colocalize with the directly Alexa568-labeled microtubules, confirming the target specificity of ADA-taxol (Figure 6b). However, a limiting factor that hindered the applicability of these probes for efficient intracellular labeling is the poor membrane permeability of the dyes/the dye-quencher pairs. Consequently, to advance this supramolecular fluorogenic strategy for live intracellular target imaging, we considered evaluating a new system where an efficient delivery vector could serve the dual purpose of intracellular dye transporter and act as a guest quencher. In pursuit of this objective, we explored the utilization of gold nanoparticles (AuNPs), recognized for their highly efficient cytosolic delivery capabilities and the ability to quench proximal dye fluorescence across a broad spectral range.^92−95^ In order to transform an AuNP (∼2 nm core diameter) into a guest quencher, we surface-functionalized the AuNPs with XYL guest conjugated thiolated ligand. This promoted the formation of quenched CB7-FL•XYL complex at the AuNP surface (Figure 6c). Fluorescence quenching of the CB7-FL dyes by XYL decorated AuNPs (XYL-AuNPs) was investigated by titrating 1 μM solution of a CB7-FL (here CB7-TAMRA) with an increasing concentration of XYL-AuNP. Notably, we observed an excellent quenching of CB7-TAMRA fluorescence upon addition of 0.02 equiv of XYL-AuNP, arising from the multivalent nature of the guest XYL-AuNP coupled with the efficient quenching property of the gold core (Figure 6c). Moreover, to elucidate the role of host–guest recognition in the observed fluorescence quenching, we titrated TAMRA-EtA, lacking any CB7 recognition motif, with XYL-AuNP. In this scenario, the addition of up to 0.2 equiv of XYL-AuNP did not lead to significant quenching (Supporting Figure S17a). This result suggests that recognition mediated CB7•XYL complexation plays a pivotal role in bringing the fluorophore into proximity with the AuNP surface, facilitating effective quenching of fluorescence. Additionally, we investigated the fluorescence activation characteristics of the quenched CB7-TAMRA•XYL-AuNP complex by introducing a relatively higher binding affinity ADA guest (Figure 6d). The addition of ADA to the quenched complex resulted in an immediate recovery of fluorescence intensity, directly attributed to the displacement of CB7-FL dye by ADA from the XYL-AuNP surface (Figure 6d). Overall, these spectroscopic studies demonstrate the fluorogenic nature of the AuNP host–guest complex, which is efficiently triggered by the guest exchange reaction with a high-affinity bioorthogonal guest molecule. Next, we used this quenched CB7-FL•XYL-AuNP complex to image the microtubule network inside live HeLa cells (Figure 6e). After targeting the microtubule with ADA-taxol, we incubated the HeLa cells with the CB7-FL•XYL-AuNP fluorogenic complexes for the target imaging. Live HeLa cells imaged without the inclusion of a washing step showed strong fluorescence signals originating from the microtubule filaments, indicating fluorogenic labeling of the microtubules inside the live cell (Figure 6f). High-contrast microtubule images with minimal background fluorescence indicated that XYL-AuNP not only efficiently transported CB7-FLs inside the cell but also kept the unbound FLs in a quenched state in the intracellular environment. In addition, the control experiment that was performed without adding ADA-taxol to the HeLa cells showed negligible microtubule fluorescence, indicating the specificity of the host–guest probes for intracellular labeling targets in the living system (Supporting Figure S17b). Microtubule mobility was also observed through time-lapse imaging. As illustrated in Figure 6g, when snapshots from different time points were merged into one image, with each time point shown in a different color, we could easily see the moving microtubules inside the live cell. Next, we evaluated whether the host–guest fluorogenic probes could be translated into even more complex settings, like live tissue imaging. In this regard, we chose to image the distribution of microtubules in intestinal tissue from *Drosophila melanogaster*. ADA-taxol was used to stain microtubules in live intestinal tissue. Subsequently, CB7-FL•XYL-Q fluorogenic probe in Schneider’s medium was incubated with the ADA-labeled tissues, and imaging was performed via super-resolution structured illumination microscopy (SIM). The SIM images that are acquired with the range of host–guest fluorogenic probes clearly showed the distribution of microtubules in the live intestine tissue (Figure 7). Notably, all the spectrally different fluorogenic probes performed efficiently to label and visualize the distribution of microtubules from the live intestine tissues. Overall, these results prove the applicability of our noncovalent CB7-based fluorogenic probes for imaging complex living specimens.

**Figure 8:**
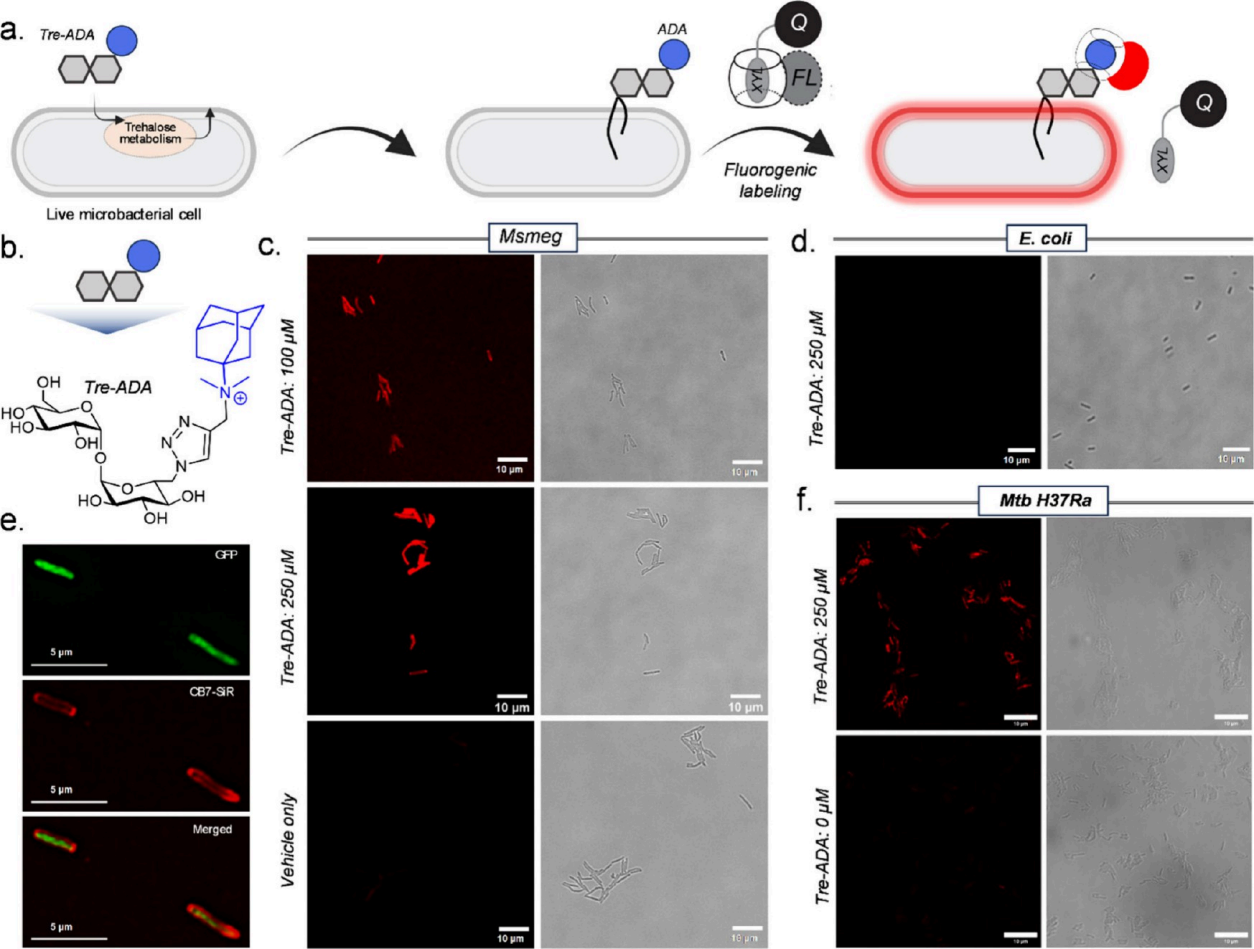
Fluorogenic imaging of metabolically labeled mycobacteria. (a) Illustration of the guest-modified trehalose-based metabolic labeling and fluorogenic strategy. (b) Chemical structure of the guest-modified trehalose analogue, Tre-ADA, utilized in this study. (c) Confocal microscopy images comparing the fluorescence between Tre-ADA-incorporated and vehicle-treated Msmeg strain upon reaction with the CB7-SiR•XYL-BHQ3 complex. (d) Fluorescence image of Tre-ADA-treated *E. coli* postreaction with the CB7-SiR•XYL-BHQ3 complex. (e) Structured illumination microscopy (SIM) images demonstrating the fluorescence signal in Msmeg strain expressing cytoplasmic GFP, after metabolic labeling and reaction with the CB7-SiR•XYL-BHQ3 complex. (f) Confocal microscopy images depicting the difference in fluorescence intensity between Tre-ADA-incorporated and vehicle-treated H37Ra strain upon reaction with the CB7-SiR•XYL-BHQ3 complex. Scale bar: 10 μm (c, d, and f) and 5 μm (e).

One significant advantage of a fluorogenic probe is its application with bioorthogonal groups incorporated using the cell’s own synthesis machinery.^96^ Motivated by the promising outcomes observed in the living samples, we next evaluated whether our supramolecular fluorogenic imaging strategy can be used in combination with metabolic labeling technique. Metabolic incorporation that leverages the trehalose metabolism has recently emerged as an attractive strategy for simultaneously reporting identity and metabolic viability of bacteria belonging to the Mycobacterium genus.^97−100^ Recent findings indicate that modified trehalose analogs can be metabolically incorporated into the mycobacterial outer membrane, referred to as the mycomembrane, in the form of trehalose mycolate.^96−99^ This metabolic processing is facilitated by the substrate promiscuity of the antigen 85 (Ag85) complex, responsible for catalyzing the mycolation of trehalose.^101^ In order to capitalize on this mycobacterial trehalose metabolism, we decided to evaluate whether the mycomembrane can be metabolically labeled with a guest (ADA)-modified trehalose analogue (Tre-ADA) (Figure 8a and 8b), permitting fluorogenic imaging of the Mycobacterium species by the supramolecular fluorogenic probes. To evaluate this, we initially examined the metabolic incorporation and fluorogenic imaging capability using Tre-ADA in the nonpathogenic and fast-growing *Mycobacterium smegmatis (*Msmeg*) mc2155* strain, commonly utilized as an experimental model organism for *Mycobacterium tuberculosis (*Mtb*)*. Growth curves of the Msmeg strain showed no toxicity and perturbation in growth rate in the presence of varying concentrations (100 and 250 μM) of Tre-ADA at 37 °C (Supporting Figure S18). Subsequently, the growth rate of the bacteria in the presence of Tre-ADA was monitored, and upon reaching logarithmic phase, the bacteria were stained with the CB7-SiR•XYL-BHQ3 complex. To our delight, confocal microscopy imaging without washing excess probes revealed concentration-dependent bright fluorescence staining of Msmeg, with minimal background fluorescence from the free probes (Figure 8c). In contrast, when we used untreated Msmeg or a non-Mycobacterium bacterial species (*E. coli*) treated with Tre-ADA, we did not observe any appreciable fluorescence staining of the bacteria after incubation with the CB7-SiR•XYL-BHQ3 complex (Figure 8c and 8d). This finding demonstrates that Tre-ADA specifically targets and labels bacteria belonging to the Mycobacterium genus through metabolic incorporation, with CB7-SiR•XYL-BHQ3 supramolecular complex serving as a fluorogenic reporter probe to enable microscopic investigation of the labeled bacteria. We also used an *Msmeg* strain expressing cytoplasmic green fluorescent protein (GFP) and conducted SIM imaging, which revealed high-contrast fluorescently labeled bacteria under a no-wash condition and proved its compatibility with super-resolution imaging modality (Figure 8e). Importantly, in the SIM microscopy images with enhanced resolution, the fluorescence signal from CB7-SiR was predominantly observed, localizing on the cell surface surrounding the cytoplasmic GFP signal. This finding indicates the expression and localization of Tre-ADA into the mycomembrane. Moreover, the fluorescence signal was often found concentrated at the bacterial poles, a phenomenon previously documented by other researchers and correlated with a polar growth model for mycobacteria.^102^ Finally, we tested this fluorogenic metabolic labeling in H37Ra, an attenuated strain of *M. tuberculosis*. Similar to Msmeg, *M. tuberculosis* H37Ra was metabolically labeled by growing them in 250 μM of Tre-ADA. Subsequent incubation with the CB7-SiR•XYL- BHQ3 probe resulted in fluorogenic staining of H37Ra, whereas control experiments with untreated bacteria resulted in no significant fluorescence signal (Figure 8f). Overall, these observations reveal that unique metabolic labeling routes can be harnessed with our host–guest fluorogenic system to achieve the detection and visualization of metabolically tagged biomolecules under a no wash condition.

**Figure 9:**
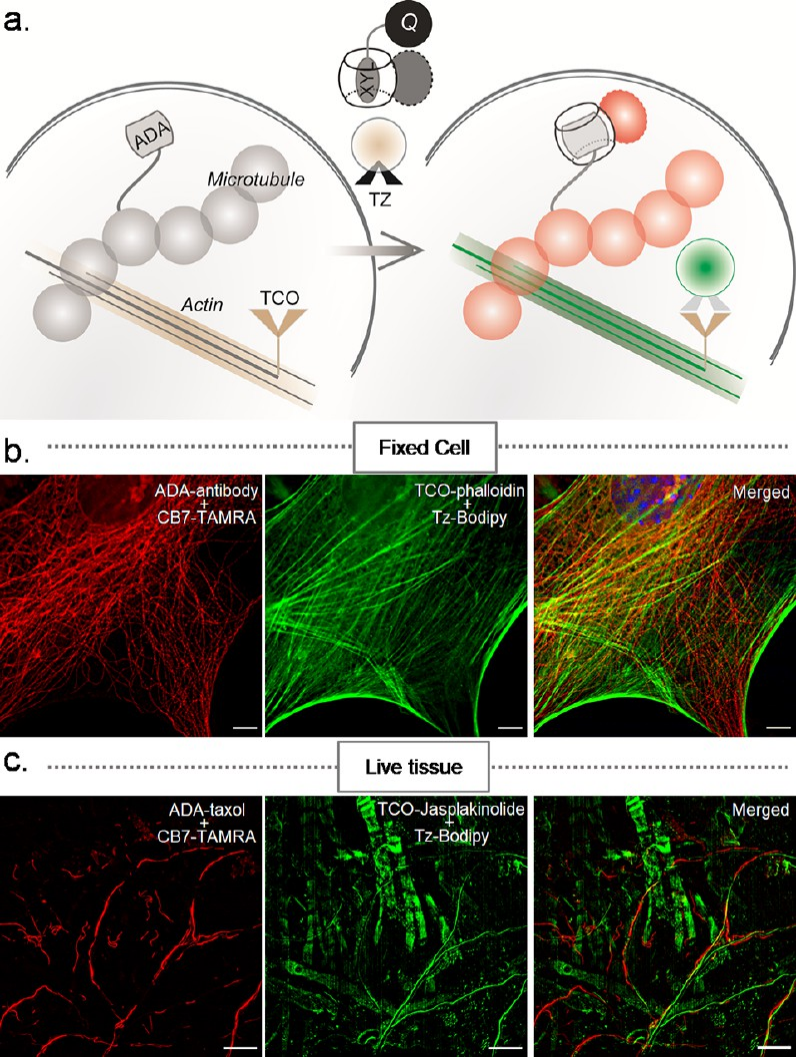
Fluorogenic multiplexed imaging in fixed cells and live tissues using orthogonally reactive fluorogenic probes. (a) Strategy for multiplexed fluorogenic imaging by combining the CB7-based host–guest fluorogenic system with a covalently clickable (TCO–Tz) fluorogenic probe. (b,c) Multiplexed fluorogenic imaging of microtubules and actins in fixed cells and live tissue using orthogonally reactive CB7-TAMRA•XYL-BHQ2 and Tz-Bodipy probes. Scale bar: 10 μm (b,c).

Simultaneous imaging of multiple biomolecules is an important aspect of modern biological investigations, requiring fluorogenic pairs with orthogonal reactivity. The development of our host–guest-based quenched probe presents a new opportunity for multiplexed fluorogenic labeling, as it can be easily paired with the covalently clickable fluorogenic probe. In addition to its orthogonal reactivity, the flexible choice of fluorophores that can be used in the host–guest-based fluorogenic mechanism makes it an ideal choice for multicolor bioorthogonal fluorogenic imaging. To demonstrate this, we used CB7-TAMRA•XYL-BHQ2 based noncovalent fluorogenic probe and combined it with a tetrazine (Tz)-Bodipy-based fluorogenic probe for multiplexed imaging (Figure 9a).^44^ We first aimed to simultaneously image the distribution of microtubule and actin in cells, which were accordingly targeted using ADA-Ab and TCO-phalloidin, respectively. After incubating with a mixture of quenched probes, we acquired dual-color SIM images from the cells. As demonstrated in Figure 9b, the SIM images clearly revealed specific staining of the microtubule network in the TAMRA channel via CB7•ADA complexation, whereas actin filaments showed a strong signal in the Bodipy channel via Tz-TCO reaction. This suggests that orthogonal reactivity of CB7 and Tz based fluorogenic probes can be easily adopted for multicolor imaging experiments without using a clearing step. We further performed dual color fluorogenic imaging of microtubule and actin in live intestine tissue of *Drosophila melanogaster*. Microtubules and actin were targeted using living system compatible small molecule-based targeting agents, docetaxel-ADA and jasplakinolide-TCO conjugates. SIM microscopy images acquired after incubation with the pair of orthogonally reactive fluorogenic probes confirmed distinct staining of the microtubule and actin in live intestine tissue (Figure 9c), highlighting the importance of the host–guest fluorogenic probe in a multiplexed assay.

## Conclusion

In conclusion, we introduced a distinct supramolecular guest exchange strategy based on CB7 host–guest interaction for generating highly efficient bioorthogonal fluorogenic probes. This strategy is highly generalizable and can be easily extended to any readily available fluorophore scaffold without any further core structural alternations, providing straightforward synthesis and appealing optical flexibility for imaging applications. Utilizing this supramolecular guest exchange strategy, we transformed a library of highly optimized dye molecules, spanning the entire visible-light spectrum, into highly efficient fluorogenic probes, validating our approach’s generalizability and robust mechanism. We demonstrated no-wash fluorogenic imaging of diverse target molecules in the biological complexities of live cells and tissue sections, where high contrast images of the target molecules were achieved with minimal background fluorescence and negligible nonspecific signal. We also validated the capability of our supramolecular fluorogenic imaging strategy to synergize with metabolic labeling techniques, enabling the fluorogenic visualization of metabolically tagged biomolecules within living organisms. Further design optimization will focus on developing fluorogenic single-component CB7-dye conjugate. Some attractive CB7-dye conjugates were reported for single component analyte sensing;^74,103^ however, critical design changes would be necessary to reverse their ON-to-OFF response to OFF-to-ON response for fluorogenic imaging applications. Nevertheless, the fast and catalyst-free reactivity, straightforward synthesis, mutual orthogonality to the preexisting bioorthogonal reactions, and appealing optical flexibility of our current host–guest fluorogenic probes should synergize with emerging microscopic methods to open up new opportunities in multiplexed investigations *in vitro*, *in vivo*, and in diagnostic settings. Advancements like genetically encoded unnatural amino acids featuring high-affinity guest side chains could expand the scope of potential multiplexed protein imaging using our developed supramolecular fluorogenic probes. Moreover, because of its highly flexible choice of fluorophores unmatched by other techniques, the current strategy can easily accommodate already existing highly specialized dye molecules optimized for super-resolution imaging, providing an important addition to the toolkit of fluorogenic nanoscopic imaging.^2,3,104,105^

## Supporting Information Available

Synthesis and characterization data of the host–guest probes and targeting ligands. Experimental protocols for imaging, microscopy, analysis, and other supporting data (PDF)
Transparent Peer Review report available (PDF)
