# Supplementary material for: Supramolecular Guest Exchange in Cucurbit[7]uril for Bioorthogonal Fluorogenic Imaging across the Visible Spectrum
Source: ACS Cent Sci. 2024 Oct 8;10(10):1945–59. doi: 10.1021/acscentsci.4c01080 (PMC11503495; doi:10.1021/acscentsci.4c01080)
Supplement: Supplementary file 1 — oc4c01080_si_001.pdf [file oc4c01080_si_001.pdf]

Supporting information

for

Supramolecular guest exchange in cucurbit[7]uril for bioorthogonal fluorogenic imaging across the visible spectrum

Ranjan Sasmal, [a,‡] Arka Som, [a,‡] Pratibha Kumari,[a] Resmi V. Nair,[a] Sushanta Show,[a] Nisha Sanjay Barge,[b] Meenakshi Pahwa,[a] Nilanjana Das Saha,[a] Sushma Rao,[c] Sheeba Vasu,[c] Rachit Agarwal,[b] and Sarit S. Agasti[a]*

[a]New Chemistry Unit, Chemistry & Physics of Materials Unit, and School of Advanced Materials (SAMat), Jawaharlal Nehru Centre for Advanced Scientific Research (JNCASR), Bangalore, Karnataka 560064, India.

[b]Department of Bioengineering, Indian Institute of Science, Bengaluru 560012, Karnataka, India

[c]Evolutionary and Integrative Biology Unit and Neuroscience Unit, Jawaharlal Nehru Centre for Advanced Scientific Research (JNCASR), Bangalore, Karnataka 560064, India.

‡R.S. and A.S. contributed equally to this work.

Corresponding author

Correspondence to: Sarit S. Agasti

E-mail: sagasti@jncasr.ac.in

https://orcid.org/0000-0001-7216-0490

Table of Contents:

Contents	Page Number
1. General information	S3
2. Synthesis protocol	S5
3. Preparation of targeting conjugates	S14
4. Synthetic of XYL decorated gold nanoparticle (XYL–AuNP)	S18
5. Synthetic protocol for Tre-ADA	S19
6. Fluorescence titration and MALDI-MS experiments	S21
7. Labelling experiment with in vitro polymerized microtubule	S27
8. Protocol for fluorogenic labelling in cells, tissue and metabolically labeled bacteria	S29
9. Microscopy setup	S38
10. Image processing and data analysis	S40
11. Protocol for kinetic study in the cell using confocal imaging.	S42
12. Supporting information images	S43
13. HPLC, Mass, and NMR characterization data	S52
14. References	S70

1. General information

All the chemicals were purchased from either of the following companies: Sigma Aldrich, Alfa Aesar, Thermo Fischer Scientific, TCI Chemicals, Merck, SD fine chemicals, and Spectrochem, unless mentioned specifically. Amino Phalloidin (Product No. 92-1-10) was purchased from the American Peptide Company. Lysine derivative of jasplakinolide (Boc-Lys-jasplakinolide, SKU – SC005) was purchased from Spirochrome Ltd. Phosphate buffered saline powder pH 7.4 (Product No. P3813-10PAK) was purchased from Sigma-Aldrich. Zeba™ spin desalting columns (Product No. 89883) were purchased from Thermo Fisher Scientific. Antibodies, fluorophores, and quenchers were purchased from commercial sources as listed below. Whenever necessary, solvents were dried by using standard solvent drying methods and then used for reactions. ^{1}H NMR spectrum was recorded using Bruker AVANCE III 400 MHz and JEOL Delta 600 MHz instrument, and data analysis was done using Spinworks_4.0 and JEOL delta v5.0.5.1 software. High-Resolution Mass Spectrometry (HRMS) was carried out using Agilent 6538 Ultra High Definition (UHD) Accurate-Mass Q-TOF LC/MS. Liquid chromatography-mass spectrometry (LCMS) experiments were carried out using a Waters Alliance High-Performance Liquid Chromatography (HPLC) system attached a SQD2 mass detector. HPLC purification was carried out using Agilent 1260 infinity quaternary HPLC system equipped with analytical ZORBAX Eclipse plus C18 column (4.6 mm × 100 mm, 3.5 microns) and semipreparative ZORBAX Eclipse plus C18 column (9.4 mm × 250 mm, 5 microns). The solvents used as eluent in HPLC purification were solvent A (water containing 0.1% TFA) and solvent B (acetonitrile containing 0.1% TFA). In general, gradient elution of solvent B in solvent A from 5-100% was used for the purification. Absorbance measurement was carried out to check the concentration of fluorophores in an Eppendorf BioSpectrometer. The microscopic studies were carried out using three optical set-ups: a) A custom-built inverted epi–fluorescence microscope (Olympus) equipped with Cool–LED light source, 2) A Zeiss ELYRA PS1 set up for Structured Illumination Microscopy (SIM), and 3) A Leica SP8 confocal microscope.

Table S1: List of primary antibodies used for immunostaining.

Target	Antibody commercial sources	Species
Microtubule (anti–α tubulin)	Thermo Fischer Scientific (MA1-80017)	Rat
Human EGFR (Cetuximab) MAb (Clone Hu1)	R&D Systems, Inc (MAB9577-100)	Human

Human EGFR (Cetuximab)	Merck (IL/BIO-000085-FF-373)	Human
Anti-NUP98 antibody [2H10] - Nuclear Pore Marker	Abcam (ab50610)	Rat

Table S2: List of secondary antibodies used for immunostaining.

Target	Host	Specification and commercial source
Rat	Donkey	Donkey Anti-Rat IgG (H+L)<br>(min X Bov, Ck, Gt, GP, SyHms, Hrs, Hu, Ms, Rb, Shp Sr Prot)<br>Jackson ImmunoResearch Laboratories (Cat. No. 712-005-153)
Human	Donkey	Donkey Anti-Human IgG (H+L)<br>(min X Bov, Ck, Gt, GP, Sy Hms, Hrs, Ms, Rb, Rat, Shp Sr Prot)<br>Jackson ImmunoResearch Laboratories (Cat. No. 709-005-149)

Table S3: List of fluorophores used for the fluorogenic imaging study.

Fluorophores	Commercial source
Coumarin NHS ester	TCI Chemicals (Cat. No. S0866)
Fluorescein isothiocyanate	TCI Chemicals (Cat. No. F0026)
Bodipy NHS ester	TOCRIS Bioscience (Cat. No. 5465)
TAMRA NHS ester	Sigma Aldrich (Cat. No. 53048)
Cy3 NHS ester	Lumiprobe (Cat. No. 21020)
Cy5 NHS ester	Lumiprobe (Cat. No. 23020)
Silicon rhodamine (SiR) NHS ester	Spirochrome (SC003)
Alexa488-Phalloidin	Thermo Fisher Scientific (A12379)
Alexa568-NHS	Thermo Fisher Scientific (A20103)

Table S4: List of Quenchers used for the fluorogenic imaging study.

Quenchers	Commercial source
Dabcyl NHS ester	Sigma Aldrich (Cat. No. 09278)
BHQ1 NHS ester	LGC Biosearch Technologies (Cat No. BHQ–1000S)
BHQ2 NHS ester	LGC Biosearch Technologies (Cat No. BHQ–2000S)
BHQ3 NHS ester	LGC Biosearch Technologies (Cat No. BHQ–3000S)

2. Synthesis protocol

2.1 Synthesis protocol for functionalized guest molecules

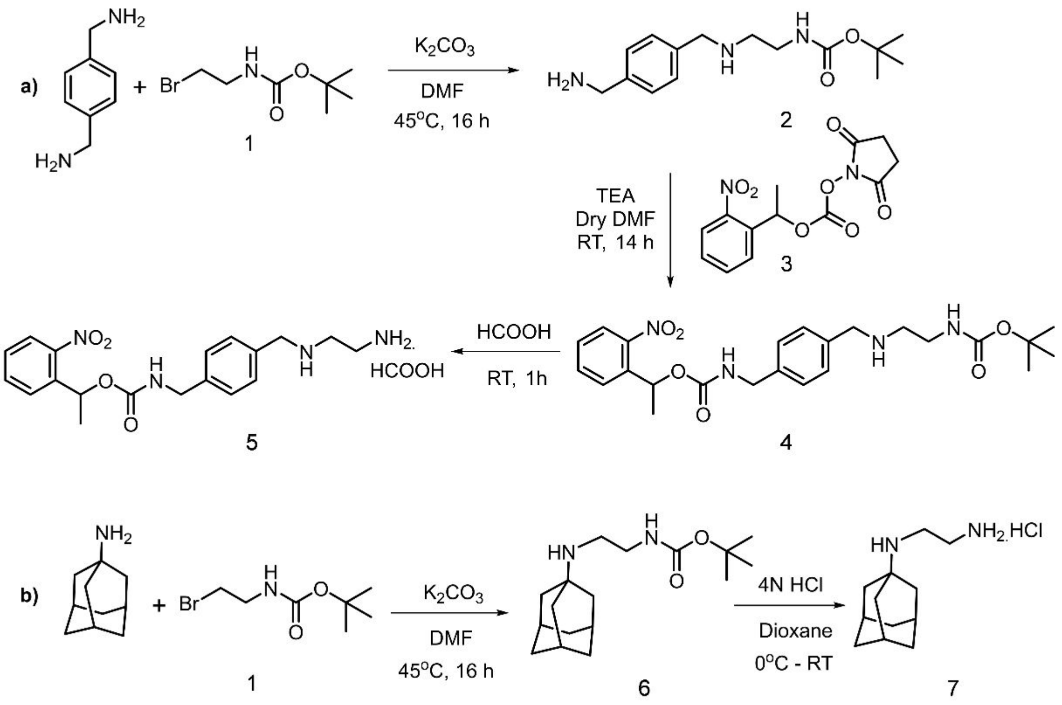

Scheme S1: Synthetic scheme for preparing amine derivatives of a) XYL and b) ADA guests.

2.1.1 Synthesis of Compound 1

Compound 1 has been synthesized according to the literature procedure.[1]

2.1.2 Synthesis of Compound 2

In a 50 ml RB flask, *p*–xylenediamine (3 g, 22.027 mmol) was dissolved in 15 ml dry dimethyl formamide (DMF) followed by the addition of potassium carbonate (K_2CO_3) (1.5 g, 10.85 mmol) to the solution. In a separate flask compound 1 (0.8 g, 3.587 mmol) was dissolved in 5 ml dry DMF. The solution was added dropwise to *p*-xylenediamine solution over 10 min. The reaction mixture was stirred at 45˚C for 16 h and then cooled down to room temperature. The thin layer chromatography (TLC) was checked in 20% methanol (MeOH) in dichloromethane (DCM) (R_f = 0.09) to ensure the completion of the reaction. After that, the reaction mixture was filtered to remove solids, and the filtrate was extracted in DCM by washing with water (2 times), brine solution (1 time), and concentrated under reduced pressure. The crude sample was charged on silica gel and purified by column chromatography (eluent: 20% MeOH, 4% NH_3 in DCM). Compound 2 was thus obtained as a yellow color liquid (370 mg, 1.324 mmol, Yield 37%). LCMS (ESI–MS): calculated 280.20 $[M+H]^+$, found 280.11 $[M+H]^+$. ^1H–NMR (400 MHz, CD_3OD): δ

7.31 (d, 4H, ArH), 3.79 (s, 2H, benzyl–CH_2–), 3.73 ((s, 2H, benzyl–CH_2–), 3.18 (t, 2H, CO–NH–CH_2), 2.65 (t, 2H, NH–CH_2), 1.42 (s, 9H, –$C(CH_3)_3$–). ^{1}H–NMR of compound 2 has been shown in Figure S33.

2.1.3 Synthesis of Compound 4

Compound 3 has been synthesized according to the literature procedure[2]. In a 5 ml pear-shaped flask, compound 2 (100 mg, 0.357 mmol) was taken, and an inert atmosphere was created using N_2 gas. To this, 2 ml of dry DMF was added, followed by subsequent addition of triethylamine (Et_3N) (0.120 ml, 0.892 mmol). At last, compound 3 (121 mg, 0.393 mmol) was added. The reaction mixture was stirred at room temperature (RT) for 14 h. The TLC was checked in 5% MeOH in DCM (R_f = 0.21). The solvent was evaporated under reduced pressure and dissolved in DCM. The organic layer was washed with water (2 times), brine solution (1 time), and dried over sodium sulfate (Na_2SO_4). The crude sample was charged on silica gel and purified by column chromatography (eluent: 5% MeOH in DCM). Compound 4 was thus obtained as a colorless oily liquid (82 mg, 0.173 mmol, Yield 49%). LCMS (ESI–MS): calculated 473.24 $[M+H]^+$, found 473.67 $[M+H]^+$. ^{1}H–NMR (400 MHz, $CDCl_3$): δ 7.92 (d, 1H, ArH), 7.63 (m, 2H, ArH), 7.43 (m, 1H, ArH), 7.29 (d, 2H, ArH), 7.20 (d, 2H, ArH), 6.29 (q, 1H, O–CH–), 5.13 (br, 1H, NH–CO), 5.05 (br, 1H, NH–BOC), 4.30 (s, 2H, benzyl–CH_2– NH–CO), 3.79 (s, 2H, benzyl–CH_2– NH), 3.24 (t, 2H, CO–NH–CH_2–), 2.77 (t, 2H, NH– CH_2–),1.64 (d, 3H, –C–CH_3), 1.44 (s, 9H, –$C(CH_3)_3$–). ^{1}H–NMR of compound 4 has been shown in Figure S34.

2.1.4 Synthesis of Compound 5

In a 5 ml pear-shaped flask, compound 4 (54.6 mg, 0.116 mmol) was dissolved in formic acid (5.9 ml, 156.42 mmol) and incubated at room temperature for 1 h. The solvent was evaporated under reduced pressure and thoroughly dried under a high vacuum. The product was dissolved in a minimum amount of acetonitrile (ACN): water (1:1) and lyophilized. Compound 5 was thus obtained as a yellow color liquid (17.1 mg, 0.0459 mmol, Yield 87%). HRMS (ESI–MS): calculated 373.1870 $[M + H]^+$, found 373.1854 $[M + H]^+$. ^{1}H–NMR (400 MHz, $CDCl_3$): δ 8.26 (br, 1H, formate) 7.86 (d, 1H, ArH), 7.60 (m, 2H, ArH), 7.36 (m, 1H, ArH), 7.22 (d, 2H, ArH), 7.10 (d, 2H, ArH), 6.19 (q, 1H, O–CH–), 6.10 (br, 1H, NH–CO), 4.13 (s, 2H, benzyl–CH_2– NH–CO), 3.79 (s, 2H, benzyl–CH_2– NH), 2.97 (t, 4H, –CH_2–CH_2–),1.56 (d, 3H, –C–CH_3). ^{1}H–NMR of compound 5 has been shown in Figure S35.

2.1.5 Synthesis of Compound 6

In a 25 ml round bottom (RB) flask, 1–adamantylamine (1.0 g, 6.60 mmol) was dissolved in 5 ml dry DMF followed by the addition of K_2CO_3 (1.11 g, 8.031 mmol) to the solution. In a separate flask compound 1 (1.48 g, 6.60 mmol) was dissolved in 3 ml dry DMF and added to 1–adamantylamine

solution. The reaction mixture was stirred at 45°C for 16 h and then cooled down to room temperature. The TLC was checked in 20% MeOH in DCM (R_f = 0.2). After that, the reaction mixture was filtered to remove the solids, and the filtrate was extracted in DCM by washing with water (2 times), brine solution (1 time), and concentrated under reduced pressure. The crude sample was charged on silica gel and purified by column chromatography (eluent: 20% MeOH, 1% NH_3 in DCM). Compound 6 was thus obtained as a yellow color liquid (675 mg, 2.310 mmol, Yield 35%). ^{1}H–NMR (400 MHz, $CDCl_3$): δ 3.50 (Br, 2H, –CH_2– NHCO–), 3.27 (Br, 2H, –NH–CH_2–), 2.83 (s, 1H, –NH–CO–), 2.10 (t, 3H, –CH–), 1.64–1.72 (m, 12H, –CH_2–), 1.44 (s, 9H, –$(CH_3)_3$). ^{1}H–NMR of compound 6 has been shown in Figure S36.

2.1.6 Synthesis of Compound 7

In a 5 ml pear-shaped flask, 4N hydrochloric acid (HCl) in dioxane (2 ml, 156.42 mmol) was added to compound 6 (400 mg, 1.36 mmol) at 0°C. The reaction mixture was incubated at room temperature for 1 h. The solvent was evaporated under reduced pressure and thoroughly dried under a high vacuum. Afterward, the product was dissolved in a minimum amount of ACN: water (1:1) and lyophilized. Compound 7 was thus obtained as yellow colored solid (229 mg, 1.18 mmol, Yield 87%). ^{1}H–NMR (400 MHz, D_2O): δ 3.40 (Br, 4H, –CH_2– NH–), 2.98 (s, 1H, – NH–), 2.27 (Br, 3H, –CH–), 1.97 (Br, 6H, –CH_2–), 1.80 (m, 6H, –CH_2–). ^{1}H–NMR of compound 7 has been shown in Figure S37.

2.2 Synthesis protocol for XYL conjugated quenchers (XYL-Q)

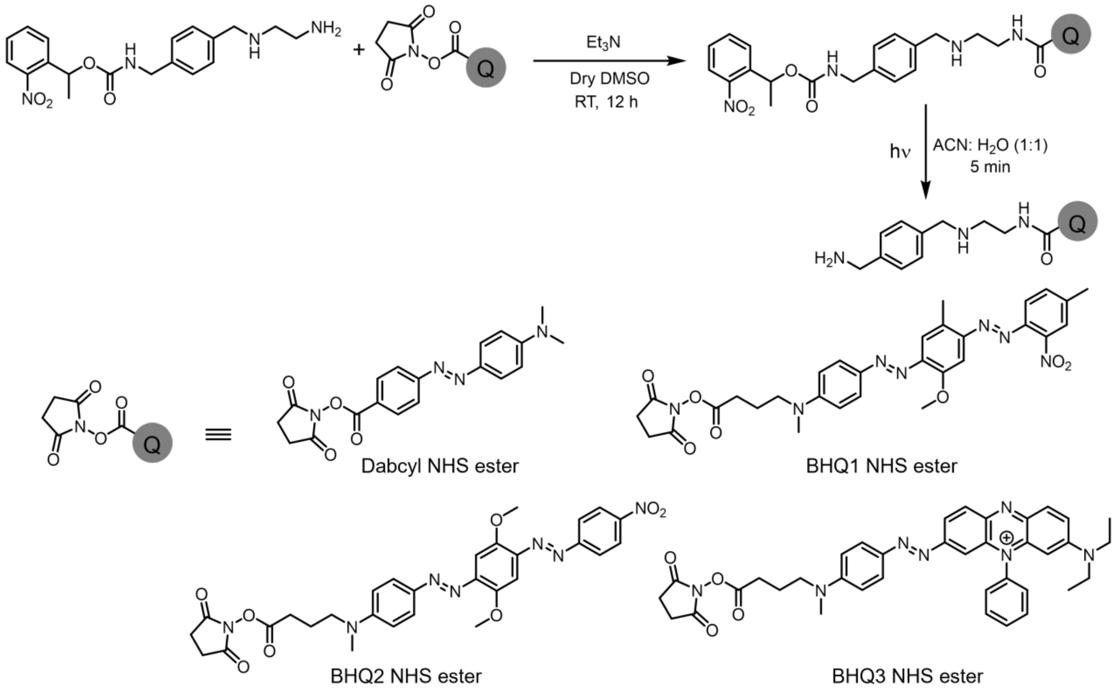

Scheme S2: Synthetic scheme for the preparation of XYL–Q.

2.2.1 Synthesis of XYL conjugated Dabcyl

Compound 5 (305 mg, 30.5 ml from 10 mg. ml^{-1} stock in dry Dimethyl sulfoxide (DMSO), 0.819 mmol) was taken in a microcentrifuge tube and triethylamine (138 mg, 1.9 ml from 100 ml.ml^{-1} stock in dry DMSO, 1.365 mmol) was added to it. Dabcyl NHS ester (100 mg, 10 ml from 10 mg. ml^{-1} stock in dry DMSO, 0.273 mmol) was added to the above mixture, and the reaction was stirred at room temperature for 12 h. Next, the reaction mixture was made into a 1:1 mixture in water and directly injected into HPLC for purification using water/acetonitrile as eluent. The purified product was characterized by LCMS and dried by lyophilization. The yield calculated is ~75 % (from HPLC). LCMS (ESI–MS): calculated 624.29 $[M+H]^+$, 312.65 $[M+2H]^{2+}$; found 624.38 $[M+H]^+$, 312.85 $[M+2H]^{2+}$.

The photocleavable group (PCG) protected xylene (XYL) conjugated product was further dissolved in 1:1 mixture of water/acetonitrile and irradiated with 365 nm UV lamp (50 mW) for 5 min to photocleave the PCG group followed by purified by HPLC using water/acetonitrile as eluent. The purified product was characterized by ^{1}H–NMR and HRMS. The yield calculated is ~90% (from HPLC). HRMS (ESI–MS): calculated 431.2554 $[M+H]^+$; found 431.2565 $[M+H]^+$. ^{1}H–NMR (DMSO–d^6, 600 MHz) δ 8.88 (s, 1H), 8.80 (t, J = 5.7 Hz, 1H), 8.15 (s, 2H), 7.99 (d, J = 8.6 Hz, 2H), 7.85 (d, J = 8.6 Hz, 2H), 7.81 (d, J = 8.9 Hz, 2H), 7.54 (d, J = 7.9 Hz, 2H), 7.50 (d, J = 7.9 Hz, 2H), 6.85 (d, J = 9.3 Hz, 2H), 4.24 (t, J = 5.7 Hz, 2H), 4.06 (q, J = 5.8 Hz, 2H), 3.61 (q, J = 5.9 Hz, 2H), 3.13 (t, J = 6.0 Hz, 2H), 3.08 (s, 6H). The HPLC chromatogram and ^{1}H–NMR of XYL–Dabcyl have been shown in Figure S20 and S38, respectively.

2.2.2 Synthesis of XYL conjugated BHQ1

Compound 5 (310 mg, 31 ml from 10 mg. ml^{-1} stock in dry DMSO, 0.8331 mmol) was taken in a microcentrifuge tube and triethylamine (84 mg, 1.2 ml from 100 ml.ml^{-1} stock in dry DMSO, 0.8331 mmol) was added to it. BHQ1 NHS ester (100 mg, 10 ml from 10 mg. ml^{-1} stock in dry DMSO, 0.1662 mmol) was added to the above mixture, and the reaction was stirred at room temperature for 12 h. Next, the reaction mixture was made into a 1:1 mixture in water and directly injected into HPLC for purification using water/acetonitrile as eluent. The purified product was characterized by LCMS and dried by lyophilization. The yield calculated is ~60% (from HPLC). LCMS (ESI–MS): calculated 859.39 $[M+H]^+$, 430.19 $[M+2H]^{2+}$; found 859.58 $[M+H]^+$, 430.39 $[M+2H]^{2+}$.

The PCG protected XYL conjugated product was then dissolved in 1:1 mixture of water/acetonitrile and irradiated with 365 nm UV lamp (50 mW) for 5 min to photocleave the PCG group followed by purified by HPLC using water/acetonitrile as eluent. The purified product was characterized by ^{1}H–NMR and HRMS. The yield calculated is ~95% (from HPLC). HRMS (ESI–MS): calculated 666.3511 $[M+H]^+$, 333.6792 $[M+2H]^{2+}$; found 666.3310 $[M+H]^+$, 333.6763 $[M+2H]^{2+}$. ^{1}H–NMR (DMSO–d^6, 600 MHz) δ 8.81

(s, 2H), 8.13 (t, J = 5.8 Hz, 3H), 7.95 (s, 1H), 7.81 (d, J = 9.9 Hz, 2H), 7.77 (d, J = 8.2 Hz, 1H), 7.69 (d, J = 7.2 Hz, 1H), 7.51 (dd, J = 15.6, 8.4 Hz, 4H), 7.29 (s, 1H), 6.88 (d, J = 9.3 Hz, 2H), 4.20 (t, J = 5.8 Hz, 2H), 4.05 (q, J = 5.8 Hz, 2H), 3.92 (s, 3H), 3.43-3.66 (m, 4H merged with H_2O peak), 3.06 (s, 3H), 2.97 (t, J = 6.0 Hz, 2H), 2.63 (s, 3H), 2.55-2.38 (m, 3H merged with DMSO peak), 2.20 (t, J = 7.4 Hz, 2H), 1.78-1.83 (m, 2H). The HPLC chromatogram and ^{1}H–NMR of XYL–BHQ1 have been shown in Figure S21 and S39, respectively.

2.2.3 Synthesis of XYL conjugated BHQ2

Compound 5 (308 mg, 30.8 ml from 10 mg. ml^{-1} stock in dry DMSO, 0.8283 mmol) was taken in a microcentrifuge tube and triethylamine (83.65 mg, 1.2 ml from 100 ml.ml^{-1} stock in dry DMSO, 0.8283 mmol) was added to it. BHQ2 NHS ester (100 mg, 10 ml from 10 mg. ml^{-1} stock in dry DMSO, 0.1657 mmol) was added to the above mixture, and the reaction was stirred at room temperature for 12 h. Next, the reaction mixture was made into a 1:1 mixture in water and directly injected into HPLC for purification using water/acetonitrile as eluent. The purified product was characterized by LCMS and dried by lyophilization. The yield calculated is ~90% (from HPLC). LCMS (ESI–MS): calculated 861.36 $[M+H]^+$, 431.19 $[M+2H]^{2+}$; found 861.54 $[M+H]^+$, 431.31 $[M+2H]^{2+}$.

The PCG protected XYL conjugated product was then dissolved in 1:1 mixture of water/acetonitrile and irradiated with 365 nm UV lamp (50 mW) for 5 min to photocleave the PCG group followed by purified by HPLC using water/acetonitrile as eluent. The purified product was characterized by ^{1}H–NMR and HRMS. The yield calculated is ~100% (from HPLC). HRMS (ESI–MS): calculated 668.3303 $[M+H]^+$, 334.6688 $[M+2H]^{2+}$; found 668.3310 $[M+H]^+$, 334.6690 $[M+2H]^{2+}$. ^{1}H–NMR (DMSO–d^6, 600 MHz) δ 8.78 (s, 2H), 8.44 (d, J = 8.9 Hz, 2H), 8.11 (t, J = 5.7 Hz, 3H), 8.06 (d, J = 8.9 Hz, 2H), 7.82 (d, J = 8.9 Hz, 2H), 7.49 (q, J = 8.0 Hz, 4H), 7.37 (s, 1H), 6.88 (d, J = 9.3 Hz, 2H), 4.18 (t, J = 5.8 Hz, 2H), 4.04 (q, J = 5.8 Hz, 2H), 3.99 (s, 3H), 3.94 (s, 3H), 3.51-3.39 (m, 2H merged with H_2O peak), 3.06 (s, 3H), 2.96 (t, J = 6.2 Hz, 2H), 2.59-2.39 (m, 2H merged with DMSO peak), 2.19 (t, J = 7.4 Hz, 2H), 1.80 (t, J = 7.2 Hz, 2H). The HPLC chromatogram and ^{1}H–NMR of XYL–BHQ2 have been shown in Figure S22 and S40, respectively.

2.2.4 Synthesis of XYL conjugated BHQ3

Compound 5 (288.84 mg, 28.9 ml from 10 mg. ml^{-1} stock in dry DMSO, 0.7754 mmol) was taken in a microcentrifuge tube and triethylamine (78.3 mg, 1.1 ml from 100 ml.ml^{-1} stock in dry DMSO, 0.7754 mmol) was added to it. BHQ3 NHS ester (100 mg, 10 ml from 10 mg. ml^{-1} stock in dry DMSO, 0.1550 mmol) was added to the above mixture, and the reaction was stirred at room temperature for 12 h. Next, the reaction mixture was made into a 1:1 mixture in water and directly injected into HPLC for purification using water/acetonitrile as eluent. The purified product was characterized by LCMS and

dried by lyophilization. The yield calculated is ~40% (from HPLC). LCMS (ESI–MS): calculated 901.45 $[M]^+$, 451.23 $[M+H]^{2+}$; found 901.64 $[M]^+$, 451.38 $[M+H]^{2+}$.

The PCG protected XYL conjugated product was then dissolved in 1:1 mixture of water/acetonitrile and irradiated with 365 nm UV lamp (50 mW) for 5 min to photocleave the PCG group followed by purified by HPLC using water/acetonitrile as eluent. The purified product was characterized by ^{1}H–NMR and HRMS. The yield calculated is ~99% (from HPLC). HRMS (ESI–MS): calculated 708.4133 $[M]^+$, 354.7103 $[M+H]^{2+}$; found 708.4136 $[M]^+$, 354.7105 $[M+H]^{2+}$. ^{1}H–NMR (DMSO–d^6, 600 MHz) δ 8.84-8.76 (2H), 8.43 (d, J = 8.9 Hz, 2H), 8.19-8.21 (m, 2H), 8.15 (s, 3H), 8.00 (d, J = 12.3 Hz, 1H), 7.90-7.95 (m, 2H), 7.77-7.80 (m, 3H), 7.50 (d, J = 5.5 Hz, 4H), 7.23 (s, 1H), 6.88 (d, J = 9.3 Hz, 2H), 5.70 (s, 1H), 4.18-4.20 (m, 2H), 4.04-4.07 (m, 2H), 3.56-3.82 (m, 6H merged with H_2O peak), 3.09 (s, 3H), 2.95-2.99 (m, 2H), 2.41-2.65 (m, 2H merged with DMSO peak), 2.19 (t, J = 6.7 Hz, 2H), 1.77-1.81 (m, 2H), 1.23 (s, 6H). The HPLC chromatogram and ^{1}H–NMR of XYL–BHQ3 have been shown in Figure S23 and S41, respectively.

2.3 Synthesis protocol for EtA conjugated quenchers (EtA-Q)

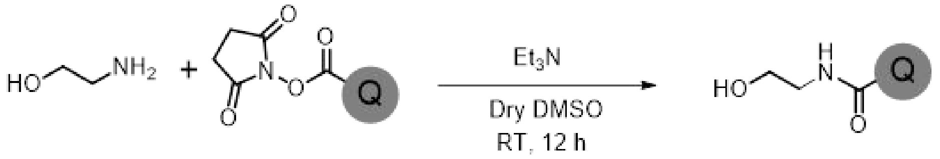

Scheme S3: Synthetic scheme for the preparation of EtA–Q.

2.3.1 Synthesis of EtA–Dabcyl conjugate

Ethanolamine (166.67 mg, 1.67 ml from 1:10 diluted stock in dry DMSO, 2.7295 mmol) was taken in a microcentrifuge tube and triethylamine (1.9 ml from 1:10 diluted stock in dry DMSO, 1.3647 mmol) was added to it and mixed by vortex mixture. Dabcyl NHS ester (100 mg, 0.2729 mmol) was added to the above mixture and the reaction was stirred at room temperature for 12 h. Next, the reaction mixture was diluted to a 1:1 mixture in water and directly injected into HPLC for purification (Figure S24) using water/acetonitrile as eluent. The purified compound was characterized by HRMS. The yield calculated is ~95% (from HPLC). HRMS (ESI–MS): calculated 313.1659 $[M+H]^+$; found 313.1649 $[M+H]^+$.

2.3.2 Synthesis of EtA–BHQ1 conjugate

Ethanolamine (101.20 mg, 1.02 ml from 1:10 diluted stock in dry DMSO, 1.6620 mmol) was taken in a microcentrifuge tube and triethylamine (1.16 ml from 1:10 diluted stock in dry DMSO, 0.8283 mmol) was added to it and mixed by vortex mixture. BHQ1 NHS ester (100 mg, 0.1662 mmol) was added to the above mixture and the reaction was stirred at room temperature for 12 h. Next, the reaction mixture was diluted to a 1:1 mixture in water and directly injected into HPLC for purification (Figure S25) using

water/acetonitrile as eluent. The purified compound was characterized by HRMS. The yield calculated is ~95% (from HPLC). HRMS (ESI–MS): calculated 548.2616 $[M+H]^+$; found 548.2604 $[M+H]^+$.

2.3.3 Synthesis of EtA–BHQ2 conjugate

Ethanolamine (101.20 mg, 1.02 ml from 1:10 diluted stock in dry DMSO, 1.657 mmol) was taken in a microcentrifuge tube and triethylamine (1.16 ml from 1:10 diluted stock in dry DMSO, 0.8283 mmol) was added to it and mixed by vortex mixture. BHQ2 NHS ester (100 mg, 0.1657 mmol) was added to the above mixture and the reaction was stirred at room temperature for 12 h. Next, the reaction mixture was diluted to a 1:1 mixture in water and directly injected into HPLC for purification (Figure S26) using water/acetonitrile as eluent. The purified compound was characterized by HRMS. The yield calculated is ~95% (from HPLC). HRMS (ESI–MS): calculated 550.2409 $[M+H]^+$; found 550.2388 $[M+H]^+$.

2.3.4 Synthesis of EtA–BHQ3 conjugate

Ethanolamine (77.8 mg, 7.78 ml from 1:100 diluted stock in dry DMSO, 1.2660 mmol) was taken in a microcentrifuge tube and triethylamine (8.8 ml from 1:100 diluted stock in dry DMSO, 0.6333mmol) was added to it and mixed by vortex mixture. BHQ3 NHS ester (100 mg, 0.1551 mmol) was added to the above mixture and the reaction was stirred at room temperature for 12 h. Next, the reaction mixture was diluted to a 1:1 mixture in water and directly injected into HPLC for purification (Figure S27) using water/acetonitrile as eluent. The purified compound was characterized by HRMS. The yield calculated is ~60% (from HPLC). HRMS (ESI–MS): calculated 590.3238 $[M]^+$; found 590.3214 $[M]^+$.

2.4. Synthesis protocol for tetrazine conjugated Bodipy fluorophore

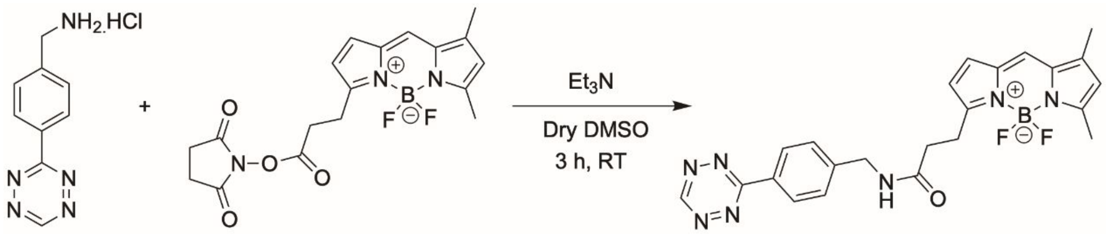

Scheme S4: Synthetic scheme for preparing tetrazine–BODIPY conjugate.

Tetrazine-Bodipy was synthesized using the previously reported protocol[3]. Benzyl amine derivative of tetrazine (241.2 mg, 24.12 ml from 10 mg. ml^{-1} stock in DMSO, 1.2848 mmol) was taken in a microcentrifuge tube. Triethylamine (259.57 mg, 3.6 ml from 1:10 (v/v) stock in DMSO, 2.5700 mmol) was added to it. Bodipy NHS ester (100 mg, 5 ml from 10 mg. ml^{-1} stock in DMSO, 0.2570 mmol) was added to the reaction mixture and stirred at room temperature for 3 h. After that, the reaction mixture was diluted using water and directly injected into HPLC for purification. The purified product was dried

under vacuum and characterized by LCMS. The yield calculated is ~50% (from HPLC). LCMS (ESI–MS): calculated 462.20 $[M+H]^+$; found 462.47 $[M+H]^+$.

2.5. Synthesis of NHS ester derivatives of ADA

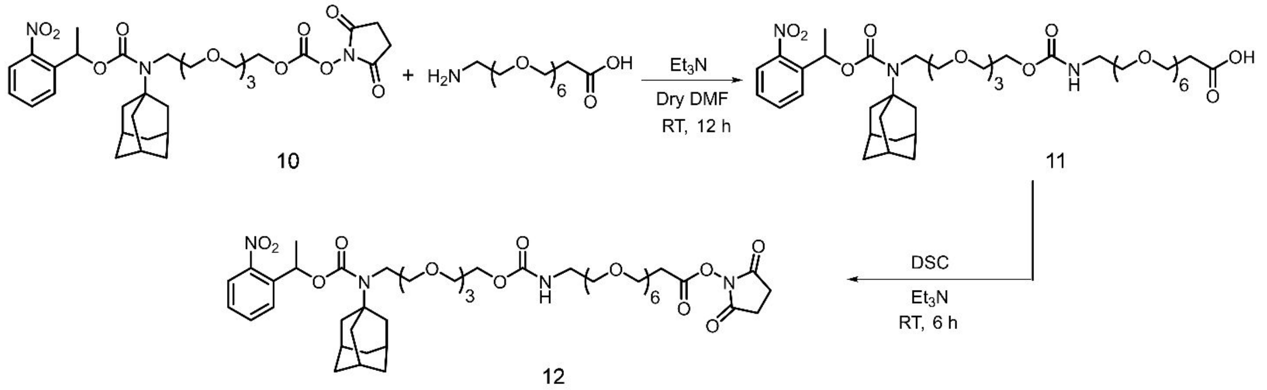

Scheme S5: Synthetic scheme for the preparation of nitrobenzyl-protected ADA–PEG–NHS ester (Compound 12, ADAPc-PEG-NHS ester).

Note – Compounds 11 and 12 were synthesized using our previously reported protocol.[4]

2.6. Synthesis of PC_2XYL-PEG-NHS compound for antibody conjugation

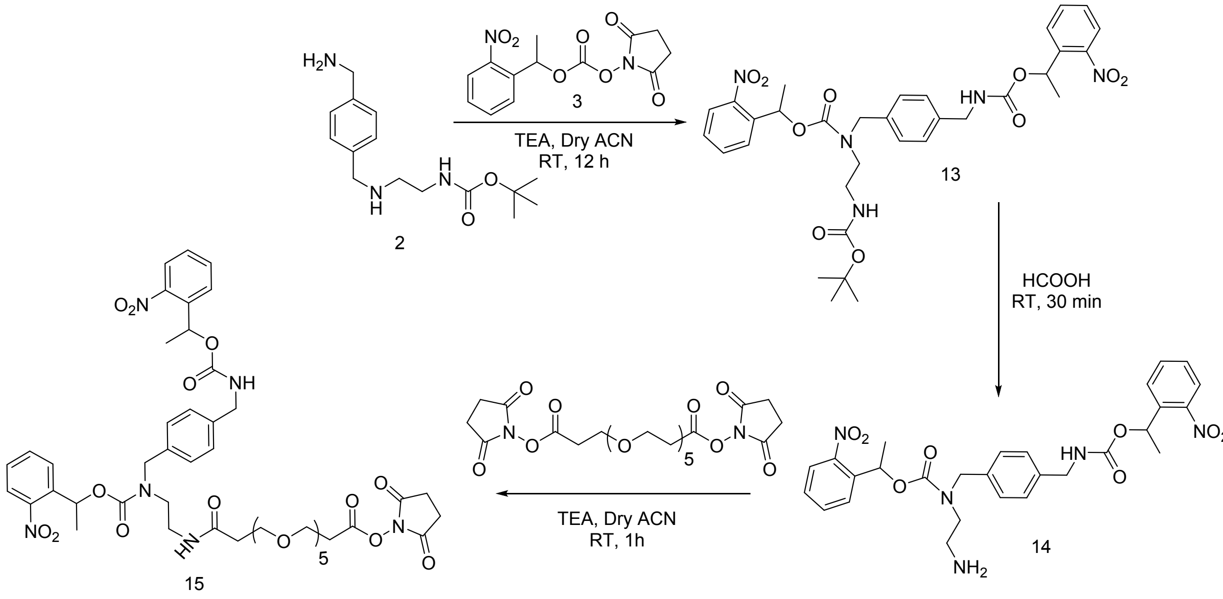

2.6.1 Synthesis of Compound 13: Compound 2 (100.0 mg, 0.358 mmol) was taken in a 50 ml RB flask under an argon inert atmosphere at room temperature. 10.0 ml of dry acetonitrile and triethylamine (0.22 ml, 2.148 mmol) were added sequentially. The mixture was kept under stirring and Compound 3 (0.50 g, 1.611 mmol) was added to it and kept for 12 h at room temperature. Upon completion of the reaction (monitored by TLC), the reaction mixture was diluted with 10 ml water, washed with water (2 ×), HC1 solution (1M, 2 ×), and extracted with dichloromethane (DCM) (3 ×). The combined organic

layers were dried over Na_2SO_4 and solvent was removed under vacuum. The crude product was purified by silica column chromatography (eluent: DCM/MeOH) to yield Compound 13 (340.0 mg, 71.4 %). R_f = 0.25 (DCM). LCMS (ESI-MS): calculated m/z 666.28 $[M+H]^+$; found 666.53 $[M+H]^+$. ^1H NMR (400 MHz, DMSO-d_6): δ 7.95-7.55 (8H, m, ArH), 7.11 (4H, m, ArH), 6.00 (2H, q, −CH−), 4.34 (2H, m, $-CH_2-$), 4.07 (2H, m, $-CH_2-$), 3.17 (4H, t, $-CH_2-$), 3.04 (2H, br, −NH−), 1.55 (6H, d, $-CH_3$), 1.36 (9H, s, $-CH_3$). ^1H NMR spectra of PC_2XYL-NHBoc have been shown in Figure S42.

2.6.2 Synthesis of Compound 14: Compound 13 (340.0 mg, 0.510 mmol) was dissolved in 23 ml of formic acid and allowed to stir at room temperature for 30 min. Then the reaction mixture was evaporated to dryness and redissolved in water/acetonitrile. Finally, the solution was lyophilized to obtain the product Compound 14 (285.0 mg, 98.6 %). LCMS (ESI-MS): calculated m/z 566.22 $[M+H]^+$; found 566.47 $[M+H]^+$. ^1H NMR (400 MHz, $CDCl_3$): δ 7.92-7.42 (8H, m, ArH), 7.15 (4H, m, ArH), 6.29 (2H, q, −CH−), 4.50 (2H, m, $-CH_2-$), 4.32 (2H, m, $-CH_2-$), 3.32 (4H, t, $-CH_2-$), 2.82 (2H, t, $-CH_2-$), 1.25 (6H, d, $-CH_3$). ^1H NMR spectra of PC_2XYL-NH_2 have been shown in Figure S43.

2.6.3 Synthesis of Compound 15: Compound 14 (20.0 mg, 0.035 mmol) was dissolved in 2.5 ml of dry acetonitrile in a 5 ml pear-shaped flask under nitrogen inert atmosphere. Triethylamine (TEA, 14.8 µl, 0.106 mmol) and Bis(NHS)PEG_5 (94.1 mg, 0.177 mmol) were added sequentially and stirred at room temperature under an inert atmosphere. After 1 h the reaction mixture was immediately evaporated to dryness using rotary evaporator, diluted with 1 ml mixture of ACN-water, and purified using reverse-phase flash column chromatography [Solvents: 0.1% Trifluoroacetic acid (TFA), 5% acetonitrile, water (A), 0.1% Trifluoroacetic acid (TFA), acetonitrile (B); gradient: 5 to 100% of B in 35 min]. After the column, the product Compound 15 was lyophilized to get a viscous compound (R_t = 19.0, 22.8 mg, Yield: 65.6 %). HRMS: (ESI-MS): calculated m/z 983.3880 $[M+H]^+$; found 983.3859 $[M+H]^+$. ^1H NMR (600 MHz, DMSO-d_6): δ 7.96-7.55 (8H, m, ArH), 7.24-7.03 (4H, m, ArH), 6.03-5.98 (2H, q, −CH−), 4.32 (2H, m, $-CH_2-$), 4.11 (2H, t, $-CH_2-$), 3.69-3.46 (20H, m, $-OCH_2CH_2O-$), 3.21-3.11 (4H, m, $-CH_2-$), 2.90 (2H, t, $-CH_2-$), 2.79 (4H, s, $-CH_2-$), 2.20 (2H, t, $-CH_2-$), 1.54 (6H, d, $-CH_3$), 1.36 (9H, s, $-CH_3$). ^1H NMR spectra of PC_2XYL-PEG-NHS has been shown in Figure S44.

3. Preparation of targeting conjugates

3.1 Conjugation of ADA with the antibody

1. Secondary antibodies (Donkey anti-Rat and Donkey anti-human) were buffer exchanged by Zeba^{TM} spin desalting column 7K MWCO, where columns were pre-equilibrated with PBS containing 10 % 1M $NaHCO_3$.
2. Buffer exchanged secondary antibody (50 µg, 3.33x10^{-4} µmol) was first taken in a 1.5 ml microcentrifuge tube.
3. Nitrobenzyl-protected ADAPc-PEG-NHS (Compound 12) (3.32 µg, 3.33x10^{-3} µmol, 4.5 µl solution from of 0.75 mg ml^{-1} in DMF) were added in two portions (2.25 µl each time) to the buffer exchanged secondary antibody solution.
4. The reaction was kept for gentle shaking at 4°C for 12 h.
5. After that ADAPc-PEG conjugated antibody was purified by Zeba^{TM} spin desalting column 7K MWCO, where columns were pre-equilibrated with PBS.
6. The purified antibody was then subjected to irradiation using 365 nm UV light (50 mW.cm^{-2}) for 5 min to generate ADA conjugated antibody.
7. The ADA conjugated antibody was then characterized by MALDI-MS (Figure S6 and S32) and proceeded with the fluorogenic microscopy experiment.

3.2 Conjugation of XYL with antibody

1. Donkey anti-Rat secondary antibody was buffer exchanged by Zeba^{TM} spin desalting column 7K MWCO, where columns were pre-equilibrated with PBS containing 10 % 1M $NaHCO_3$.
2. Buffer exchanged secondary antibody (44.66 µg, 2.98 x10^{-4} µmol) was first taken in a 1.5 ml microcentrifuge tube.
3. A 0.73 µl solution of nitrobenzyl-protected XYL-PEG-NHS (PC_2XYL-PEG-NHS, compound 15) (10 mg ml^{-1} in DMSO) was diluted to 3 µl by DMSO and added to the secondary antibody solution in two portions (1.5 µl each time).
4. The reaction was kept at 4°C overnight.
5. After that nitrobenzyl-protected XYL-PEG conjugated antibody was purified by Zeba^{TM} spin desalting column 7K MWCO, where columns were pre-equilibrated with PBS. The purified antibody was then subjected to irradiation using 365 nm UV light (50 mW.cm^{-2}) for 5 min to cleave the nirtobenzyl group to generate XYL conjugated antibody.

3.3 Conjugation of ADA with phalloidin

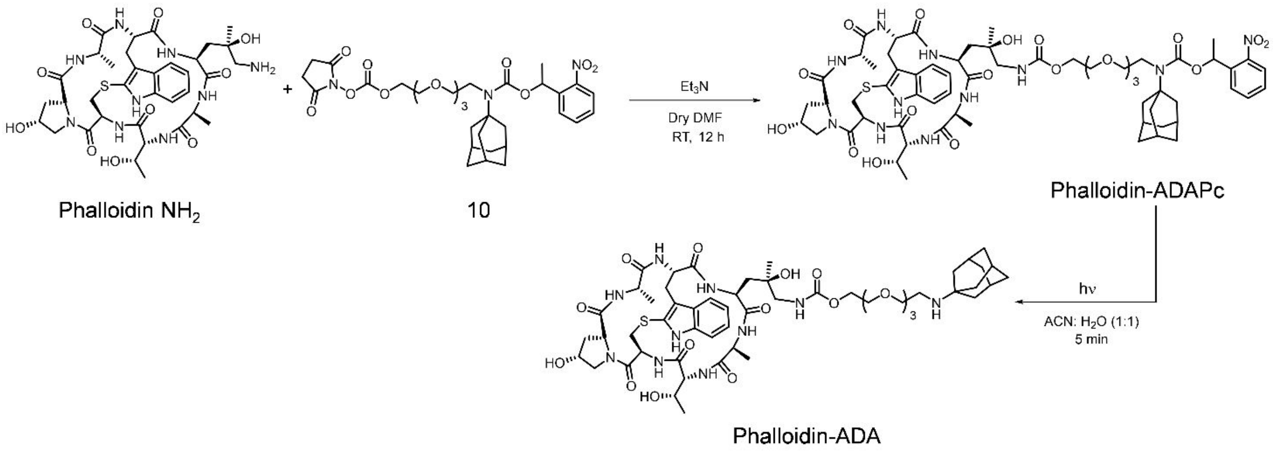

Scheme S6: Synthetic scheme for the preparation of ADA conjugated phalloidin**.**

1. Phalloidin–ADAPc was synthesized using previously reported protocols by our group[4].
2. The ADAPc conjugated phalloidin was then dissolved in a 1:1 mixture of water/acetonitrile and irradiated with a 365 nm UV lamp (50 mW) for 5 min to photocleave the Pc group followed by purified by HPLC using water/acetonitrile as eluent.
3. The ADA conjugated phalloidin was then characterized by HRMS and proceeded for the fluorogenic microscopy experiment. Yield: quantitative (from HPLC). HRMS (ESI-MS): Calculated m/z 1141.5598 $[M+H]^+$; found 1141.5598 $[M+H]^+$.

3.4 Conjugation of ADA with taxol

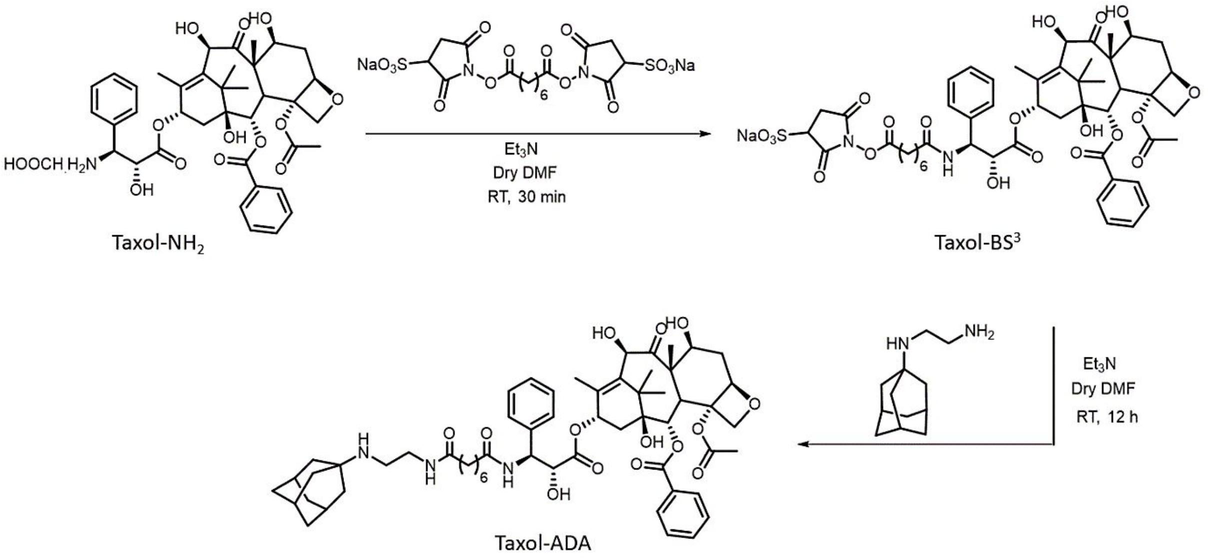

Scheme S7: Synthetic scheme for the preparation of ADA conjugated taxol**.**

1. Amine derivative of docetaxel (80 μg, 16 μl from 5 mg ml^{-1} stock solution in dry DMF, 0.1061 μmol) was taken in a 0.5 ml microcentrifuge tube.
2. 4.2 μl DMF solution containing 0.042 μl Et_3N was added to it.
3. Bis(sulfosuccinimidyl) suberate (BS^3) (1.5 mg, 2.84 μmol, dissolved in 30 μl DMF) was added to it and the reaction was stirred at room temperature for 30 min.
4. After that, the reaction mixture was diluted using 50 μl of Milli-Q water and injected into HPLC for purification using water/acetonitrile containing 0.1% TFA as eluent.
5. The taxol-BS3 conjugate was characterized by using LCMS. LCMS (ESI-MS): Calculated m/z 1041.35 $[M+H]^+$; found 1041.56 $[M+H]^+$.
6. The purified compound (Yield: quantitative from HPLC chromatogram) was then dissolved in dry DMF for conjugation with ADA.
7. In a separate microcentrifuge tube, compound 7 (0.187 mg, 1.016 μmol, dissolved in 20 μl DMF) was taken and triethylamine (2.82 μl DMF solution containing 0.141 μl Et_3N) was added to it.
8. Next, the solutions were mixed together and allowed to stir at room temperature for 12 h.
9. After the completion of the reactions, the reaction mixture was diluted using 50 μl of Milli-Q water and injected into HPLC for purification using water/acetonitrile containing 0.1% TFA as eluent.
10. The ADA conjugated taxol was then characterized by LCMS and ^1H NMR. LCMS (ESI-MS): Calculated m/z 1040.55 $[M+H]^+$; found 1040.73 $[M+H]^+$.
   ^1H NMR (DMSO–d^6, 600 MHz) δ 8.36 (d, J = 8.0 Hz, 1H), 8.00 (t, J = 7.8 Hz, 2H), 7.84 (d, J = 8.4 Hz, 1H), 7.71 – 7.59 (m, 2H), 7.48 – 7.18 (m, 5H), 5.95 – 5.88 (m, 2H), 5.43 (d, J = 7.2 Hz, 1H), 5.28 (dd, J = 6.0, 3.0Hz, 1H), 5.17 – 4.90 (m, 4H), 4.59 (s, 1H), 4.42 (t, J = 6.6 Hz, 1H), 4.12 – 3.98 (m, 3H), 3.76 (q, J = 6.6 Hz, 1H), 2.99 (s, 1H), 2.93 – 2.87 (m, 2H), 2.29 (s, 1H), 2.24 (s, 3H), 2.20 – 2.15 (m, 2H), 2.12 (br, 3H), 2.09 – 1.80 (m, 6H), 1.79 – 1.74 (m, 8H), 1.69 – 1.58 (m, 6H), 1.53 (s, 3H), 1.46 (q, J = 6.6 Hz, 4H), 1.21 – 1.09 (m, 4H), 1.06 – 0.99 (m, 6H). The HPLC and ^1H–NMR of ADA-taxol conjugate have been shown in Figure S29 and S45, respectively.

3.5 Conjugation of TCO with phalloidin

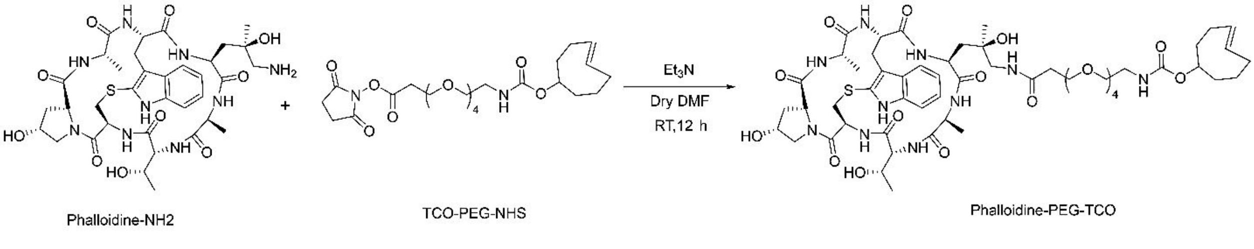

Scheme S8: Synthetic scheme for the preparation of TCO conjugated phalloidin.

1. Phalloidin amine (10 mg, 1 ml from 10 mg. ml^{-1} stock in DMSO, 0.0127 mmol) was taken in a microcentrifuge tube and diluted to 10 ml using dry DMSO.
2. Triethylamine (6.09 mg, 8.5 ml from 1:1000 (v/v) stock in DMSO, 0.6029 mmol) was added.
3. TCO-PEG-NHS ester (31.2 mg, 3.12ml from 10 mg. ml^{-1} stock in DMSO, 0.6029 mmol) was added to the reaction mixture and stirred at room temperature for 12 h. This TCO-PEG-NHS was procured from Click Chemistry Tools (Catalog#A137-25).
4. After the reaction, the reaction mixture was diluted to a 1:1 water/DMSO mixture and directly injected into HPLC for purification (Figure S30).
5. The purified product was dried under vacuum and characterized by LCMS. The yield calculated is ~80% (from HPLC). LCMS (ESI–MS): calculated 1187.56 [M+H]$^+$, 1209.54 [M+Na]$^+$; found 1187.81 [M+H]$^+$, 1209.78 [M+Na]$^+$.

3.6 Conjugation of TCO with jasplakinolide

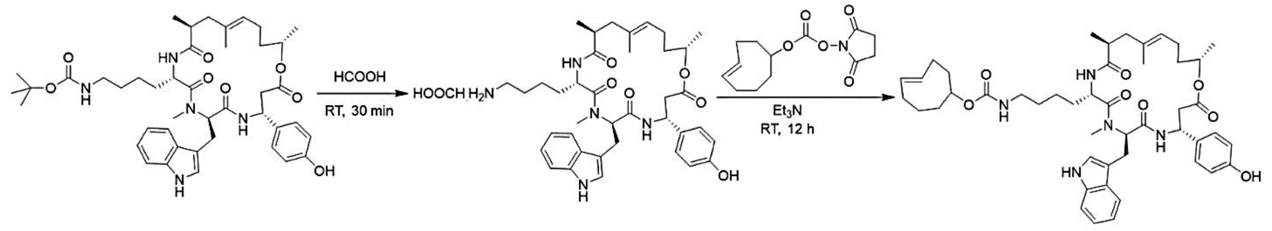

Scheme S9: Synthetic scheme for the preparation of TCO–jasplakinolide conjugate.

3.6.1 Jasplakinolide–lysine amine preparation

Boc protected lysine derivative of jasplakinolide (100 mg, 0.1291 mmol) was dissolved in 100 ml formic acid and incubated for 30 min at room temperature. The formic acid solution was diluted using water and dried under a vacuum for 12 h to remove the acid traces completely. The crude jasplakinolide lysine derivative (considering quantitative yield) was dissolved in dry DMSO and characterized by LCMS. LCMS (ESI–MS): calculated 674.39 [M+H]$^+$; found 674.57 [M+H]$^+$.

3.6.2 Jasplakinolide–TCO conjugate

1. Jasplakinolide–lysine amine (10 mg, 1 ml from 10 mg.ml^{-1} stock in DMSO, 0.015 mmol) was taken in a microcentrifuge tube and diluted to 10 ml using dry DMSO.
2. Triethylamine (4.54 mg, 6.3 ml from 0.1% (v/v) stock in DMSO, 0.045 mmol) was added.
3. TCO NHS ester (61.5 mg, 6.15 ml from 10 mg. ml^{-1} stock in DMSO, 0.23 mmol) was added into the reaction mixture and stirred at room temperature for 12 h. This TCO-PEG-NHS was procured from Click Chemistry Tools (Catalog# 1016-25).
4. After the reaction, the reaction mixture was diluted to a 1:1 water/DMSO mixture and directly injected into HPLC for purification (Figure S31).

5. The purified product was dried under vacuum and characterized by HRMS. The yield calculated is ~70% (from HPLC). HRMS (ESI–MS): calculated 826.4749 $[M+H]^+$, 848.4569 $[M+Na]^+$; found 826.4676 $[M+H]^+$, 848.4496 $[M+Na]^+$.

4. Synthetic of XYL decorated gold nanoparticle (XYL–AuNP)

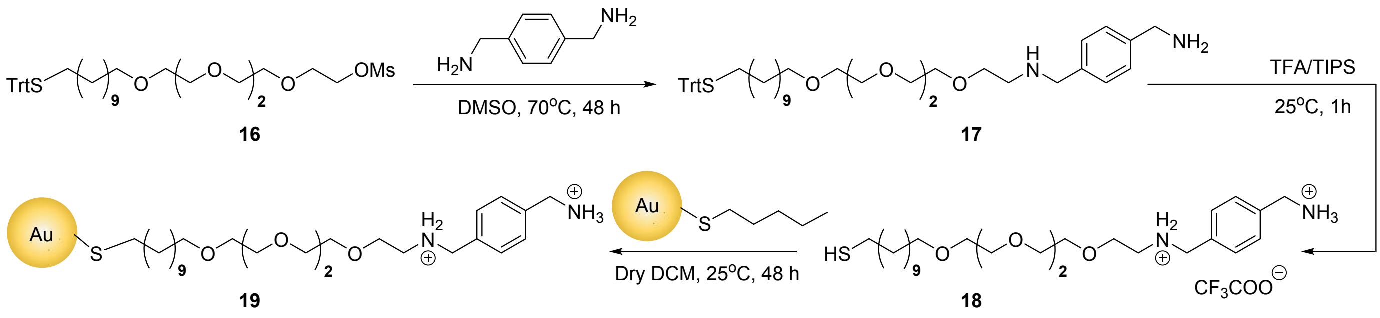

Scheme S10: Synthetic scheme for the preparation of XYL–AuNP.

4.1. Synthesis of Compound 17 (Trt-C11-TEG-XYL)

In a 100 ml round-bottom flask xylene diamine (1.11 g, 8.16 mmol) was taken and dissolved with 5 ml of dry DMSO. In a separate 100 ml RB, Trt-C11-TEG-XYL[5] (compound 16, 280 mg, 0.39 mmol) was dissolved in 2 ml of dry DMSO. Next, the DMSO solution of Compound 1 was added to the xylene diamine solution (in DMSO) slowly in a dropwise manner. The reaction mixture was stirred at 70°C for 48 h. After that, the reaction mixture was dissolved in DCM and washed with water (30 ml × 2), and brine (30 ml × 2), dried over Na_2SO_4 and concentrated under reduced pressure. Next, the reaction mixture was purified using reverse-phase flash column chromatography (C-18 20 g column used) with water/acetonitrile as eluent. The purified product was characterized by LCMS and dried by lyophilization. Compound 17 was thus obtained as a yellowish-brown oil (265 mg, Yield 89.5 %). ^{1}H–NMR (600 MHz, DMSO-d^6): δ 8.32 (s, 2H), 7.51 (q, 4H) 7.35 – 7.23 (m, 15H), 4.19 (s, 2H), 4.06 (s, 2H), 3.55 (s, 2H), 3.33 – 3.50 (m, 16H), 2.06 (t, 2H), 1.45 (m, 2H), 1.06-1.28 (m, 18H). LCMS (ESI-MS): calculated 741.56 $[M+H]^+$; obtained 741.72 $[M+H]^+$. The ^{1}H–NMR of Compound 17 has been shown in Figure S46.

4.2. Synthesis of Compound 18 (HS-C11-TEG-XYL)

Compound 17 (100 mg, 0.13 mmol) was taken with trifluoroacetic acid (TFA, 2.6 mmol) and triisopropylsilane (TIPS, 0.39 mmol) in dry DCM (3ml). During the addition of TFA, the solution slowly turned yellow, which after the addition of TIPS resulted in a colorless solution. The reaction mixture was stirred at room temperature for 5 h under an N_2 atmosphere. After the completion of the reaction,

the volatile components (solvent, TFA, TIPS) were removed under reduced pressure and the obtained residue was washed with hexane (4 times). The compound 18 obtained was dried (64.0 mg, yield 65.7 %) and characterized by ^{1}H NMR. ^{1}H NMR (400 MHz, DMSO-d^6): δ 9.03 (s, 2H), 8.27 (s, 2H), 7.55 – 7.49 (m, 4H), 4.20 (s, 2H), 4.07 (s, 2H), 3.69 (t, *J* = 5.2 Hz, 2H), 3.57 – 3.34 (m, 14H), 3.08 (d, *J* = 7.2 Hz, 2H), 1.61 – 1.34 (m, 4H), 1.25 – 1.21 (s, 14H). HRMS (ESI-MS): calculated 499.3564 $[M+H]^+$; obtained 499.4186 $[M+H]^+$. The ^{1}H–NMR of Compound 18 has been shown in Figure S47.

4.3. Surface functionalization of AuNP (Compound 19)

To fabricate AuNPs, we followed a two-step procedure. Firstly, we synthesized 1-Pentanethiol-coated gold nanoparticles (d~2 nm) using the literature procedure.[6] Xylene functionalized gold nanoparticles (XYL–AuNP) were prepared by place exchange of pentanethiol capped ~2 nm gold nanoparticles (Au-C_5).

In a 20 ml vial Au-C_5 (3.2 mg) was dissolved in nitrogen-purged dry DCM (1 ml). In another vial xylene diamine thiol ligand (compound 18, 15.6 mg) was dissolved in nitrogen-purged dry DCM (1 ml) plus methanol (0.1 ml) and transferred to the first vial. The reaction was stirred at RT for 48 h under an N_2 atmosphere. The solvent was evaporated under reduced pressure and the nanoparticles were washed 3 times with hexane. Nanoparticles were recovered by centrifugation and the supernatant was discarded. After that, the nanoparticles were redispersed in water and then dialyzed for 48 h using Snake-Skin Dialysis Membrane 10K MWCO to get the surface functionalized XYL-AuNP. Then, the surface functionalized XYL-AuNP was characterized by MALDI (Figure S51). The concentration of the functionalized AuNPs was measured to be 9.13 μM. The MALDI spectrum showed a peak at m/z = 499.072 (observed) which corresponds to the calculated mass value (m/z = 499.356) of the thiol ligand of xylene diamine moiety.

5. Synthetic protocol for Tre-ADA

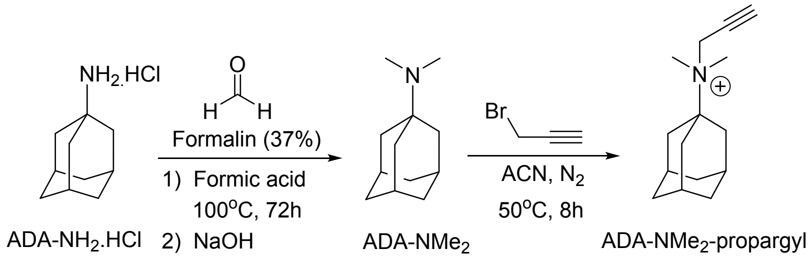

Scheme S11: Synthetic scheme for the preparation of ADA-NMe_2-propargyl.

5.1 Synthesis of Compound ADA-NMe_2

In a 100 ml round-bottom flask, adamantane hydrochloride (2.0 g, 10.65 mmol) was dissolved in 1:1 (v/v) of formic acid (10 ml) and formalin (10 ml). The reaction mixture was refluxed at 100°C for 72 h. Upon completion of the reaction, the reaction mixture was kept at room temperature for 1 h. Next, the reaction mixture was neutralized with ~75 ml of 20% (w/w) NaOH solution (pH~ 14). A colorless oily layer was formed, which was extracted with toluene (sulfur-free). The organic solvent was evaporated, and ADA-NMe_2 was obtained as a colorless oil (1.0 g, Yield 48%). HRMS (ESI-MS): Calculated m/z 180.1746 $[M + H]^+$, Found 180.1749 $[M + H]^+$. ^{1}H-NMR ($CDCl_3$, 400 MHz), δ (ppm): 2.27 (s, 6H), 2.07 (s, 3H), 1.69-1.57 (12H, m, –CH2). ^{1}H–NMR of ADA-NMe_2 has been shown in Figures S48.

5.2 Synthesis of Compound ADA-NMe_2-propargyl

In a 50 ml round-bottom flask, N, N-dimethyl adamantane (ADA-NMe_2, 0.5 g, 2.78 mmol) was taken and 10 mL of dry acetonitrile was added. The reaction mixture was stirred for 10 min at room temperature. Next, propargyl-bromide (1.65 g, 13.87 mmol) was added to the reaction mixture. The solution was stirred at 50°C for 8 h. Upon completion of the reaction, the reaction mixture was kept for 30 min at room temperature. The resulting precipitate i.e. formed during the reaction was filtered and washed two times with acetonitrile and diethyl ether (4:3 v/v) to obtain a white powdery solid as ADA-NMe_2-propargyl (340.0 mg, Yield 56%). HRMS (ESI-MS): Calculated m/z 218.1903 $[M]^+$, Found 218.1897 $[M]^+$. ^{1}H-NMR (DMSO-d_6, 400 MHz), δ (ppm): 4.32 (s, 2H), 4.02 (s, 1H), 2.96 (s, 6H), 2.22 (s, 3H), 2.05 (s, 6H), 1.62 (s, 6H). ^{1}H–NMR of ADA-NMe_2-propargyl has been shown in Figures S49.

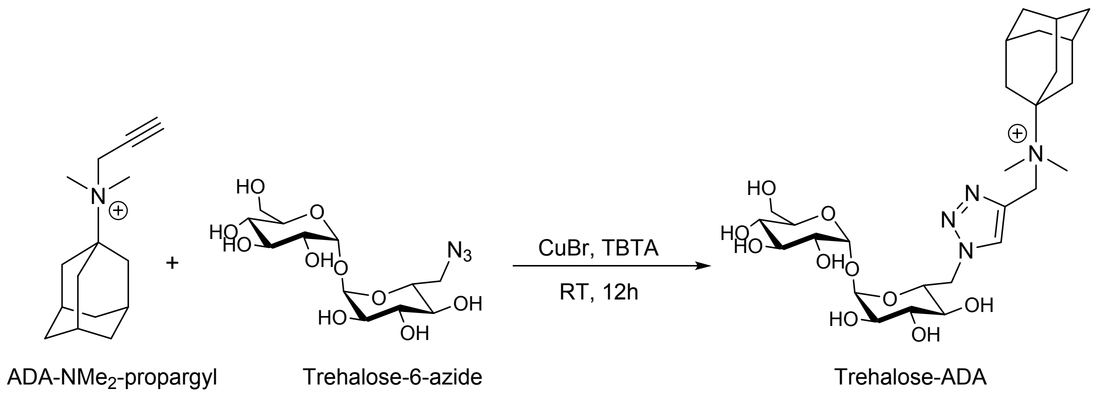

Scheme S12: Synthetic scheme for the preparation of Trehalose-ADA.

5.3 Synthesis of Trehalose-ADA

In a 0.5 ml microcentrifuge tube, Trehalose-6-azide (1 mg, 100 µl from 10 $mg.ml^{-1}$ stock in dry DMSO, 0.0027 mmol) was taken and N, N-dimethyl adamantane propargyl (ADA-NMe_2-propargyl, 0.458 mg, 45.8 µl from 10 $mg.ml^{-1}$ stock in dry DMSO, 0.0021 mmol) was added. Next, CuBr (1.93 mg, 33.17 µL

from 400 mM stock in dry DMSO) and TBTA (7.163 mg, 33.73 µL from 400 mM stock in dry DMSO) were added to the reaction mixture and stirred at room temperature for 12 h. The reaction mixture was diluted using water and injected into HPLC for purification. The purified product (750 µg, yield 47.4%) was characterized by HRMS and ^{1}H-NMR. HRMS (ESI-MS): Calculated m/z 585.3130 $[M]^+$, Found 585.3122 $[M]^+$ ^{1}H-NMR (DMSO-d_6, 600 MHz): δ (ppm): 8.38 (s, 1H), 5.36 (d, 1H), 5.00 (d, 1H), 4.86-4.70 (m, 6H), 4.52 (m, 3H), 4.29 (t,1H), 4.11 (t, 1H), 3.60-3.42 (m, 6H), 3.12-3.01 (m, 5H), 2.76 (d, 6H), 2.27 (s, 3H), 2.16 (s, 6H), 1.67 (m, 6H). ^{1}H–NMR of Trehalose-ADA has been shown in Figure S50.

6. Fluorescence titration and MALDI-MS experiments

6.1 Protocol for fluorescence quenching study of CB7–FLs with XYL–Qs

The fluorescence quenching titrations of CB7–FLs vs XYL–Qs were performed using a fluorescence microplate reader. In this experiment, CB7–FLs were taken in a black 96 well microplate (Corning, non–binding plate) with a final concentration of 1 mM in 200 µl PBS buffer. Fluorescence spectra were recorded at respective excitation and emission wavelength scans listed in the table below (Table S5a). After this, 0.25 eq of XYL–Qs (final concentration 0.25 mM in 200 µl) were added to the system followed by the recording of fluorescence spectra. The addition of XYL–Qs (0.25 eq each) was subsequently continued followed by spectra recording for the respective CB7–FLs to achieve maximum quenching.

Similar protocols have been followed for quenching studies of CB7–FL against EtA–Q.

6.2 Protocol for fluorogenic response study from CB7–XYL quenched complex.

The CB7–FLs were taken in a black 96 well microplate (Corning, non–binding plate) with a final concentration of 1 mM in 200 µl PBS buffer. The respective equivalent of XYL–Qs were added to each well of CB7–FLs to achieve maximum quenching of fluorophores (Table S6).

To achieve stepwise recovery (Fig. 2b), ADA–NH_2 (final concentration 0.25 µM, 0.25 eq) was added to the quenched [CB7–FL·XYL–Q] systems, and fluorescence recovery kinetics were recorded every 30 seconds for 10 minutes. After 10 min, another 0.25 eq ADA–NH_2 was added, and recovery kinetics was again recorded. This protocol was followed for 4 successive additions of ADA (total 1 eq, final concentration 1 µM). The excitation and emission wavelengths that are used for kinetics measurements are listed in Table S5b. The exact concentrations of CB7-FL and XYL-Q that were used for Fig. 2b is given below:

CB7-Coumarin: [CB7-Coumarin (1 µM) + XYL-Dabcyl (1.5 µM)]; CB7-Fluorescein: [CB7-Fluorescein (1 µM) + XYL-BHQ1 (1 µM)]; CB7-Bodipy: [CB7-Bodipy (1 µM) + XYL-BHQ1 (1 µM)]; CB7-Cy3: [CB7-Cy3 (1 µM) + XYL-BHQ2 (2 µM)]; CB7-TAMRA: [CB7-TAMRA (1 µM) + XYL-BHQ2 (1.5 µM)]; CB7-Cy5: [CB7-Cy5 (1 µM) + XYL-BHQ3 (1.5 µM)]; CB7-SiR: [CB7-SiR (1 µM) + XYL-BHQ3 (2.5 µM)].

Other studies with quenched complex and recovery: the specific experimental conditions for preparing the quenched complex and conducting the recovery studies are provided in the corresponding figure descriptions.

Table S5a: Table for excitation and emission wavelength of CB7–FL used in fluorogenic response experiment.

CB7–FL	XYL–Q/EtA–Q	Excitation wavelength (l_{ex})	Emission range (l_{em})
Coumarin	Dabcyl	350 nm	(425-525) nm
Fluorescein	BHQ1	465 nm	(500-600) nm
Bodipy	BHQ1	465 nm	(500-600) nm
Cy3	BHQ2	520 nm	(550-650) nm
TAMRA	BHQ2	520 nm	(550-650) nm
Cy5	BHQ3	620 nm	(650-750) nm
SiR	BHQ3	620 nm	(650-750) nm

Table S5b: Table for excitation and emission wavelength of CB7–FL used in fluorescence recovery experiment.

CB7–FL	XYL–Q	Excitation wavelength (l_{ex})	Emission wavelength (l_{em})
Coumarin	Dabcyl	350 nm	450 nm
Fluorescein	BHQ1	465 nm	520 nm
Bodipy	BHQ1	465 nm	515 nm
Cy3	BHQ2	510 nm	565 nm
TAMRA	BHQ2	510 nm	585 nm
Cy5	BHQ3	620 nm	665 nm
SiR	BHQ3	620 nm	675 nm

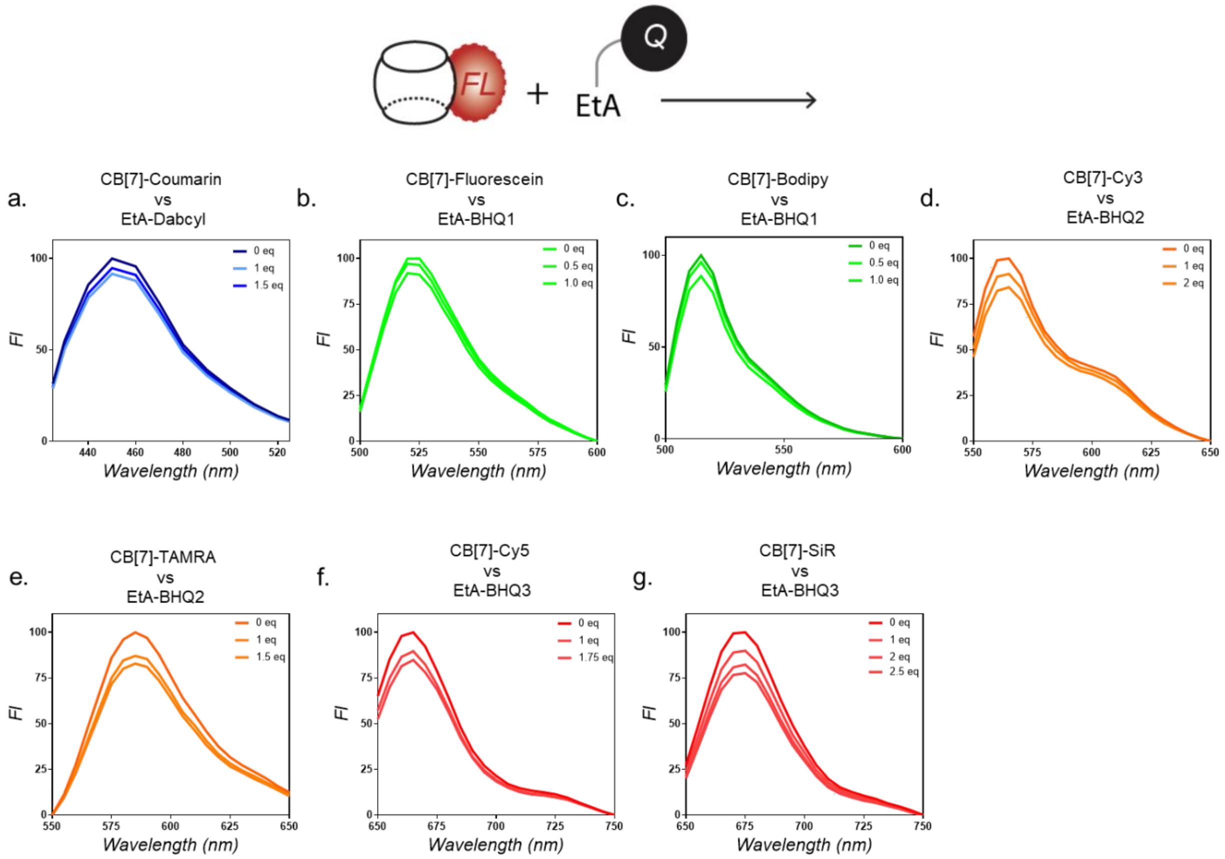

Figure S1: Fluorescence titration data for CB7–FL vs Ethanolamine conjugated quencher (EtA–Q). Negligible fluorescence quenching from the non–interacting pair demonstrated that the fluorescence quenching requires specific recognition of CB7 host and XYL guest moiety.

Table S6. The concentration of XYL-Q quencher used with respect to CB7-FL

SL No	CB7-FL	XYL-Q
1	CB7-Coumarin (1.0 μM)	XYL-Dabcyl (1.5-2 μM)
2	CB7-Fluorescein (1.0 μM)	XYL-BHQ1 (1.0-1.5 μM)
3	CB7-Bodipy (1.0 μM)	XYL-BHQ1 (1.0-1.5 μM)
4	CB7-Cy3 (1.0 μM)	XYL-BHQ2 (2.0-2.5 μM)
5	CB7-TAMRA (1.0 μM)	XYL-BHQ2 (1.5-2 μM)
6	CB7-Cy5 (1.0 μM)	XYL-BHQ3 (1.5-2.5 μM)
7	CB7-SiR (1.0 μM)	XYL-BHQ3 (2.5-3 μM)

6.3 Protocol for MALDI-MS analysis.

I. The CB7-FL (FL = Coumarin, TAMRA, SiR, and Cy5) was taken in 1 µM concentration for the study. Xyl-Q was taken in concentration with respect to the type of quencher [XYL-Q, Q = Dabcyl (1 µM), BHQ2 (2 µM), BHQ3 (3 µM)]. The respective quencher and fluorophore were combined in appropriate concentrations in Milli-Q water [CB-Cy5 (1 µM) + XYL-BHQ3 (3 µM), CB-SiR (1 µM) + XYL-BHQ3 (3 µM), CB-TAMRA (1 µM) + XYL-BHQ2 (2 µM), CB-Coumarin (1 µM) + XYL-Dabcyl (1 µM)]. The resulting solution was incubated for 30 min at RT. Next, the host-guest complex solution (2 µL) was combined with 2 µL of matrix solution of α-cyano-4-hydroxycinnamic acid (solvent – 70:30 Acetonitrile/Water) for MALDI-MS analysis. The matrix mixed solution was deposited on a MALDI plate for analysis.

II. The displacement of the XYL-Q quencher from the [CB-FL·XYL-Q] complex (prepared using the above-described protocol) was analyzed by adding 1-Adamantylamine hydrochloride (ADA) to the solution. The concentration of ADA used was the same as that of the respective quencher. The resulting solution was incubated for 30 minutes before performing MALDI-MS analysis (using the same matrix as in protocol I).

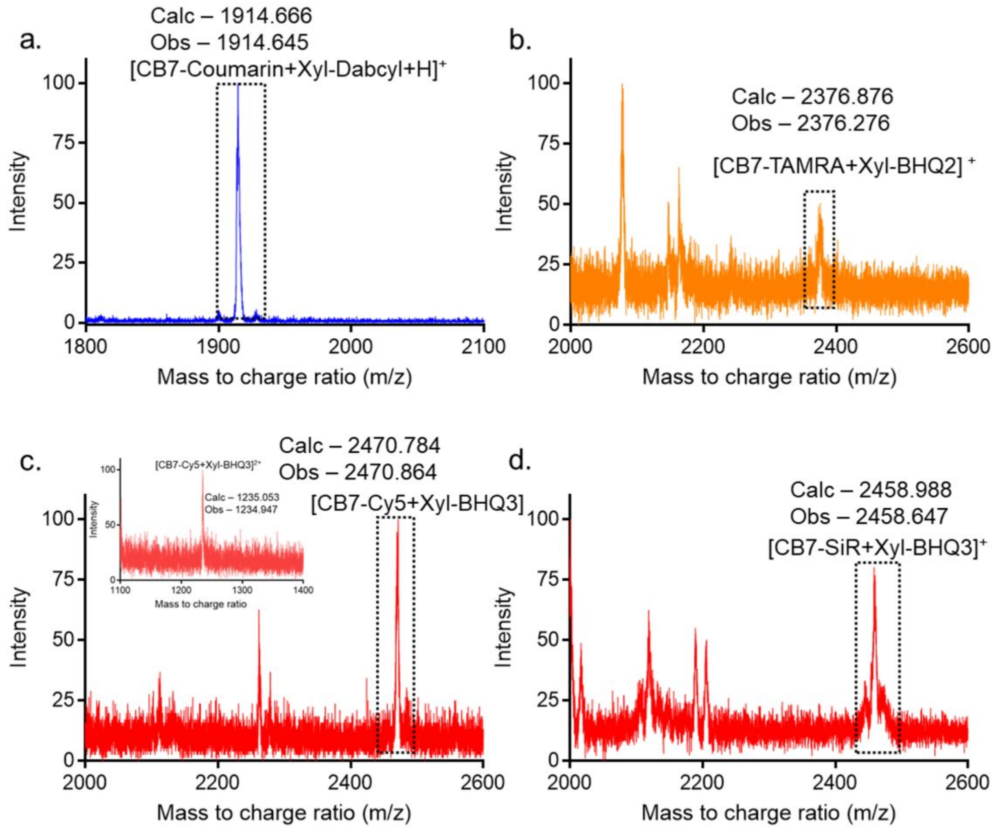

Figure S2: MALDI-MS spectroscopic data showing the detection of CB7-FL·XYL-Q monovalent complex. a) CB7-Coumarin·XYL-Dabcyl b) CB7-TAMRA·XYL-BHQ2 c) CB7-Cy5·XYL-BHQ3 d) CB7-SiR·XYL-BHQ3.

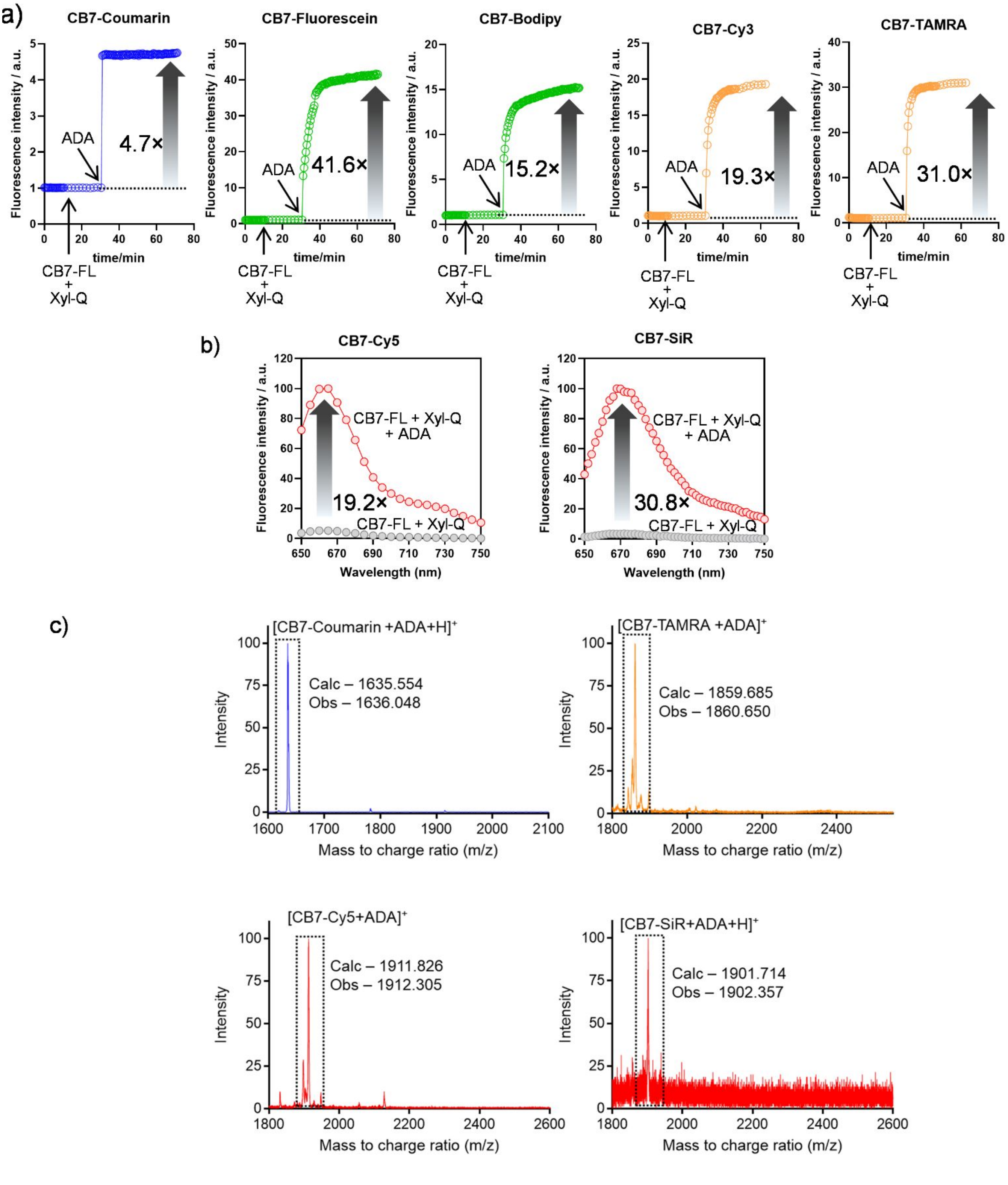

Figure S3: Fluorescence enhancement and MALDI-MS analysis of ADA mediated exchange reaction (CB7-FL·XYL-Q + ADA). a) Fluorescence enhancement kinetics and turn-on ratio for CB7-FL·XYL-Q pairs upon addition of ADA. CB7-Coumarin: [CB7-Coumarin (1 μM) + XYL-Dabcyl (2 μM)] + ADA (8 μM); λ_{ex}=350 nm and λ_{em}=450 nm. CB7-Fluorescein: [CB7-Fluorescein (1 μM) + XYL-BHQ1 (1.5 μM)] + ADA (6 μM); λ_{ex}= 465 nm and λ_{em}=520 nm. CB7-Bodipy: [CB7-Bodipy (1 μM) + XYL-BHQ1 (1.5 μM)] + ADA

(6 μM); λ_{ex}= 465 nm and λ_{em}= 515 nm. CB7-Cy3: [CB7-Cy3 (1 μM) + XYL-BHQ2 (2.5 μM)] + ADA (10 μM); λ_{ex}= 510 nm and λ_{em}= 565 nm. CB7-TAMRA: [CB7-TAMRA (1 μM) + XYL-BHQ2 (2 μM)] + ADA (8 μM); λ_{ex}= 510 nm λ_{em}= 585 nm. b) Fluorescence turn-on ratio for CB7-Cy5·XYL-BHQ3 and CB7-SiR·XYL-BHQ3 complexes. CB7-Cy5: [CB7-Cy5 (1 μM) + XYL-BHQ3 (2.5 μM)] + ADA (7.5 μM); λ_{ex}= 620 nm. CB7-SiR: [CB7-SiR (1 μM) + XYL-BHQ3 (2.5 μM)] + ADA (5 μM); λ_{ex}= 620 nm. c) MALDI-MS based analysis of ADA mediated exchange reaction for [CB7-Coumarin·XYL-Dabcyl] + ADA, [CB7-TAMRA·XYL-BHQ2] + ADA, [CB7-Cy5·XYL-BHQ3] + ADA, and [CB7-SiR·XYL-BHQ3] + ADA. The complete disappearance of CB7-FL·XYL-Q complex mass and observation of CB7-FL·ADA complex mass indicated a quantitative displacement reaction.

III. protocol for MALDI-MS characterization of ADA-conjugated antibodies:

Antibodies (1 μL) after desalting through Zeba spin column (~ 1 mg.mL^{-1} in Milli-Q water) were taken in a microcentrifuge tube containing 1 μL of sinapinic acid [10 mg.mL^{-1} in 50:50 water (0.1% TFA):acetonitrile)]. The mixed solution was placed onto a MALDI plate and allowed to dry at room temperature for analysis.

6.4 Protocol for fluorescence quenching study of CB7–TAMRA with XYL–AuNP

The fluorescence quenching titrations of CB7–TAMRA vs XYL–AuNP were performed using a fluorescence microplate reader. In this experiment, CB7–TAMRA was taken in a black 96-well microplate (Corning, non–binding plate) with a final concentration of 1 μM in 200 μl PBS buffer. Fluorescence spectra were recorded at respective excitation and emission wavelength scans listed in the table (Table S5a). After this, 0.005 eq of XYL– AuNP (final concentration 5.0 nM in 200 μl) was added to the system followed by the recording of fluorescence spectra. The addition of XYL–AuNP (0.005 eq in the first four steps, followed by 0.01 eq in the subsequent three steps) was continued, with spectra recorded for the respective CB7–TAMRA to achieve maximum quenching. In the final two steps, the equivalent of XYL–AuNP was increased to reach final concentrations of 0.1 μM and 0.2 μM, respectively.

Similar protocols have been followed for quenching studies of TAMRA-EtA against XYL–AuNP (Figure S17a).

6.5 Protocol for fluorogenic response study from CB7–TAMRA·XYL–AuNP quenched complex.

The CB7–TAMRA was taken in a black 96 well microplate (Corning, non–binding plate) with a final concentration of 1 μM in 200 μl PBS buffer. About 0.2 equivalent of XYL– AuNP was added to CB7–TAMRA to achieve maximum quenching of the fluorophore. After this, ADA–NH_2 (final concentration 20.0 μM, 20.0 eq) was added to the quenched [CB7–TAMRA·XYL–AuNP] systems, and fluorescence

recovery was recorded after 10 minutes. The excitation and emission wavelengths used are listed in Table S5a.

7. Labeling experiment with *in vitro* polymerized microtubule

7.1 *In vitro* preparation and labeling of microtubules (MTs)

The *in vitro* synthesized microtubules were inherently labeled with Alexa568 and biotin to examine the targeting specificity of the host-guest probe.

Labeling reaction

A warm Ti90 tube was filled with a 2.5 ml prewarmed high pH cushion. A polymerized MT mix was layered on top of the high pH cushion and spun at 43k rpm for 40 min at 35°C. The supernatant was then aspirated, and the pellet was washed with warm labeling buffer. The pellet was then resuspended in 600 µl of warm labeling buffer (containing Guanosine triphosphate (GTP)). After subsequent washings, a total volume of 1 ml was transferred to a 2 ml centrifuge tube for labeling reaction. 20 µl of dye-NHS (Alexa568-NHS) was added to the resuspended MT mix and kept in a 37°C water bath. Mixed thoroughly by inversion. Vortexed gently and intermittently every 5 min for 20 min. Added remaining dye-NHS and repeated the previous process. The reaction mix was then layered on 200 µl of prewarmed low pH cushion (containing GTP) in TLA 120.1 tubes and spun at 86k rpm for 20 min at 35°C. Pellet was washed with warm labeling buffer.

De-polymerization and polymerization cycle

The pellet was resuspended in 100 µl of ice cold depolymerization buffer and placed on ice for 20 min. Spun at 80k rpm for 10 min at 4°C. To the supernatant, 5× BRB80, 2 mM $MgCl_2$, and 1mM GTP were added. It was then placed on ice for 5min. The polymerization was initiated by the addition of 350 µl prewarmed glycerol and it was allowed to polymerize for 30 min at 37°C. The polymerization mix was layered on 200 µl of prewarmed low pH cushion (with GTP) and spun at 80k rpm for 20 min at 35°C. Pellet was washed with warm BRB80 buffer. It was then resuspended in ice-cold BRB80 buffer. This was transferred to pre-cooled tubes and spun at 80k rpm for 10 min at 4°C. The supernatant was subsequently transferred to ice. The concentration of labeled tubulin was measured at 280 nm and aliquots were made. They were then snap-frozen and stored at -80°C until further use.

Buffers used:

1. BRB80 buffer

K- PIPES (pH – 6.8) – 80mM, $MgCl_2$ – 2mM and EGTA – 1mM

2. 5x BRB80 buffer

K- PIPES (pH – 6.8) – 400mM, $MgCl_2$ – 10mM and EGTA – 5mM

3. High pH cushion

HEPES (pH – 8.6) – 100mM, $MgCl_2$ – 1mM, EGTA – 1mM, glycerol – 60%

4. Low pH cushion

5x BRB80 (pH – 6.8) – 1mM, glycerol – 60%

5. Labeling buffer

HEPES (pH – 8.6) – 100mM, $MgCl_2$ – 1mM, EGTA – 1mM, glycerol – 40%

6. De-polymerization buffer

K-glutamate (pH – 7.0) – 100mM, $MgCl_2$ – 1mM

For the *in-vitro* synthesis of microtubules, 100 μM of tubulin heterodimers (α and β), 100 μM of labeled tubulin (Alexa568-NHS), 40 μM of biotin-conjugated tubulin, 1 mM of GMPCPP (nucleoside analog of GTP, with slower hydrolysis) were mixed in a microtubule polymerization buffer, BRB80 (80 mM PIPES buffer, 1 mM EGTA, 2 mM $MgCl_2$, pH–6.8). This mix was then placed in ice for 5 min to depolymerize the tubulin oligomers if any. It was then spun down at 14k rpm for 10 min at 4°C to pellet down the protein aggregates. The supernatant which contains the unpolymerized tubulin was then placed for polymerization at 37°C for two hours. This was followed by pelleting microtubules using a sucrose cushion which contains 50% sucrose in BRB80 buffer (spun down at 13k rpm for 10 min at 37°C to pellet down). The pellet was subsequently reconstituted in 1× BRB80 buffer and maintained at 37°C until further use.

7.2 Total internal reflection fluorescence (TIRF) imaging protocol *of in vitro* prepared MTs

For imaging of *in vitro*-microtubule labeling using CB7-ADA probe, a flow chamber was created using a clean microscopy slide, onto which two strips of double-sided adhesive tape is attached and a cover glass was placed on top. The flow cell was filled with 10 μl of BSA-Biotin (1 mg/ml) and incubated for 5 min, wherein BSA non-specifically interacts with the glass to facilitate immobilization of biotin on the surface. Washing was done to remove excess biotin by flowing in 1× BRB80 buffer twice through the chamber. Addition of 10 μl of streptavidin solution (0.5 mg/ml) for 5 min, which was then attached

firmly to biotin on the surface. Unbound streptavidin in the channel was washed twice with 1x BRB80 buffer with casein (1.25 mg/ml) which blocks unbound sites and averts non-specific binding. Then, 20µl of an appropriate dilution of the MTs (with incorporated biotin-tubulin) with 5 µM tag ADA-taxol in 1× BRB80-casein buffer was flown through the chamber. This complex was incubated in the chamber for 10 min. Excess was washed off using 1× BRB80- casein buffer. CB7-Bodipy was diluted in 1× BRB80-casein buffer to a concentration of 500 nM. It was then flown (20µl) through the channel and incubated for 10 min. The final step involves washing the flow chamber using an anti-bleach mix of PCA, PCD, and Trolox in 1× BRB80-casein buffer (40 µl). This was then imaged in the TIRF microscope under a 100× oil–immersion objective.

8. Protocol for fluorogenic labeling in cells, tissue and metabolically labeled bacteria

8.1 General cell culture protocol

1. MEF, BS-C-1, U2OS, A431, 3T3 and HeLa cells were used for the experimental study.
2. MEF, A431, 3T3, and HeLa cells were cultured in a humidified atmosphere (5% CO_2) at 37°C and grown in Dulbecco's Modified Eagle's Medium (DMEM, high glucose) supplemented with 10% fetal bovine serum (FBS) (Gibco, USA), 2 mM Glutamax (Invitrogen, USA) and 1% antibiotics (100 U/ml penicillin, 100 µg/ml streptomycin and 0.25 µg/ml amphotericin) (Gibco, USA).
3. U2OS cells cultured in humidified atmosphere (5% CO_2) at 37°C and grown in McCoy's 5A (Modified) Medium containing (+) L-Glutamine, supplemented with 10% fetal bovine serum (FBS) (Gibco, USA), 2 mM Glutamax (Invitrogen, USA) and 1% antibiotics (100 U/ml penicillin, 100 µg/ml streptomycin and 0.25 µg/ml amphotericin) (Gibco, USA).
4. BS-C-1 cultured in humidified atmosphere (5% CO_2) at 37°C and grown in Minimum Essential Medium (MEM) containing (+) non-essential amino acids (NEAA) and no glutamine, which further supplemented with 10% fetal bovine serum (FBS) (Gibco, USA), 2 mM Glutamax (Invitrogen, USA) and 1% antibiotics (100 U/ml penicillin, 100 µg/ml streptomycin and 0.25 µg/ml amphotericin) (Gibco, USA), 1 mM Sodium Pyruvate (Gibco, USA).
5. At ~80% confluence, the cells were washed with DPBS (pH 7.3) (Gibco, USA), trypsinized, and suspended in a culture medium.
6. Cells were counted and then in a typical experiment, ~10,000 cells/well in 200µL media were seeded in 96 well glass bottom plate (Eppendorf) / 35 mm glass-bottom cell imaging dish.
7. The cells were then maintained again in a humidified atmosphere (37°C, 5% CO_2) for 24 h to reach ~60% confluence. Thereafter, cells were used for imaging experiments.

8.2 Cell fixation, immunostaining and fluorogenic imaging protocol

1. Culture media was removed from 96 well glass bottom plates and washed using 200 μL PBS two times.
2. Cells were fixed with chilled methanol for 7 minutes at –20°C followed by washing with PBS three times.
3. Blocking with 3% bovine serum albumin (BSA) in PBS at 24°C for 2 h.
4. Cells were incubated for 24 h at 4°C with primary antibody against microtubules (10 μg ml^{-1}) diluted in PBS containing 3% bovine serum albumin.
5. Excess antibody was removed by three times washing with PBS (with 5 min incubation each time).
6. Cells were incubated with ADA conjugated secondary antibodies (10 μg ml^{-1}) diluted in PBS containing 3% bovine serum albumin for 2 h.
7. Excess secondary antibody was removed by three times washing with PBS (with 5 min incubation each time).
8. [CB7–FL·XYL–Q] quenched probes (CB7-FL concentration: 1 mM, XYL–Q concentration was varied according to the Table S6) were incubated with the ADA labeled cells and immediately proceeded for guest exchange based fluorogenic imaging.

8.3 Microtubule imaging with XYL-conjugated antibody in fixed U2OS cells

1. ~ 10,000 U2OS cells/well in 200μL media were seeded in the glass bottom Lab-Tek chamber (8 wells, Eppendorf).
2. After 24 hours of cell seeding removed and washed twice with PBS.
3. Then, cells were fixed with chilled methanol for 7 minutes at –20°C, followed by washing with PBS three times.
4. Blocking with 3% bovine serum albumin (BSA) in PBS at 24°C for 2 h.
5. Cells were incubated for 24 h at 4°C with primary antibody against microtubules (10 μg ml^{-1}) diluted in PBS containing 3% bovine serum albumin.
6. Excess antibody was removed by three times washing with PBS (with 5 min incubation each time).
7. A secondary antibody (donkey anti-rat-XYL-PC2) was irradiated with a UV lamp to cleave the PC2 linker for 5 minutes in an Eppendorf tube.

8. Cells were incubated with photocleaved XYL conjugated secondary antibodies (donkey anti-rat-XYL-PC2) (10 μg ml^{-1}) diluted in PBS containing 3% bovine serum albumin for 2 h.
9. Excess secondary antibody was removed by three times washing with PBS (with 5 min incubation each time).
10. [CB7–FL·XYL–BHQ3] quenched probes (Final fluorophore concentration: 1 mM CB7 and 3mM of XYL-BHQ3) were incubated with the ADA-labeled cells and immediately proceeded for non–covalent fluorogenic imaging.

8.4 Microtubule imaging in fixed BS-C-1 cells with CB7-FL and p-Xylylenediamine.2HCl washing

1. ~10,000 BS-C-1 cells/well in 200μL media were seeded in the glass bottom Lab-Tek chamber (8 wells, Eppendorf). After 24 hours of cell seeding, culture media were removed and washed twice with PBS.
2. Cells were fixed with chilled methanol for 7 minutes at –20°C, followed by washing with PBS three times.
3. Blocking with 3% bovine serum albumin (BSA) in PBS at 24°C for 2 h.
4. Cells were incubated for 24 h at 4°C with primary antibody against microtubules (10 μg ml^{-1}) diluted in PBS containing 3% bovine serum albumin.
5. Excess antibody was removed three times by washing with PBS (with 5 min incubation each time).
6. A secondary antibody (donkey-anti-rat-ADA-PEG) was irradiated with a UV lamp to cleave the PC2 linker for 5 minutes in an Eppendorf tube.
7. Cells were incubated with photocleaved ADA conjugated secondary antibodies (donkey-anti-rat-ADA-PEG) (10 μg ml^{-1}) diluted in PBS containing 3% bovine serum albumin for 2 h.
8. Excess secondary antibody was removed three times by washing with PBS (with 5 min incubation each time).
9. 1 μM of CB7–SiR was incubated with the ADA-labeled cells for 30 minutes, washed 3 times with 1× PBS with 2-minute interval and immediately proceeded for imaging.
10. Then 2.5 μM of p-Xylylenediamine.2HCl in 1× PBS was incubated in the same well for 30 minutes, washed 3 times with 1× PBS with 2-minute interval and immediately proceeded for imaging.

8.5 Cell fixation, immunostaining, and fluorogenic imaging protocol for nuclear pore complex

1. ~ 10,000 U2OS cells/well in 200μL media were seeded in the glass bottom Lab-Tek chamber (8 wells, Eppendorf). After 24 hours of cell seeding, culture media was removed, and cells were washed twice with PBS.

2. U2OS cells were fixed using 4% PFA for 15 min at room temperature (RT).

3. PFA was removed by washing with PBS three times. Afterward, free aldehyde groups were reduced with 1% NaBH4 (1 mg /ml) in PBS for 5 min.

4. After rinsing three times with PBS, cells were permeabilized using 0.25% (v/v) triton–X 100 in PBS for 10 min at RT.
5. Cells were washed three times with 1× PBS.
6. Blocking using 3% BSA (w/v) and 0.1% v/v Triton X-100 in PBS for 2 h at 24°C.
7. Cells were incubated overnight at 4°C with primary antibody against nuclear pore complex (NUP-98) (10 μg ml^{-1}) diluted in PBS containing 3% BSA and 0.1% v/v Triton X-100.
8. Excess antibody was removed by three times washing with PBS (with 5 min incubation each time).
9. Cells were incubated with ADA-conjugated secondary antibodies (10 μg ml^{-1}) diluted in PBS containing 3% BSA and 0.1% v/v Triton X-100 for 2 h.
10. Excess secondary antibody was removed by three times washing with PBS (with 5 min incubation each time).
11. [CB7–SiR·XYL–BHQ3] quenched probes (fluorophore concentration: 1 mM; quencher concentration: 3 mM) were incubated with the ADA labeled cells and immediately proceeded for non–covalent fluorogenic imaging.

8.6 Immunostaining and fluorogenic imaging protocol in live cell

1. A431/3T3 cells (~10,000 in 200 ml culture media) were plated in the 35 mm imaging dish with 10 mm glass bottom well (Cellvis, USA) and allowed to reach up to 60% confluence.
2. Immunostaining of live cells was done after keeping the cells at ~4°C.
3. Culture media was removed carefully, and cells were washed with 100 ml DPBS (pH 7.4) two times.
4. Cells were incubated with 100 ml primary antibody against EGFR (human, 10 mg ml^{-1}) in DPBS for 30 min at 4°C.
5. Excess antibody was removed by washing the cells using 100 ml DPBS (pH 7.4) two times.
6. Then cells were incubated with 100 ml ADA conjugated donkey anti-human secondary antibody (10 mg.ml^{-1}) in PBS for 30 min at 4°C.
7. Excess secondary antibody was removed by washing the cells using 100 ml DPBS (pH 7.4) two times.
8. [CB7–FL·XYL–Q] quenched probes (Final fluorophore concentration: 1 mM and XYL–Q concentration was varied according to the Table S6) were incubated with the ADA labeled cells and immediately proceeded for non–covalent fluorogenic imaging maintaining the live-cell imaging conditions.

8.7 Protocol for dissection and labeling of actins in muscle tissue from *Drosophila melanogaster*

Drosophila melanogaster cultures maintained under 12 h light: 12 h dark cycles at 25°C were used. Adult flies were collected and kept on ice for around 15 minutes for anesthetizing.

8.7.1 Thoracic muscle dissection

1. After flies were anesthetized, they were submerged in PBS, placed dorsally on the dissection plate, and were pierced with insect pins on the abdomen region.
2. Using forceps, the exoskeleton of the thorax was incised carefully, peeled out gently and a bunch of clustered thoracic muscles was taken out.
3. Dissected muscle tissues were transferred in chilled PBS in labeled wells of the glass dish kept on ice and were allowed to settle.

8.7.2 Fluorogenic imaging protocol for thoracic muscle tissue

Tissues were fixed with 4% paraformaldehyde (PFA) at 24°C for 30 minutes with gentle shaking.

1. Tissues were washed at least thrice with 5 minutes of incubation using PBS containing 0.5% Triton X–100 (0.5% PBT).
2. Samples were then blocked using 10% horse serum in 0.5% PBT for 1 h at 24°C.
3. Phalloidin–ADA (2 μM) was added to each well and incubated for 2 h at 24°C.
4. Afterwards, samples were washed three times with 0.5% PBT for 5 min incubation each time.
5. [XYL–Q · CB7–FL] quenched probes (Final fluorophore concentration: 1 mM) were incubated with the ADA labeled tissues and immediately proceeded for non–covalent fluorogenic imaging.

8.7.3 Dissection of intestine tissue from *Drosophila melanogaster* for live tissue imaging

1. Third instar larvae of *Drosophila melanogaster* which were maintained in 12:12 hour light: dark and constant 25°C were scooped out from culture vials and kept on chilled PBS for around 15 minutes to anesthetize.
2. Individual larvae were submerged in PBS in a glass dish.
3. Using forceps, the mouth hooks of larvae were held firmly, while with the help of another pair of forceps the larva was held at about 3/4th of the body length.
4. The posterior part of the body was gently pulled apart and separated from the anterior portion.
5. A small region of the gut corresponding to the midgut was isolated and placed in chilled PBS in a fresh well.
6. The gut was then incised with the help of fine scissors to expose and flatten the inner layer of tissue.

8.7.4 Protocol for fluorogenic labeling of microtubules using ADA-taxol in live intestine tissue.

1. Dissected intestinal tissues were incubated with ADA-taxol conjugate (2 mM) in PBS medium for 90 min at 24°C.
2. Isolated tissues were washed twice with PBS with 1 min incubation each.
3. [XYL–Q · CB7–FL] quenched probes (Final fluorophore concentration: 1 mM in Schneider's medium) were incubated with the ADA labeled tissues and immediately proceeded for non–covalent fluorogenic imaging using structured illumination microscopy (SIM) method.

8.8 Protocol for fluorogenic multiplexed imaging in fixed cells

1. MEF cells were cultured and fixed using 4% PFA for 15 min at 24°C.
2. Fixature were removed and washed with 1× PBS three times (5 minute).
3. Permeabilization using 0.25% (v/v) triton–X 100 in PBS 10 min at 24°C.
4. Cells were washed three times with 1× PBS.
5. Blocking using 3% BSA (w/v) in PBS for 2 h at 24°C.
4. Cells were incubated for 24 h at 4°C with primary antibody against microtubules (10 μg ml^{-1}) diluted in PBS containing 3% bovine serum albumin and 0.1% Triton–X 100.
5. Excess antibody was removed by three times washing with PBS (with 5 min incubation each time).
6. Cells were incubated with ADA conjugated secondary antibodies (10 μg ml^{-1}) diluted in PBS containing 3% bovine serum albumin and 0.5 mM Phalloidin–TCO conjugate for 2 h.
7. Excess secondary antibody and phalloidin were removed by three times washing with PBS (with 5 min incubation each time).
8. [CB7–TAMRA·XYL-BHQ2] quenched probes (Final fluorophore concentration: 1 mM) and tetrazine–Bodipy probe (final concentration: 0.5 mM) were incubated with the ADA and TCO labeled cells for 5 min and then proceeded for fluorogenic multiplexed imaging of microtubules and actin.

8.9 Protocol for fluorogenic imaging of microtubules using ADA-taxol and XYL decorated AuNPs (XYL-AuNPs) in live HeLa cells.

1. Approximately 10,000 HeLa cells were seeded into a 35 mm imaging dish with a 10 mm glass bottom well (Cellvis, USA) containing 200 μl of culture media.
2. After 24 hours, the culture media was carefully aspirated, and the cells were washed twice with 200 μl of DPBS.
3. The cells were then treated with ADA-taxol conjugate (10 μM) in complete cell culture DMEM media at 37°C for 4 hours, supplemented with verapamil hydrochloride (10 μM).
4. Following the incubation, the cells were washed three times with DPBS (pH 7.4).

5. Next, the [CB7-fluorophore· XYL-AuNP] quenched complex was added to the ADA-labeled cells. The final concentrations were as follows: CB7–TAMRA (1 μM) with XYL–AuNP (200 nM), CB7-Bodipy (1 μM) with XYL–AuNP (200 nM), and CB7-SiR (2 μM) with XYL–AuNP (200 nM). This step was carried out in cell culture DMEM media (phenol red free) for 60 minutes at 37°C in the 35 mm imaging dishes.
6. After 60 minutes, imaging was conducted without removing the excess probe, while maintaining live-cell imaging conditions using SIM and confocal microscopy.
7. During the live-cell imaging study, microtubule dynamics were monitored for 15 minutes, with images captured at 5-minute intervals, utilizing CB7–TAMRA and XYL–AuNP under the maintained live-cell setup conditions on the microscope.

8.10 Protocol for fluorogenic multiplexed imaging in live tissues

1. After dissections, live intestine tissues were incubated with ADA-taxol (2 mM) and Jasplakinolide–TCO conjugate (0.5 mM) in PBS medium for 90 min at 24°C.
2. Isolated tissues were washed twice with PBS with 1 min incubation each.
3. [XYL–BHQ2 · CB7–TAMRA] quenched probes (Final fluorophore concentration: 1 mM in Schneider's medium) and tetrazine–Bodipy probe (final concentration: 0.5 mM) were incubated with the ADA and TCO labeled tissues for 5 min and then proceeded for fluorogenic multiplexed imaging.

8.11 Protocol for control experiments in cells – Comparing ADA guest-modified targeting ligands to direct fluorophore-conjugated targeting agents.

A. Microtubule target

1. MEF cells were cultured and fixed using 4% PFA for 15 min at 24°C.
2. PFA was removed by washing with PBS three times.
3. Afterward, free aldehyde groups were reduced with sodium borohydride ($NaBH_4$) (1 mg/ml) in PBS for 5 min.
4. After rinsing three times with PBS, cells were permeabilized using 0.25% (v/v) triton–X 100 in PBS for 10 min at 24°C.
5. Cells were washed three times with 1× PBS.
6. Blocking using 3% BSA (w/v) in PBS for 2 h at 24°C.
2. Cells were incubated for 24 h at 4°C with primary antibody against microtubules (10 μg ml^{-1}) diluted in PBS containing 3% bovine serum albumin and 0.25 % Triton–X 100.
3. Excess antibody was removed by three times washing with PBS (with 5 min incubation each time).

4. Cells were incubated with a mixture of ADA conjugated secondary antibodies (10 µg ml^{-1}) and Alexa647 conjugated secondary antibodies (3.33 µg ml^{-1}) diluted in PBS containing 3% bovine serum albumin and 0.25 % Triton–X 100.
5. Excess secondary antibody was removed by three times washing with PBS (with 5 min incubation each time).
6. [CB7–Bodipy·XYL-BHQ1] quenched probe (Final fluorophore concentration: 1 mM) was incubated for 15 min and washed. After that, we proceeded with two-color SIM imaging of microtubules.

B. Actin target

1. MEF cells were fixed using the same above protocol (A).
2. Cells were incubated with a mixture of ADA-Phalloidin (1 µM) and Alexa488 conjugated phalloidin (0.33 µM) diluted in PBS containing 3% bovine serum albumin and 0.25 % Triton–X 100.
3. Excess Alexa488-Phalloidin and ADA-Phalloidin were removed by three times washing with PBS.
4. [CB7–Cy5·XYL-BHQ3] quenched probe (Final fluorophore concentration: 1 mM) was incubated for 5 min and washed. After that, we proceeded with two-color SIM imaging of actin.

8.12. Metabolic lebeling and fluorogenic imagin of bacteria

8.12.1 Bacterial culture

1. The metabolic labeling experiments utilized the following bacterial strains: *Mycobacterium smegmatis* (Msmeg, mc^2155), GFP-Msmeg[7] (mc^2155 transformed with pGFPHYG2 (Addgene plasmid # 30173)), and *Mycobacterium tuberculosis* (H37Ra). Control experiments were conducted using the *E. coli* (DH5-alpha) strain.

2. Msmeg and H37Ra cultures were grown in 7H9 media supplemented with 10% ADC growth factor and 0.1 % Tween 80. *E. coli* was cultured in LB medium at 37°C in a shaking incubator (180 RPM).

3. Bacterial strains were inoculated from glycerol stocks and cultured at 37°C with shaking to reach the logarithmic phase of growth, indicated by an OD_{600} of approximately 0.6. For *E. coli*, 8 µL from the stock was added to 5 mL of fresh media and incubated for approximately 8 h. For Msmeg and GFP-Msmeg, the same procedure was followed, with an incubation time of around 29 h. The incubation time for H37Ra was 42 h.

4. Once the bacteria reached the logarithmic growth phase, they were used for conducting metabolic labeling experiments. The required number of bacterial cells from the starter culture was diluted in a fresh growth medium containing the desired compounds in 96-well plates for specific experiments.

8.12.2 Metabolic labeling experiment involving Msmeg, GFP-Msmeg, and Mtb H37Ra, alongside the control strain of *E. coli* (DH5-alpha)

Metabolic incorporation in Msmeg and GFP-Msmeg

1. The experiments were carried out using a flat-bottom 96-well transparent plate.

2. A 45 mM stock of Tre-ADA in DMSO was diluted in the culture media to achieve the desired concentration of Tre-ADA.

3. A mixture of 15 µL of starter bacteria culture and 185 µL of culture media containing the desired concentration of Tre-ADA was prepared in the 96-well plate.

4. Two control samples were additionally prepared as follows: (a) 15 µL of bacteria mixed with 185 µL of media containing the vehicle control (DMSO). (b) 15 µL of bacteria mixed with 185 µL of media alone was used as another control.

5. The 96-well plate was incubated in a microplate reader at 37°C with shaking until the desired OD_{600} (approximately 0.6) was reached. A damp paper towel was placed inside the device along with the plate to prevent evaporation. Typically, it took approximately 16 h to reach the desired OD_{600}. The bacterial growth curve is shown in Supporting Figure S18.

6. After reaching the desired OD_{600}, the metabolically ADA incorporated samples and the control samples were utilized for fluorogenic staining experiments.

7. The bacterial solution was transferred to a microcentrifuge tube and washed three times with PBS-B (1×PBS + 0.5% BSA) by centrifugation at 3000 rpm for 3 minutes.

8. CB7–SiR and XYL–BHQ3, mixed in 10 µL of milli-Q water and diluted in PBS-B solution, were added to the metabolically incorporated bacteria and the control bacterial solution. The final concentrations were CB7–SiR= 1µM and XYL–BHQ3= 2.5µM. This solution was then used for the imaging experiment without removing the excess probe solution.

Control experiment with E. coli (DH5-alpha)

1. The experiments were carried out using a flat-bottom 96-well transparent plate.

2. A 45 mM stock of Tre-ADA in DMSO was diluted in the culture media to achieve the desired concentration of Tre-ADA.

3. A mixture of 15 µL of starter bacteria culture and 185 µL of culture media containing the desired concentration of Tre-ADA was prepared in the 96-well plate.

4. Control sample was additionally prepared as follows:15 µL of bacteria mixed with 185 µL of media alone.

5. The 96-well plate was incubated in a microplate reader at 37°C with shaking until the desired OD_{600} (approximately 0.6) was reached. A damp paper towel was placed inside the device along with the plate

to prevent evaporation. Typically, it took approximately 8h to reach the desired OD_{600}. The bacterial growth curve is shown in Supporting Figure S19.

6. After reaching the desired OD_{600}, the bacterial solution was transferred to a microcentrifuge tube and washed three times with PBS-B (1×PBS + 0.5% BSA) by centrifugation at 3000 rpm for 3 minutes.

8. CB7–SiR and XYL–BHQ3, mixed in 10 µL of milli-Q water and diluted in PBS-B solution, were added to the bacterial solution. The final concentrations were CB7–SiR= 1µM and XYL–BHQ3= 2.5µM. This solution was then used for the imaging experiment without removing the excess probe solution.

Metabolic incorporation in Mtb H37Ra

1. A 45 mM stock of Tre-ADA in DMSO was diluted in the culture media to achieve the desired concentration of Tre-ADA.

3. A mixture of 50 µL of starter bacteria culture and 150 µL of culture media containing the desired concentration of Tre-ADA was prepared in a microcentrifuge tube.

4. Control sample was additionally prepared as follows: 50 µL of bacteria mixed with 150 µL of media alone.

5. The bacterial solution was incubated in an incubator shaker at 37°C for 42 h.

6. Afterwards, the bacterial solution was washed three times with PBS-B (1×PBS + 0.5% BSA) by centrifugation at 1220 g rpm for 3 minutes.

8. CB7–SiR and XYL–BHQ3, mixed in 10 µL of milli-Q water and diluted in PBS-B solution, were added to the bacterial solution. The final concentrations were CB7–SiR= 1µM and XYL–BHQ3= 2.5µM. This solution was then used for the imaging experiment without removing the excess probe solution.

8.12.3 Imaging of bacterial cells

1. [CB7–SiR·XYL–BHQ3] added bacterial solutions were incubated for 30 min in a shaker at 25°C.
2. Agarose pads were prepared using 1.5 % agarose in 1× PBS.
3. Bacterial solutions were dropped on the agarose pad and uniformly distributed.
4. The agarose pad was then inverted on a #1.5 microscope cover glass for imaging.
5. The samples were imaged using confocal and SIM setups.

9. Microscopy setup

Epi–fluorescence microscopy via non–covalent fluorogenic labeling was carried out using an inverted microscope (Olympus) equipped with a cool–LED light source and a CMOS camera. Whereas Structured illumination microscopy (SIM) was carried out using an inverted Zeiss ELYRA PS1 microscope equipped with 4 excitation lasers and an sCMOS camera. For epi–fluorescence microscopy of fixed cell and tissue samples, the imaging dishes were placed under the microscope, and images were captured in epi–

fluorescence method using an Olympus oil–immersion objective (Plan–apochromat DIC 63x/1.40 Oil). Four LED sources have been used for excitation: 365 nm, 490 nm, 550 nm, and 635 nm for the respective excitation of fluorophores. Fluorescence light was spectrally filtered with U-FUNA (Exciter filter BP 360-370 nm, Dichroic beamsplitter DM410 with barrier filter BA420-460) for excitation of 365nm, U-FBNA (Exciter filter BP 470-495 nm, Dichroic beamsplitter DM505 with barrier filter BA510-550) for excitation of 490 nm, U-FGWA (Exciter filter BP 530-550 nm, Dichroic beamsplitter DM570 with barrier filter BA575-625) for excitation of 550 nm, and Brightline Quad-band "Pinkel" Filter set (LED-DA/FI/TR/Cy5-4X-B-000) for excitation of 635 nm. Imaging was carried out using an optiMOS sCMOS camera (QImaging).

Live tissue samples kept in microscope imaging dishes were placed under the microscope maintained at 37°C and 5% CO_2 atmosphere and fluorescence microscopic images were captured by structured illumination method using an inverted Zeiss ELYRA PS1 microscope. Four lasers have been used for excitation: 405 nm (50 mW), 488 nm (200 mW), 561 nm (200 mW), and 642 nm (150 mW) for respective excitation of fluorophores. Details of imaging parameters for the individual experiments have been given in the table below. Imaging was performed using a Zeiss oil–immersion objective (alpha Plan–apochromat DIC 63×/1.40 Oil DIC M27, numerical aperture (NA) 1.40 oil). Fluorescence light was spectrally filtered with emission filters (MBS– 405+EF BP 420–495/LP 750 for laser line 405, MBS– 488+EF BP 495–570/LP 750 for laser line 488, MBS– 561+EF BP 570–650/LP 750 for laser line 561 and MBS–642+EF LP 655 for laser line 642) and imaged using a PCO edge sCMOS camera.

For confocal imaging, cells were imaged using a Leica TCS SP8 microscope. Two lasers were used for the experiments: 405 nm (source: 50 mW) and 552 nm (source: 20 mW). Imaging was performed using Leica oil–immersion objectives: HC PL APO CS2 63× with a numerical aperture (NA) 1.40. Excitation beam splitter – DD 488/552. Cells were imaged with a HyD detector (408nm-466 nm) – Gain 100 and a PMT detector (569nm-651nm) – Gain 750. Confocal images were processed using LAS X (Leica) and ImageJ software.

Table S7: Table for imaging parameters in SIM experiment.

**Lasers**	**Fluorophores used**	**Exposure time (ms)**	**Laser power density ($W.cm^{-2}$)**
405	Coumarin	100	1.22
488	Fluorescein, Bodipy	100	6.51
561	Cy3, TMR	100	7.32
642	Cy5, SiR	100	2.93

Table S8: Table for imaging parameters of Epi-fluorescence microscopy for Figure 2 – 5.

LED Source	Fluorophores used	Exposure time (ms)	LED intensity (Percentage, %)
365 nm	Coumarin, DAPI	100	25
490 nm	Fluorescein, Bodipy	100	20
550 nm	Cy3, TMR	100	20
635 nm	Cy5*, SiR	100	20

* In the case of imaging using Cy5 and DAPI channel, the DAPI parameter used is 25% LED intensity and 40 ms exposure time.

10. Image processing and data analysis

Epi–fluorescence microscopy images of fixed cells were used as acquired after adjusting the brightness using ImageJ and data analysis was carried out using GraphPad prism. Other epifluorescence images from fixed tissues and live cells were subjected for deconvolution using cellSens software (Olympus) pre-installed with constrained iterative deconvolution mathematical algorithm function. Structured illumination image was reconstructed using a structured illumination analysis package for Zen 2.0 software (Zeiss). Additional software has been used for color adjustment (ImageJ) and data analysis (Origin 9.0 and GraphPad Prism).

Table S9: Experimental set-up for imaging of cells in Figures 2 and 3.

Figures	Mode of imaging	Targeting molecules (TM)		Cell/Tissue	Exposure time
		TM – 1	TM – 2		
Figure 2c & 3	Epi-fluorescence microscopy	α-tubulin (rat)	ADA conjugated donkey anti-rat	MEF cell	100 ms

Table S10: Experimental set-up for imaging of tissues in Figure 4.

Figures	Mode of imaging	Targeting molecules (TM)	Cell/Tissue	Exposure time
Figure 4a	SIM	ADA-Phalloidin	MEF cell	100 ms
Figure 4b	Epi-fluorescence microscopy	ADA-Phalloidin	Thoracic muscle tissue from *Drosophila melanogaster*	100 ms

Table S11: Experimental set-up for Epi/TIRF/SIM imaging of cells and tissues in Figures 5,6, and 7.

Figures	Mode of imaging	Targeting molecules (TM)		Cell/Tissue	Exposure time
		Primary	Secondary		
Figure 5	Epi-fluorescence microscopy	Human EGFR Antibody	ADA conjugated donkey anti-human	A431 cell	100 ms
Figure 6b	TIRF	ADA-taxol		*In-vitro* polymerized microtubules	100 ms
Figure 7	SIM	ADA-taxol		Live intestine tissue	100 ms

Table S12: Experimental set-up for SIM imaging of *GFP-Msmeg* in Figure 8.

Figures	Mode of imaging	Targeting molecules (TM)	Strain of Bacteria	Exposure time
Figure 8e	SIM	Trehalose-ADA	*GFP-Msmeg*	100 ms

Table S13: Experimental set-up for imaging of cells and tissues in Figure 9.

Figures	Mode of imaging	Targeting molecules (TM)		Cell/Tissue	Exposure time
		Primary	Secondary		
Figure 6b	SIM	1. α-tubulin (rat)	ADA conjugated donkey anti-rat	MEF cell	100 ms
		2. Phalloidin-TCO			
Figure 6c	SIM	1. ADA-taxol<br>2. Jasplakinolide–TCO		Live intestine tissue	100 ms

Table S14: Experimental set-up for confocal imaging in Figure 6 and 8.

Figures	Mode of imaging	Targeting molecules (TM)	Cell/Tissue	Scan Speed (Hz)	Line average	Scan Format
Figure 6f	Confocal	ADA-taxol	HeLa (Bodipy)	400	8	1024*1024
Figure 6f	Confocal	ADA-taxol	HeLa (TAMRA)	400	8	1024*1024

Figure 6f	Confocal	ADA-taxol	HeLa (SiR)	400	16	1024*1024
Figure 6g	Confocal	ADA-taxol	HeLa (TAMRA)	400	5	1024*1024
Figure 8c	Confocal	Trehalose-ADA	*Msmeg*	400	16	1024*1024
Figure 8d	Confocal	Trehalose-ADA	*E. coli*	400	16	1024*1024
Figure 8f	Confocal	Trehalose-ADA	*mtb H37Ra*	600	3	1024*1024

11. Protocol for kinetic study in the cell using confocal imaging.

MEF cells were fixed and labeled with ADA-antibody. Two experiments were performed with – 1. Fixed MEF cells without any immunostaining and 2. Fixed MEF cells labeled with ADA-antibody. Next, cells were incubated with quenched probe [CB7-TAMRA (1 μM) + XYL-BHQ2 (1.5 μM)] in both cases. The imaging was performed using a confocal microscope with a time interval of 3 min for only MEF cells and 1 min for ADA-antibody labeled cells over a duration of 30 min (Figure S4 and S9).

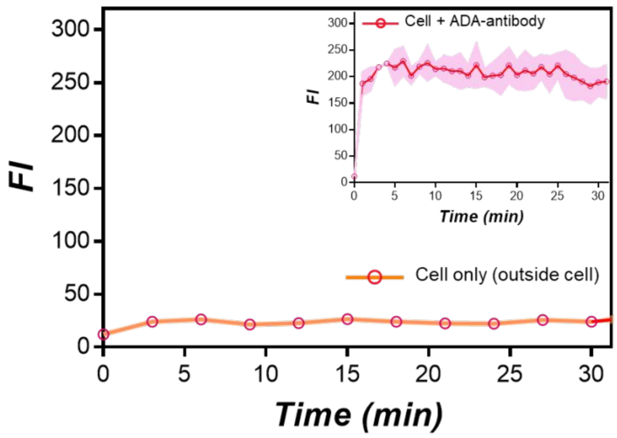

Figure S4: Time-lapse fluorescence study using only cells (without ADA labeling) demonstrates that the intensity of the cellular medium remains unchanged upon incubation with the host-guest quenched probe ([CB7–TAMRA·XYL–BHQ2]), confirming the stability of the quenched probes in cellular conditions. For comparison, the inset displays the kinetics of fluorogenic labeling using the quenched probe [CB7–TAMRA·XYL–BHQ2] in ADA immunolabeled cells, revealing a fluorescence increase within the cells upon specific interaction between the quenched probe [CB7–TAMRA·XYL–BHQ2] and ADA. The inset kinetics is detailed in Figure S9. The probe concentration was maintained as described in Table S6.

12. Supporting information images.

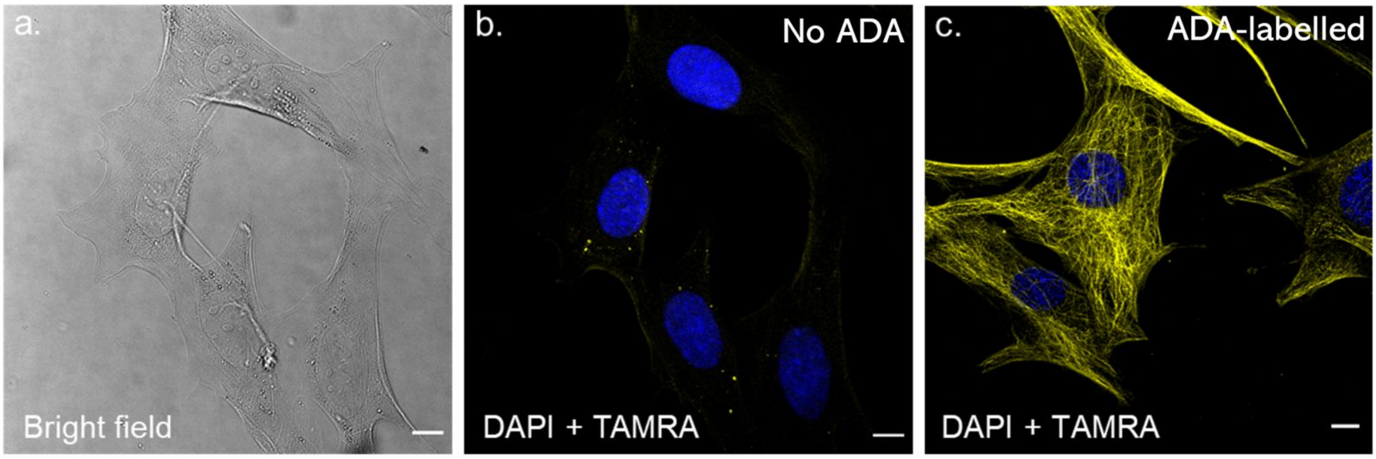

Figure S5: (a-b) Images of control cells (without ADA labeling) upon incubation with the host-guest quenched ([CB7–TAMRA·XYL–BHQ2]) probe. a) Brightfield and b) Fluorescence channel (TAMRA + DAPI). Negligible fluorescence signals from the cells indicated minimal off-target activation and labeling from the host-guest quenched probe. As a comparison, (c) shows an example of fluorogenic labeling using [CB7–TAMRA·XYL–BHQ2] in ADA labeled cells. MEF cells are labeled using protocol described in Section 10. Imaging was performed using a confocal microscope (Source – 405 nm for DAPI and 552 nm for TAMRA). Gain – 100, Laser intensity – 2% for DAPI and Gain – 750, and Laser intensity – 1% for TAMRA. Scale bar – 10 μm. The probe concentration was maintained as described in Table S6.

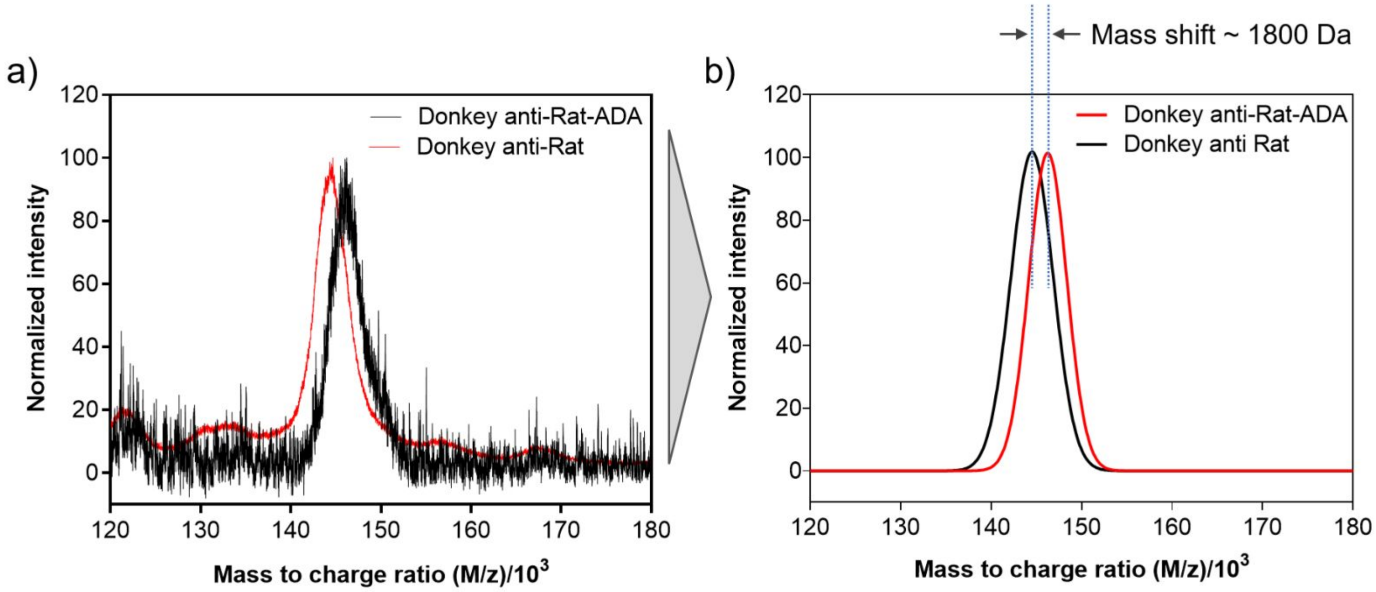

Figure S6: (a) MALDI-MS spectra of ADA modified Donkey Anti-Rat antibody (labeled as Donkey anti-Rat-ADA) and the corresponding unmodified version of the Donkey Anti-Rat antibody (labeled as Donkey anti-Rat). (b) Gaussian distribution fitting of the MALDI-MS data. We observed a mass shift of ~1800 Da after ADA conjugation reaction with the antibody. Given each addition of ADA molecule leads

to ~689.42 Da increase in mass, it was estimated that on an average ~2.6 molecules of ADA were attached per antibody.

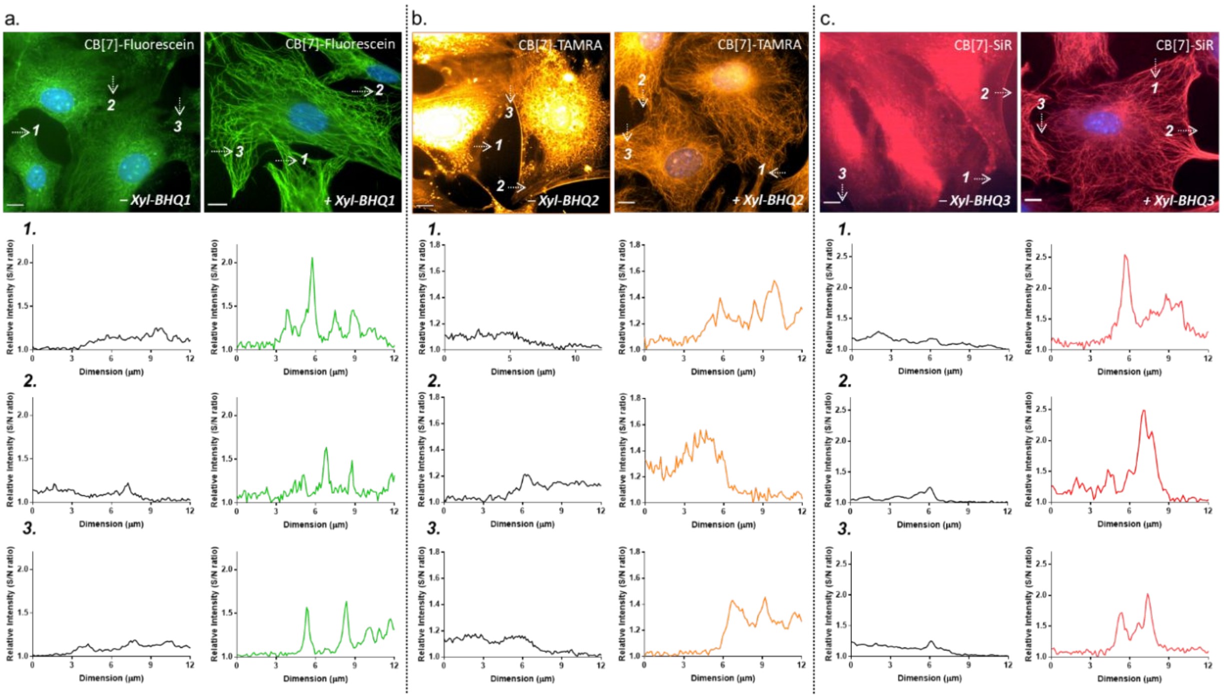

Figure S7: Intensity profile reporting single to noise (S/N) ratio for the fluorogenic imaging (CB7-FL·XYL-Q) as compared to only fluorophore-based imaging (CB7-FL) in ADA-labelled cells. a) CB7-Fluorescein with and without XYL-BHQ1, b) CB7-TAMRA with and without XYL-BHQ2, and c) CB7-SiR with and without XYL-BHQ3. The intensity profile was drawn along the arrows as shown in the images. Scale bar – 10 µm. MEF cells are labeled using the protocol described in Section 10 [The probe concentration was maintained as described in Table S6]. Imaging parameters are used as described in Table S8 (LED source – 365 nm for DAPI, 490 nm for Fluorescein, 550 nm for TAMRA, and 635 nm for SiR).

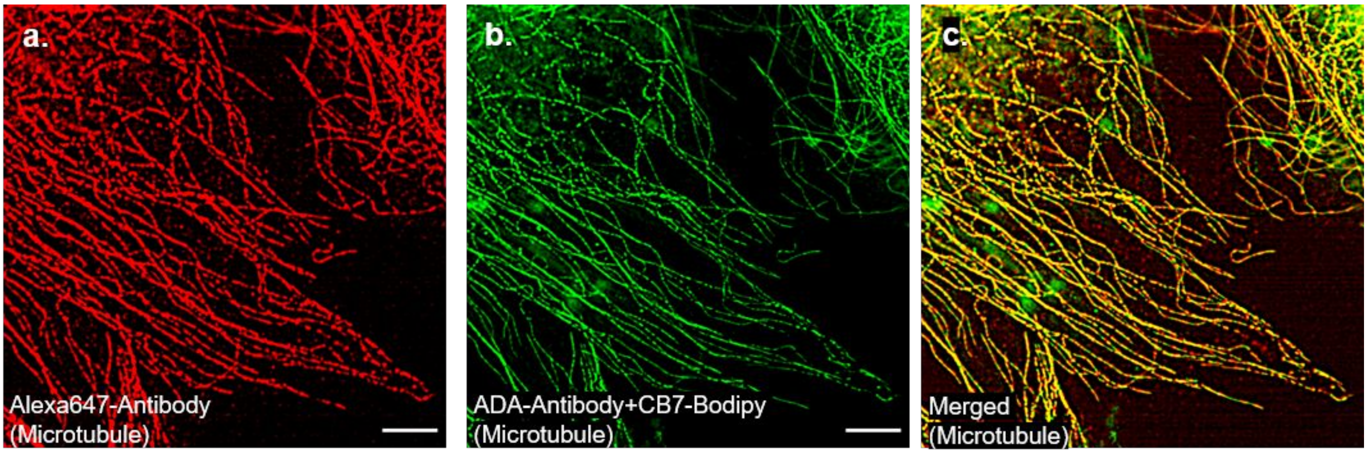

Figure S8: Control experiment comparing ADA guest-modified antibodies to direct fluorophore-conjugated antibodies. (a-c) Two-color SIM imaging of microtubules in MEF cells co-stained with two

different antibody conjugates. a) SIM image of the microtubules targeted using Alexa647-antibody (642 nm channel). b) SIM image of the microtubules targeted using ADA-antibody. CB7–Bodipy·XYL–BHQ1 complex is used to carry out the fluorogenic imaging of microtubules in the 488 nm channel (after washing). c) Merged image of microtubules. Scale bar – 5 μm. MEF cells are fixed and labeled using protocol as described above.

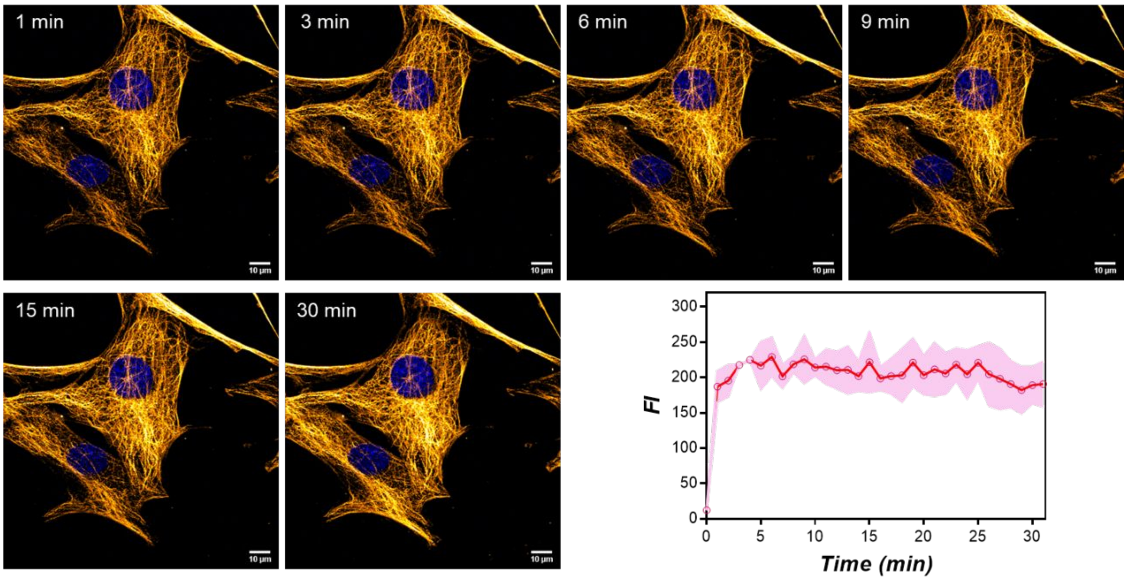

Figure S9: Kinetics of fluorogenic labeling using quenched probes in ADA labeled cells. Antibody-based ADA cells were treated with [CB7–TAMRA·XYL–BHQ2] quenched probe and time-lapsed images were immediately recorded to demonstrate the temporal intensity improvement from microtubule targets, which in turn exhibits the labeling kinetics on the imaging platform. The fluorescence intensity plot over time indicated the completion of the labeling within minutes. MEF cells are labeled using protocol described in Section 10. Imaging was performed using a confocal microscope (Source – 405 nm for DAPI and 552 nm for TAMRA). Gain – 100, Laser intensity – 2% for DAPI and Gain – 750, and Laser intensity – 1% for TAMRA. Scale bar – 10 μm. The probe concentration was maintained as described in Table S6.

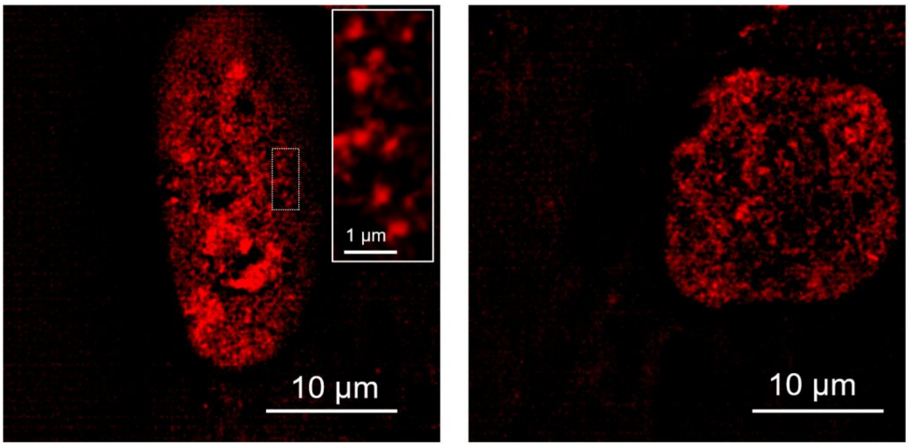

Figure S10: Fluorogenic imaging of NPC (nuclear pore complex) using host-guest complex. Fluorogenic imaging of NPC (nuclear pore complex) in U2OS cells labeled with NUP 98 antibody and ADA conjugated Donkey anti-Rat secondary antibody. Fluorogenic staining was carried out using [CB7–SiR·XYL–BHQ3] complex [CB7–SiR: 1μM and XYL–BHQ3: 3μM] before performing SIM imaging of the cells. The inset shows a magnified view of the NPC, which appears as distinct dotted puncta on the nuclear envelope.

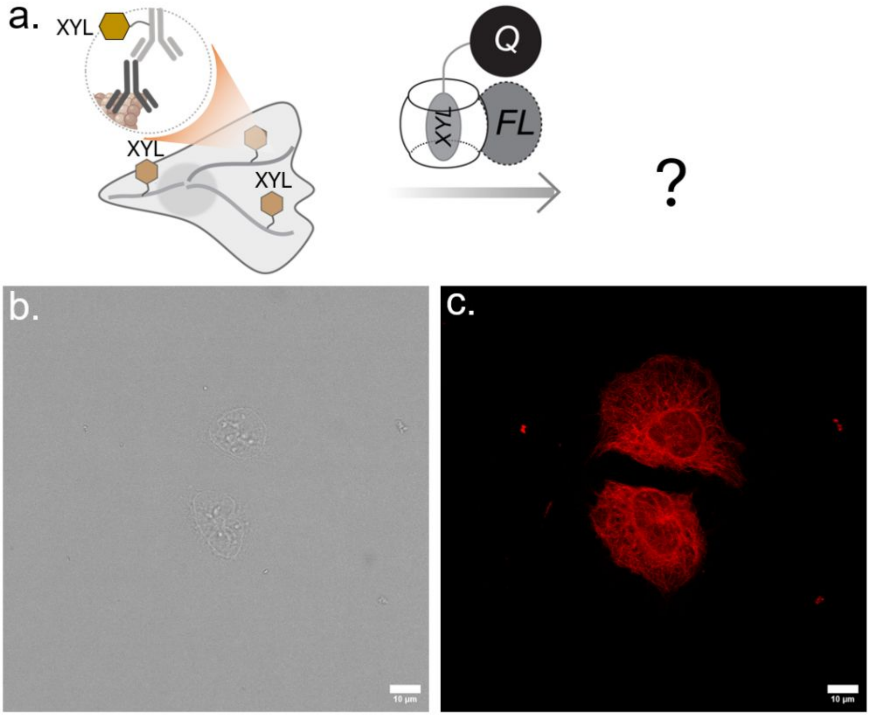

Figure S11: Labeling of microtubules in U2OS cells using XYL-conjugated antibody and fluorogenic probe CB7–SiR·XYL–BHQ3. (a) Cells were immunostained with an α-tubulin primary antibody and a donkey anti-rat secondary antibody conjugated with XYL. Subsequently, the fluorogenic probe CB7–SiR·XYL–BHQ3 was added [CB7–SiR: 1 μM and XYL–BHQ3: 3 μM] to the XYL-labeled cells. (b, c) Confocal laser scanning microscopy (CLSM) imaging was used to visualize the microtubules. (b) shows the

brightfield image, and (c) shows the fluorescence channel image with excitation at 638 nm. Scale bar: 10 μm.

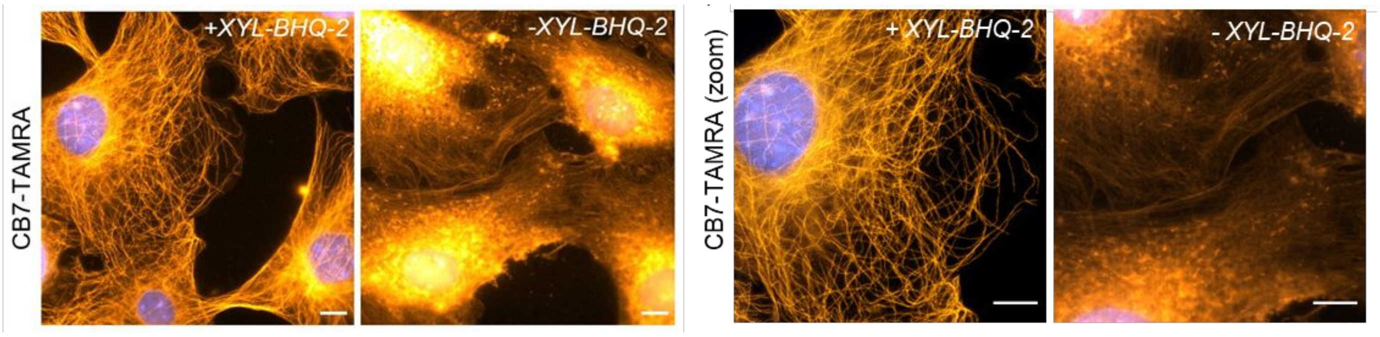

Figure S12: a) Fluorescence images acquired after washing excess probes demonstrate reduced off-target signal from the host-guest quenched probes due to its conditionally activable nature whereas, the absence of quenchers resulted in off-target binding of imaging probes. b) Zoomed image. Scale bar:10 mm (a–b). MEF cells are labeled using protocol described in Section 10 [The probe concentration was maintained as described in Table S6]. Imaging parameters are used as described in Table S8 (LED source – 365 nm for DAPI, 490 nm for Bodipy, 550 nm for TAMRA, and 635 nm for SiR).

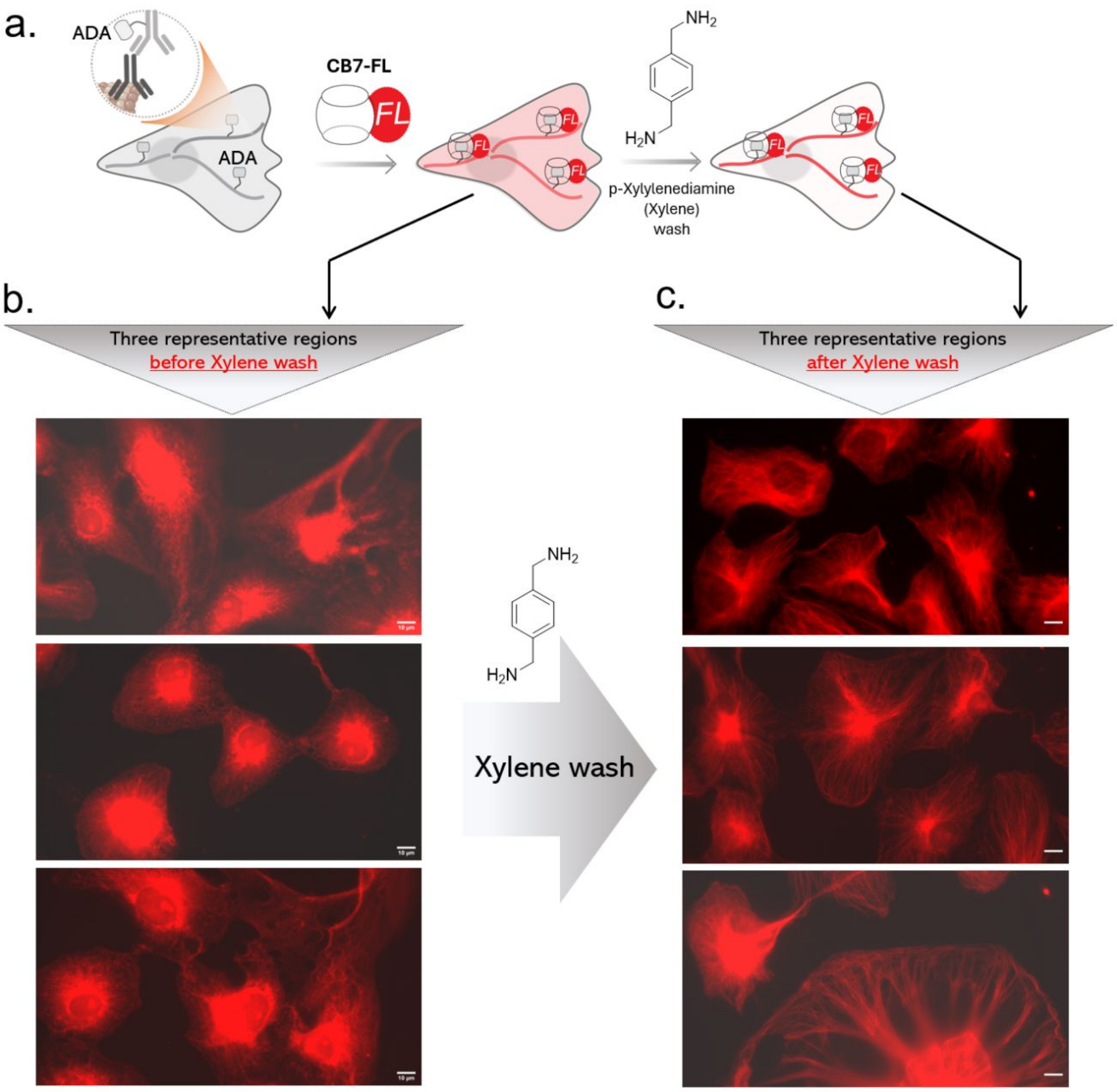

Figure S13: Removal of non-specifically bound CB7-FL using free Xylene guest washing. (a) α-Tubulin in BS-C-1 cells was labeled using an immunostaining approach with ADA-conjugated secondary antibody, which was then stained with CB7-SiR. Panel (b) shows three representative images of the cells before xylene wash, and panel (c) shows images after the Xylene wash. The microtubules were clearly visualized after washing the cells with Xylene. Scale bar: 10 μm.

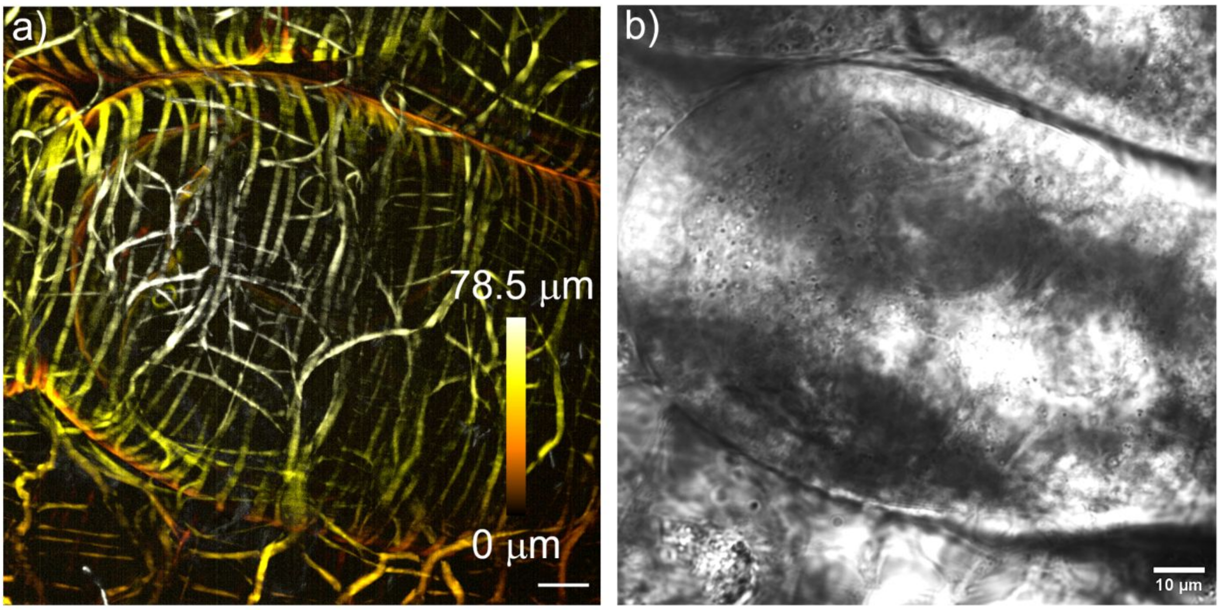

Figure S14: (a) 3D fluorogenic imaging of actins over 80 µm Z depth from ovary tissues of *Drosophila melanogaster.* Scale bar (XY): 10 µm. The probe concentration for CB7-TAMRA·XYL-BHQ2 is maintained as described in Table S6. (b) Bright field image of the same tissue section.

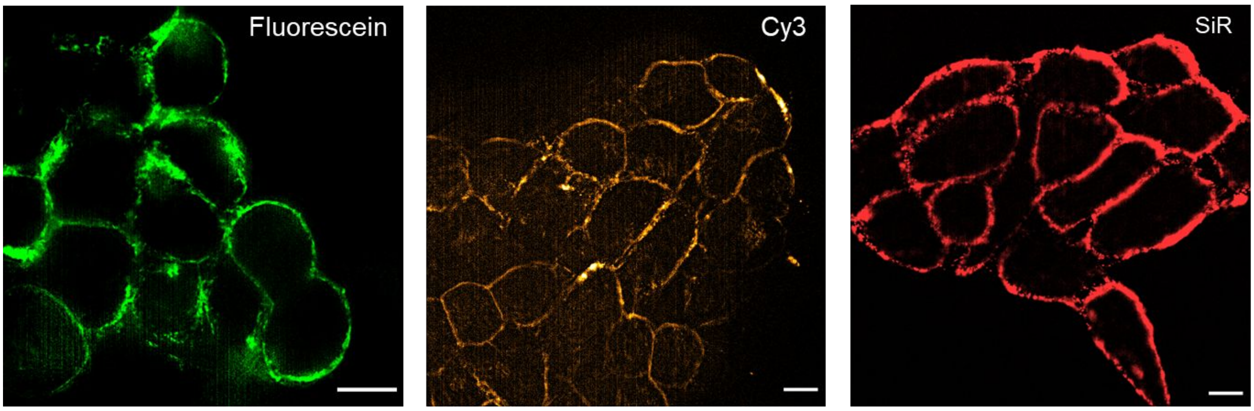

Figure S15: Additional images (with fluorescein, Cy3, and SiR host-guest probes) showing fluorogenic imaging of EGFR in live A431 cells. These images demonstrate the capability of fluorescein, Cy3, and SiR probes for supramolecular host-guest fluorogenic imaging in live cells. Scale bar – 10 µm. A431 cells are labeled using protocol described in Section 10 [The probe concentration was maintained as described in Table S6]. Imaging parameters are used as described in Table S8 (LED source – 490 nm for Fluorescein, 550 nm for Cy3, and 635 nm for SiR).

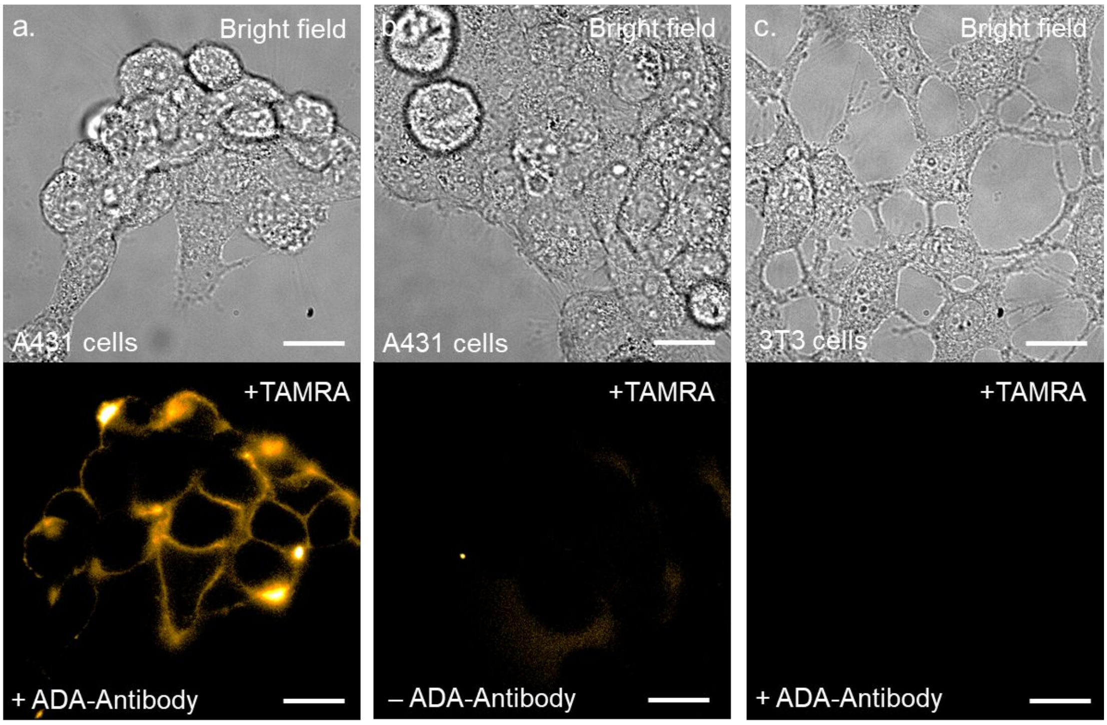

Figure S16: Fluorogenic imaging of EGFR and control experiments using [CB7–TAMRA·XYL–BHQ2] probe. a) EGFR labeling using ADA-Antibody in A431 cells. b) A431 cells without ADA-Antibody treatment show no EGFR labeling. c) EGFR negative 3T3 cells shows no fluorescence signal even with ADA-Antibody treatment [CB7-TAMRA: 1.0 μM and XYL-BHQ2 – 1.0 μM]. Scale bar – 20 μm. A431 and 3T3 cells are labeled using protocol described in Section 10. Excitation used – 550 nm for TAMRA having 20% intensity (LED source) with 30 ms exposure time.

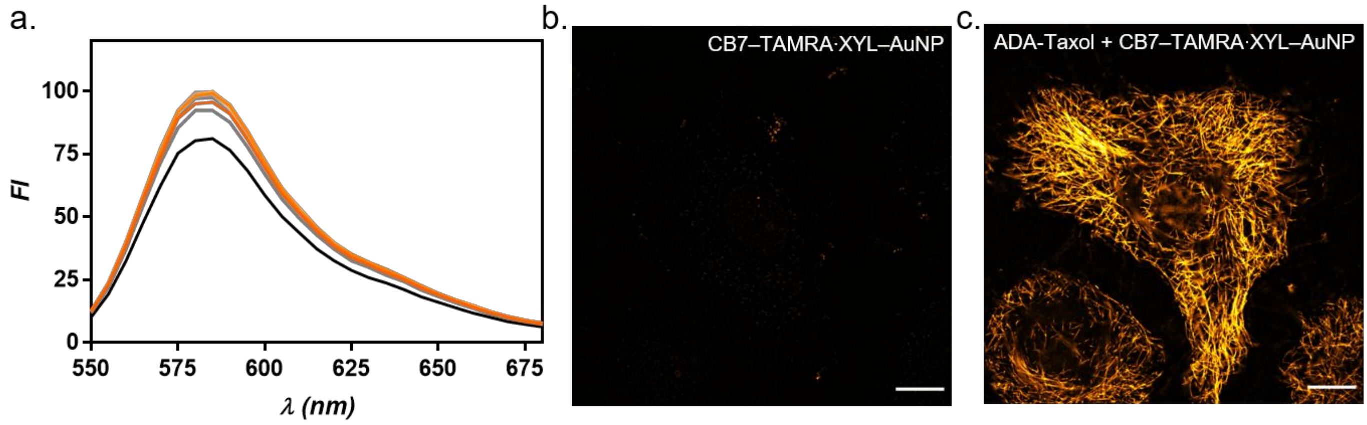

Figure S17: (a) Fluorescence titration data for TAMRA-ethanolamine (TAMRA-EtA) vs XYL-AuNP. Negligible fluorescence quenching from the non–interacting pair demonstrated that the fluorescence quenching requires specific recognition of CB7 host and XYL guest moiety. (b) SIM imaging of microtubules in live HeLa cells using CB7-FL·XYL-AuNP quenched probes without ADA-Taxol. (c)

Fluorogenic SIM imaging of microtubules in live HeLa cells using ADA-Taxol and CB7-FL·XYL-AuNP quenched probes. Scale bar: 10 μm.

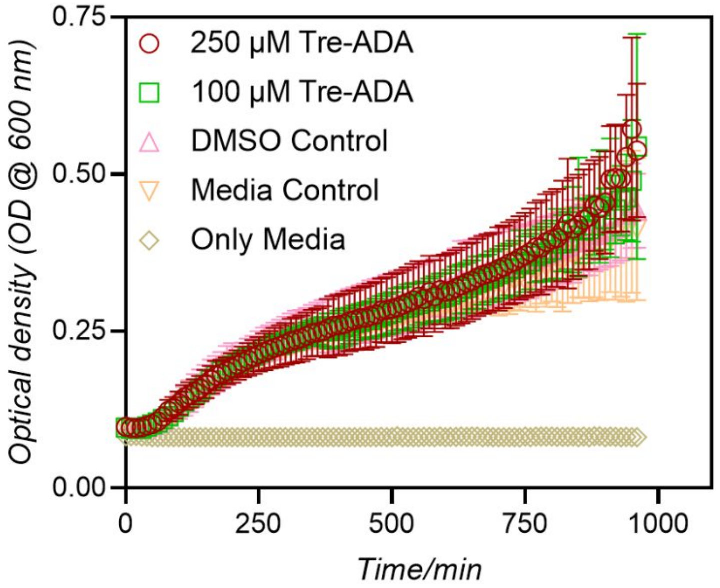

Figure S18: Representative growth curves of Msmeg under varying concentrations of Tre-ADA and control conditions. The growth curves were recorded using a plate reader at 37°C.

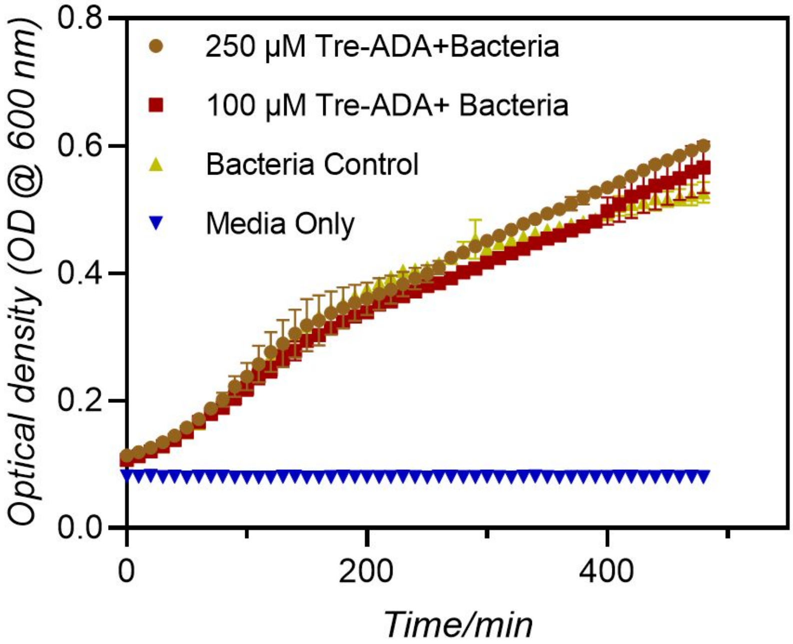

Figure S19: Representative growth curves of E. coli under varying concentrations of Tre-ADA and control conditions**.** The growth curves were recorded using a plate reader at 37°C.

13. HPLC, Mass, and NMR characterization data

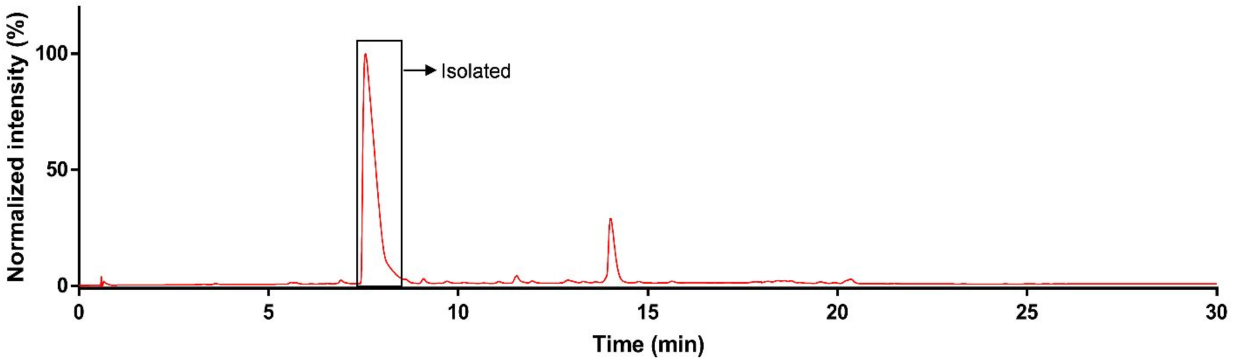

Figure S20: HPLC chromatogram of XYL–Dabcyl conjugate. The polarity of acetonitrile varied from 5 to 100% in 30 min. The conjugated product was isolated at retention time (R_t) 8.1 min.

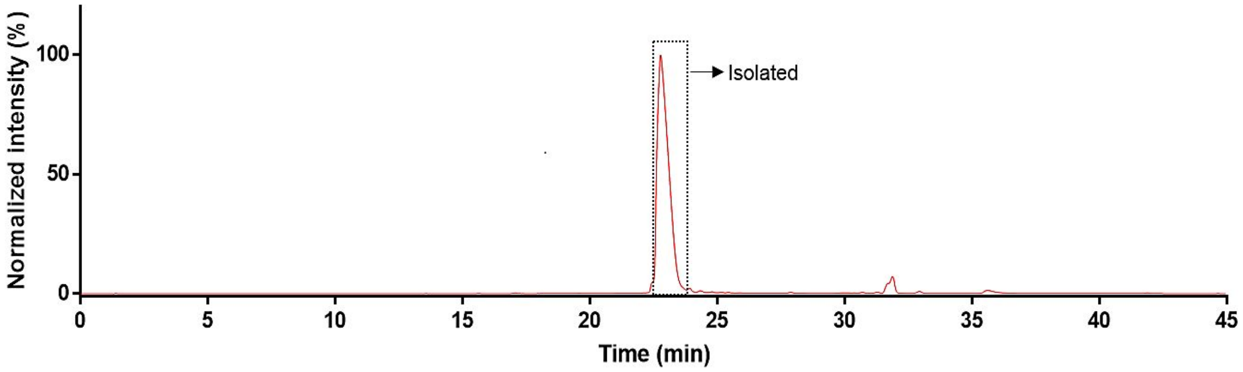

Figure S21: HPLC chromatogram of XYL–BHQ1 conjugate. The polarity of acetonitrile varied from 5 to 100% in 45 min. The conjugated product was isolated at retention time (R_t) 23.6 min.

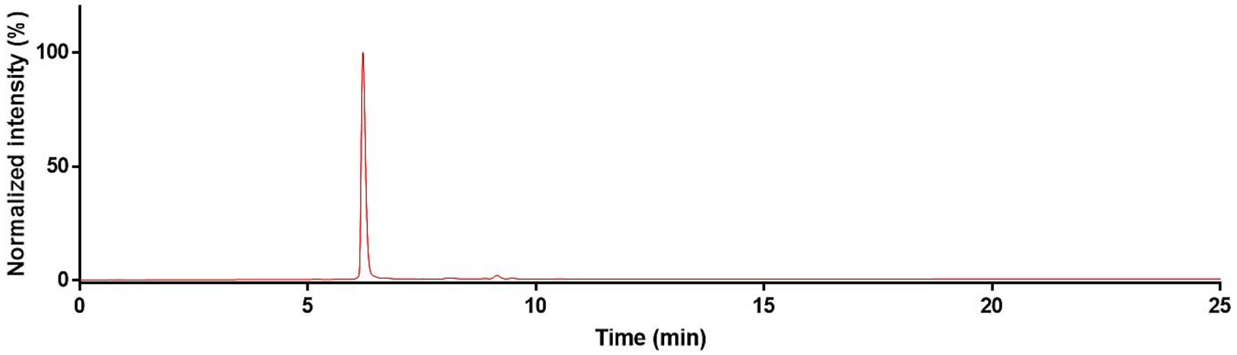

Figure S22: HPLC chromatogram of XYL–BHQ2 conjugate. The polarity of acetonitrile varied from 5 to 100% in 25 min. The conjugated product was isolated at retention time (R_t) 6.9 min.

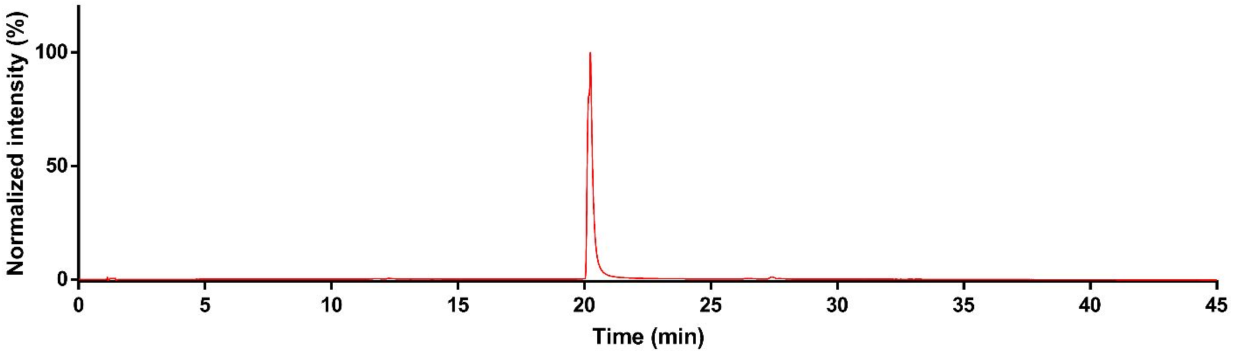

Figure S23: HPLC chromatogram of XYL–BHQ3 conjugate. The polarity of acetonitrile varied from 5 to 100% in 45 min. The conjugated product was isolated at retention time (R_t) 20.5 min.

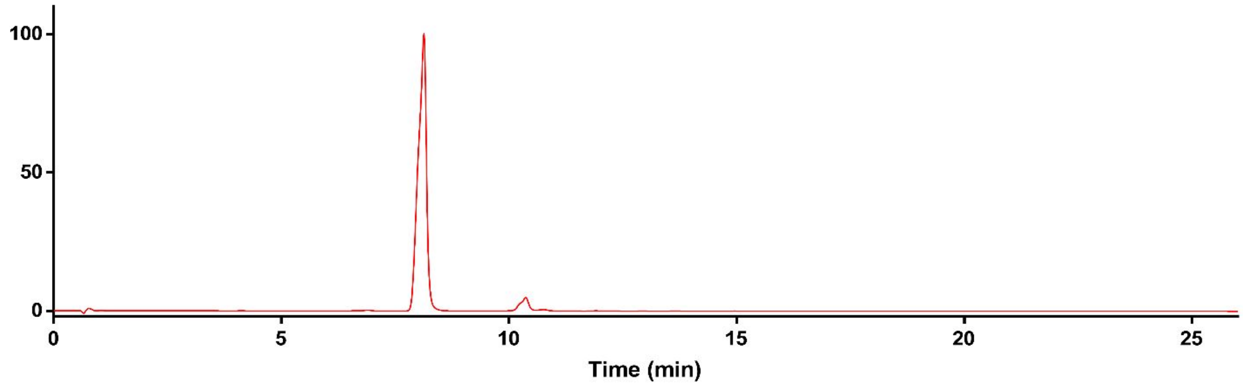

Figure S24: HPLC chromatogram of EtA–Dabcyl conjugate. The polarity of acetonitrile varied from 5 to 80% in 25 min and then upto 100% in 26 min. The conjugated product was isolated at retention time (R_t) 8.1 min.

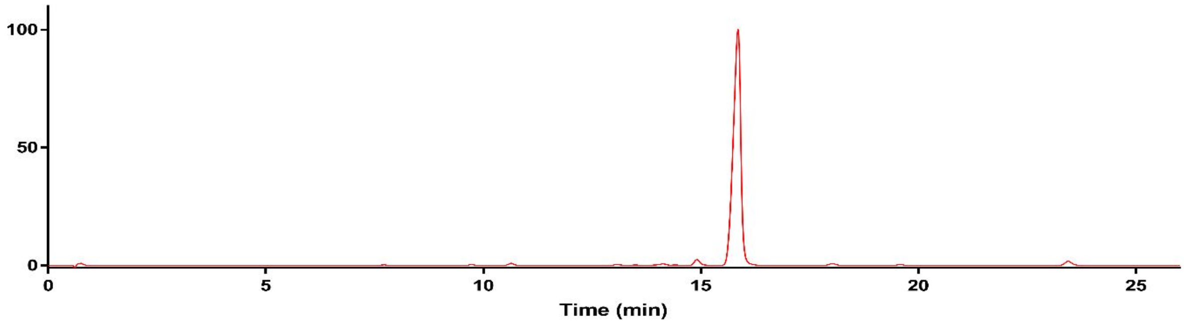

Figure S25: HPLC chromatogram of EtA–BHQ1 conjugate. The polarity of acetonitrile varied from 5 to 80% in 25 min and then upto 100% in 26 min. The conjugated product was isolated at retention time (R_t) 15.5 min.

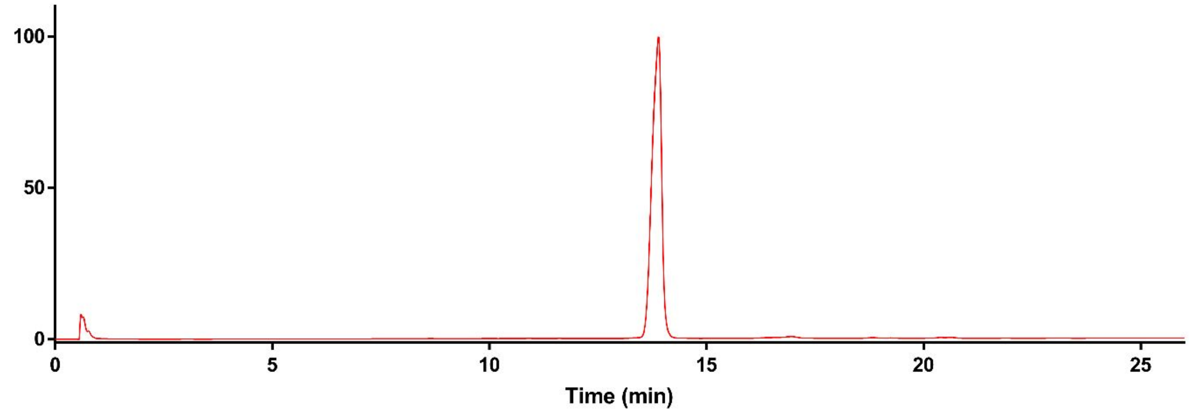

Figure S26: HPLC chromatogram of EtA–BHQ2 conjugate. The polarity of acetonitrile varied from 5 to 80% in 25 min and then upto 100% in 26 min. The conjugated product was isolated at retention time (R_t) 14.6 min.

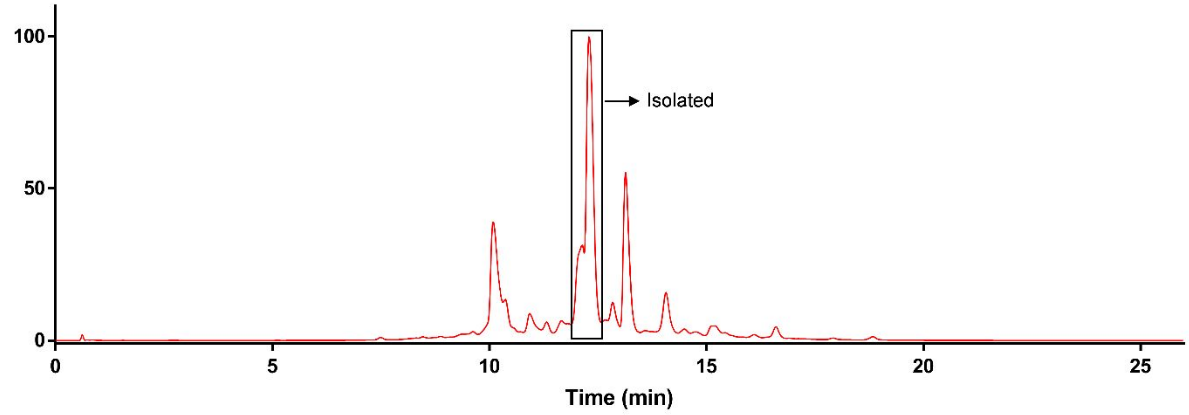

Figure S27: HPLC chromatogram of EtA–BHQ3 conjugate. The polarity of acetonitrile varied from 5 to 80% in 25 min and then upto 100% in 26 min. The conjugated product was isolated at retention time (R_t) 12.6 min.

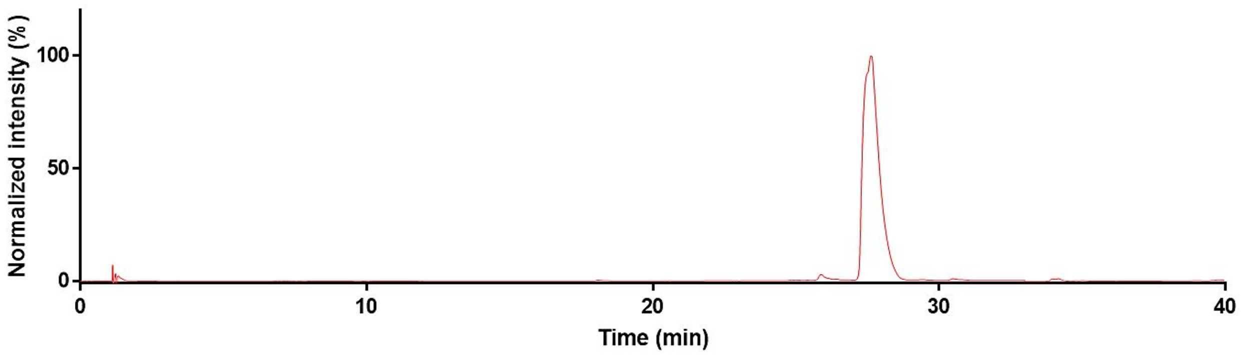

Figure S28: HPLC chromatogram of ADAPc–PEG–NHS ester. The polarity of acetonitrile varied from 5 to 100% in 40 min. The conjugated product was isolated at retention time (R_t) 28.5 min.

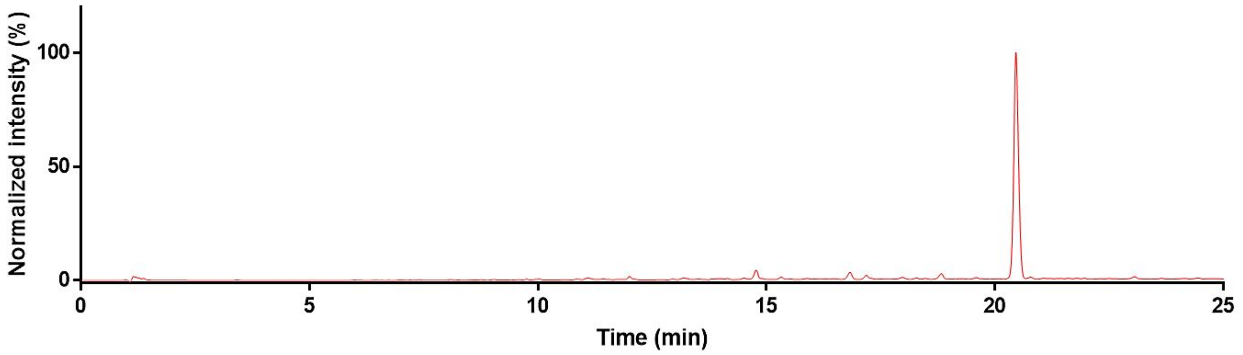

Figure S29: HPLC chromatogram of ADA conjugated taxol. The polarity of acetonitrile varied from 5 to 100% in 25 min. The conjugated product was isolated at retention time (R_t) 20.5 min.

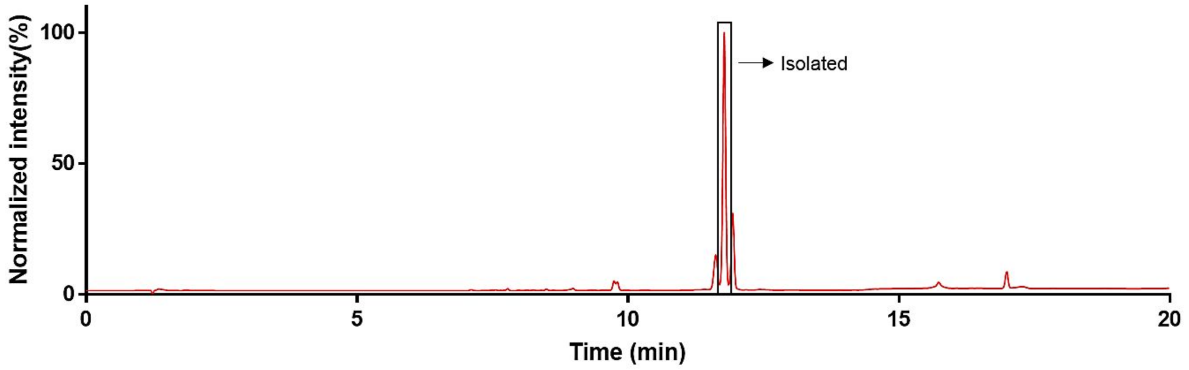

Figure S30: HPLC chromatogram of Phalloidin–TCO conjugate. The polarity of acetonitrile was changed from 5% to 50% in 15 min and then to 100% in 20 min. The conjugated product was isolated at retention time (R_t) 13.5 min.

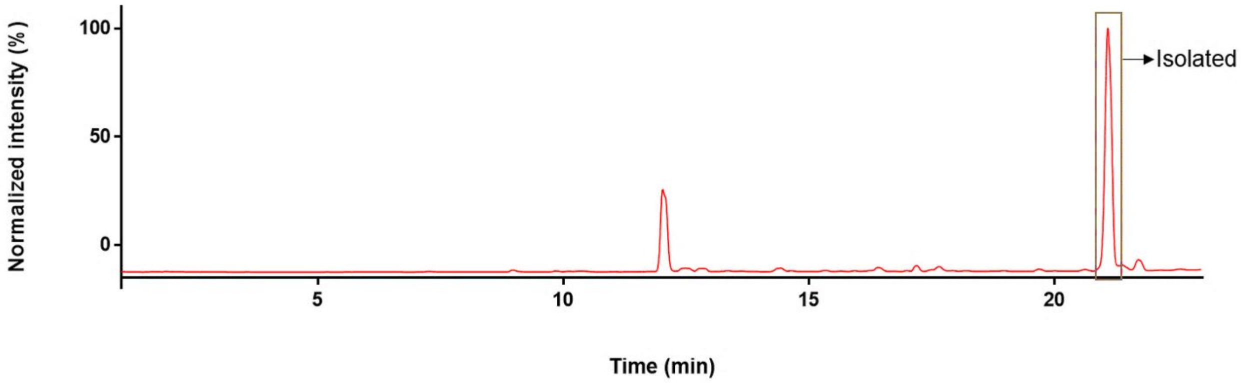

Figure S31: HPLC chromatogram of Jasplakinolide–TCO conjugate. The polarity of acetonitrile was changed from 5% to 70% in 20 min and then to 100% in 22 min.

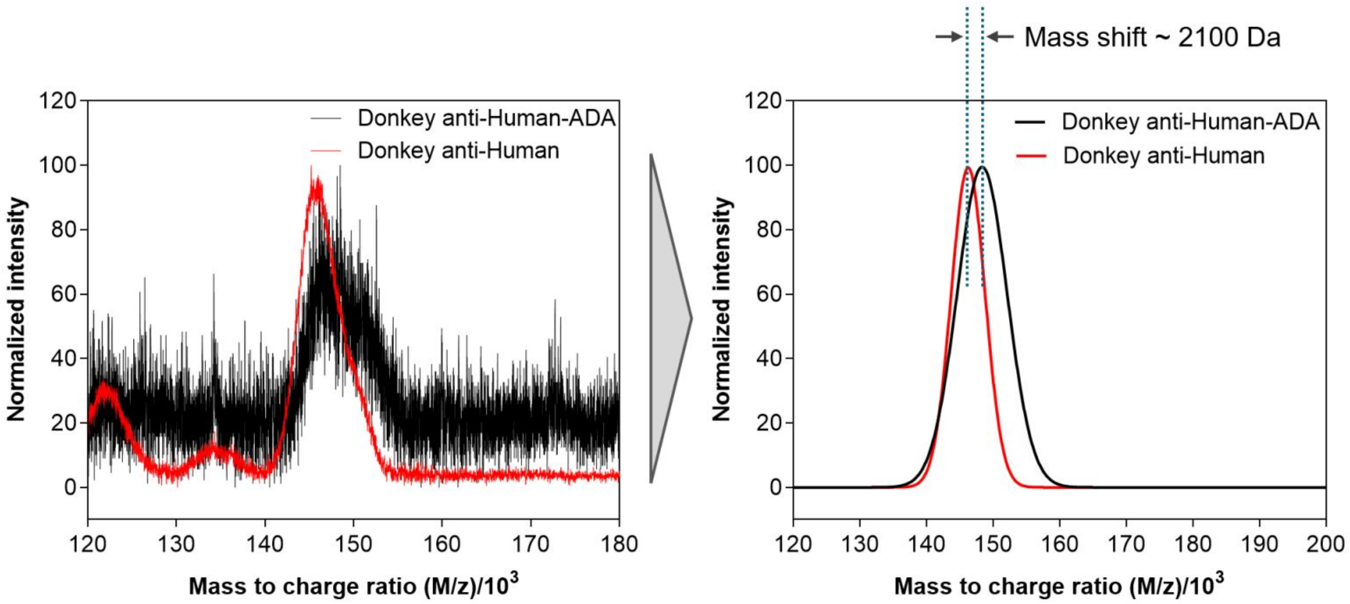

Figure S32: MALDI-MS analysis of ADA conjugated anti-human secondary antibody. The number of ADA moieties anchored to the antibodies was calculated to be three per anti-human antibody.

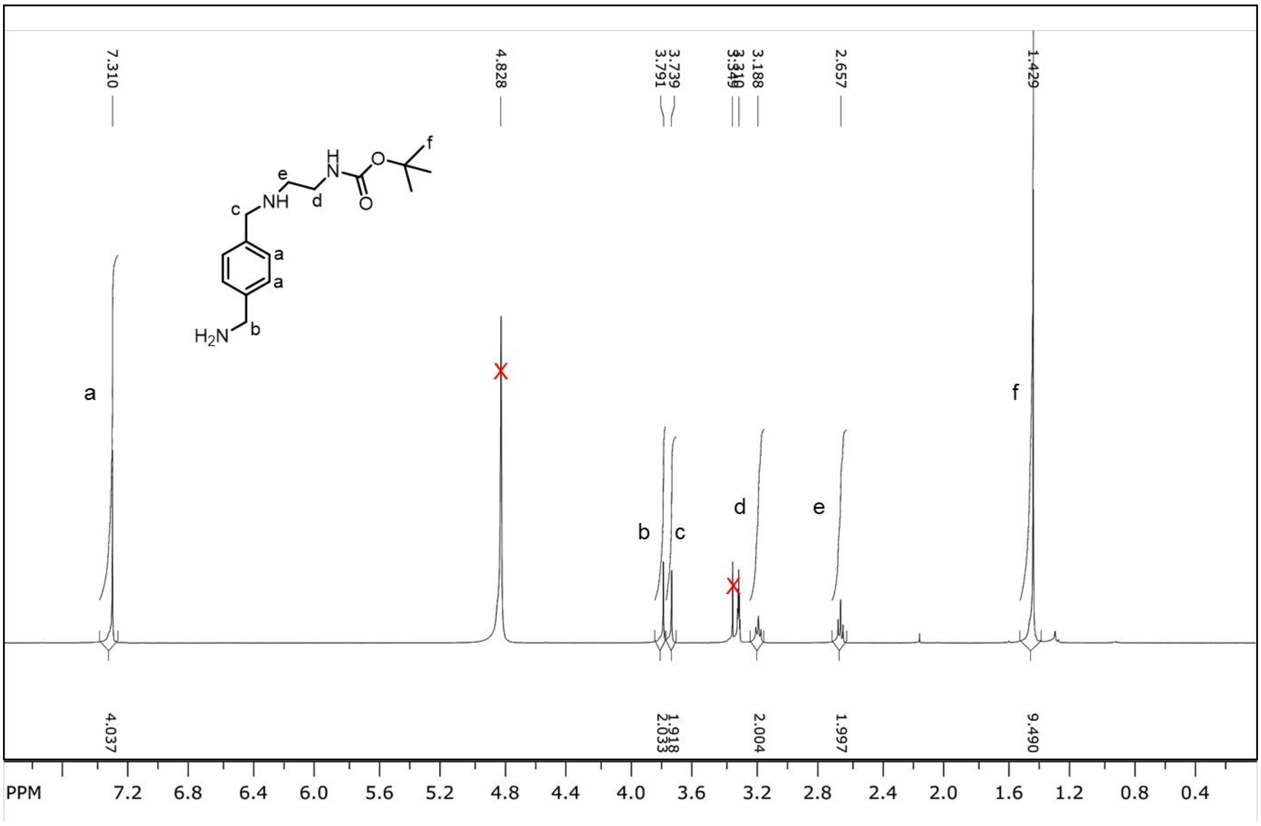

Figure S33: ^{1}H NMR of compound 2 (400 MHz, CD_3OD).

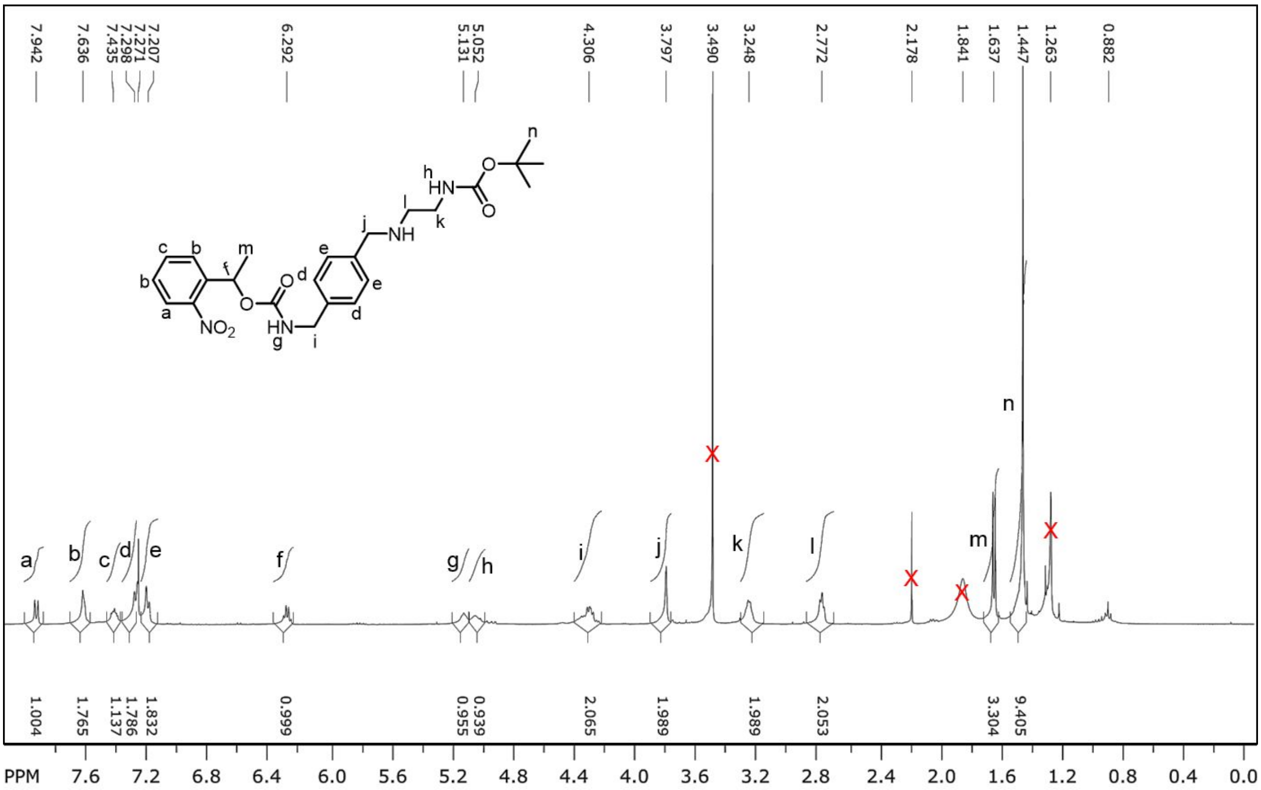

Figure S34: ^{1}H NMR of compound 4 (400 MHz, $CDCl_3$).

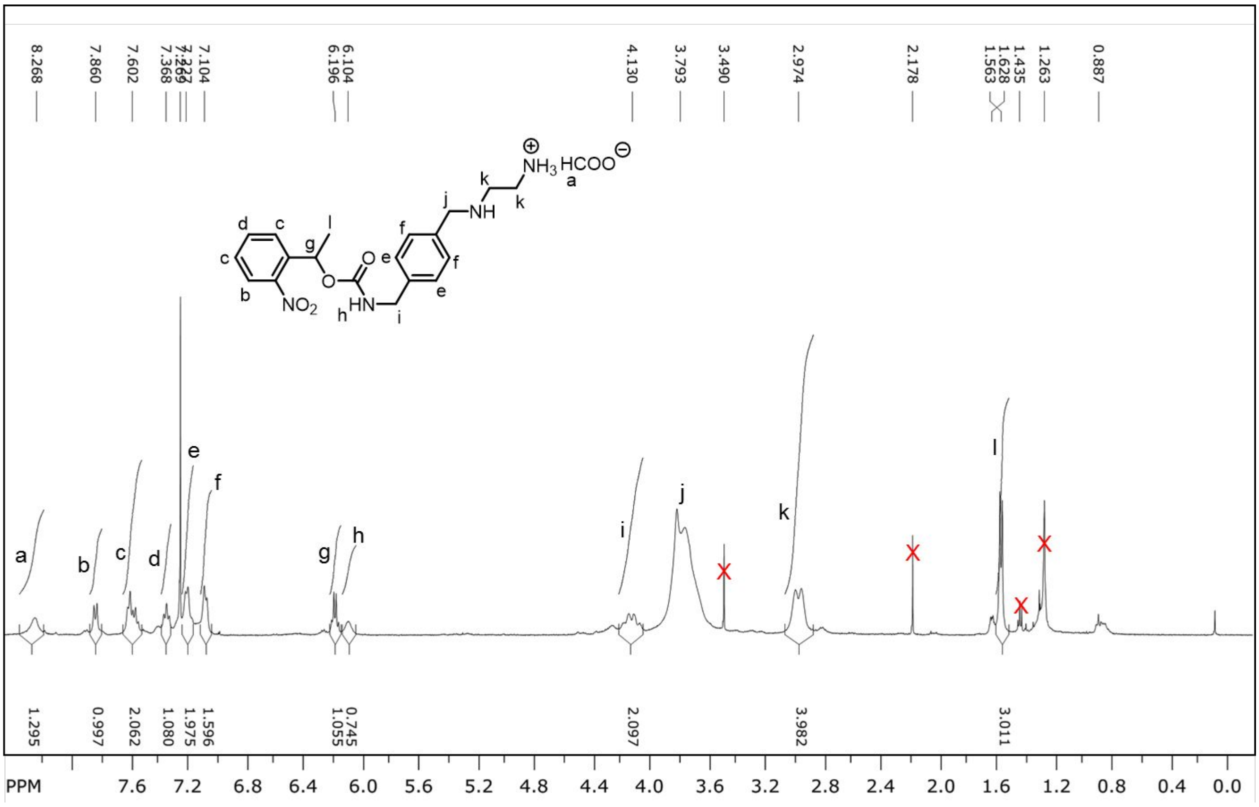

Figure S35: ^{1}H NMR of compound 5 (400 MHz, $CDCl_3$).

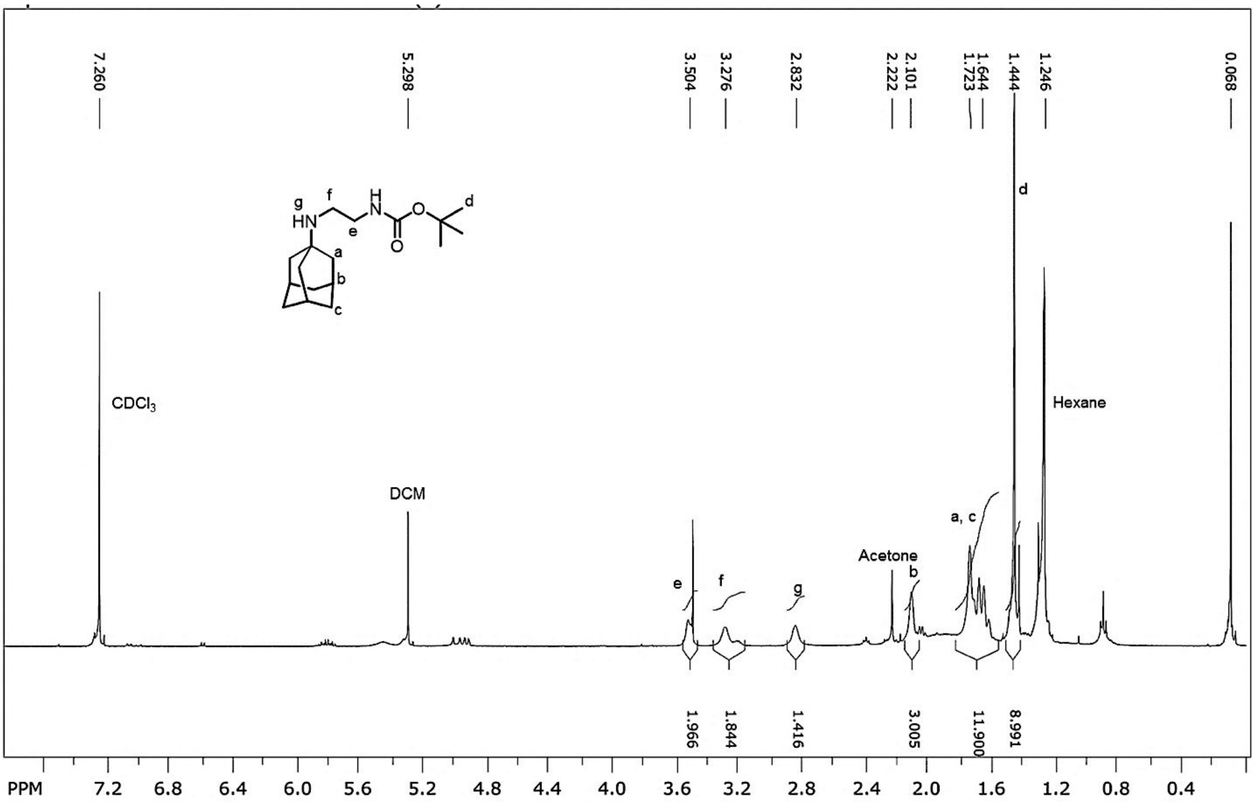

Figure S36: ^{1}H NMR of compound 6 (400 MHz, $CDCl_3$).

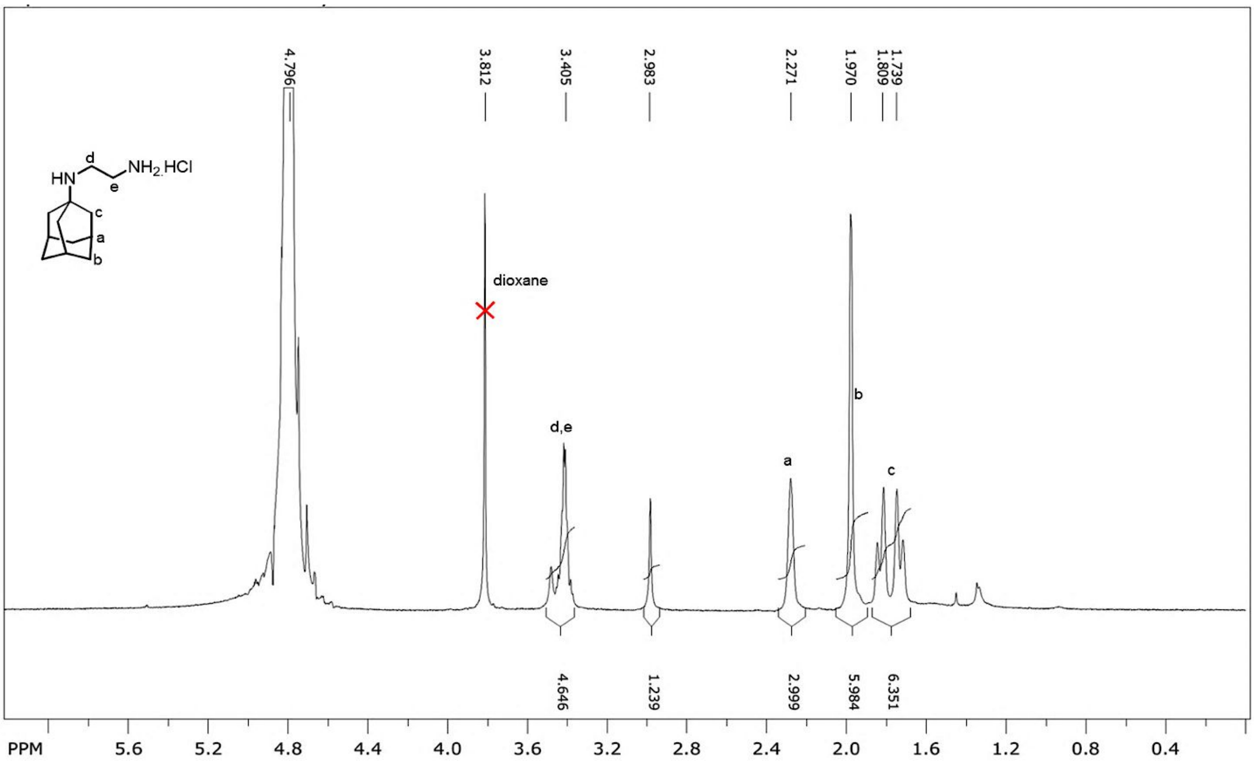

Figure S37: ^{1}H NMR of compound 7 (400 MHz, D_2O).

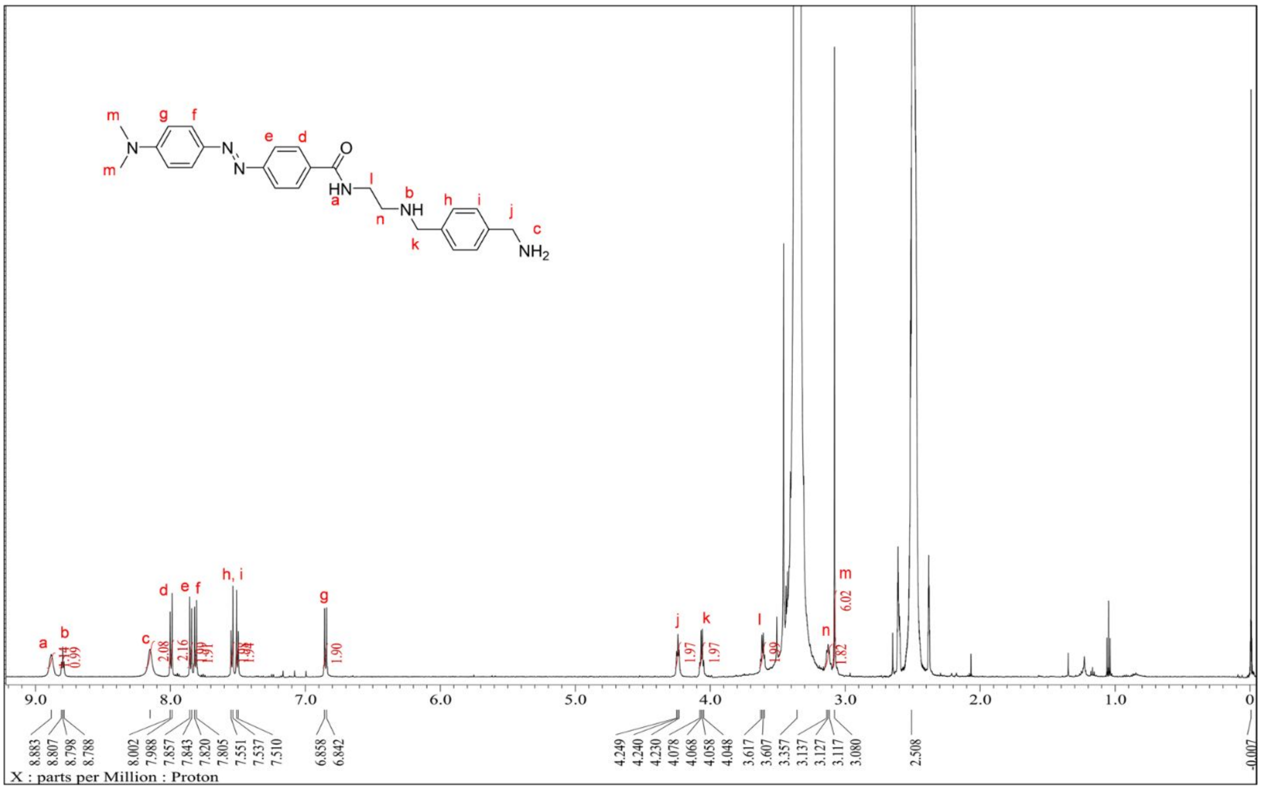

Figure S38: ^{1}H–NMR spectrum of XYL–Dabcyl conjugate (600 MHz, DMSO–d_6).

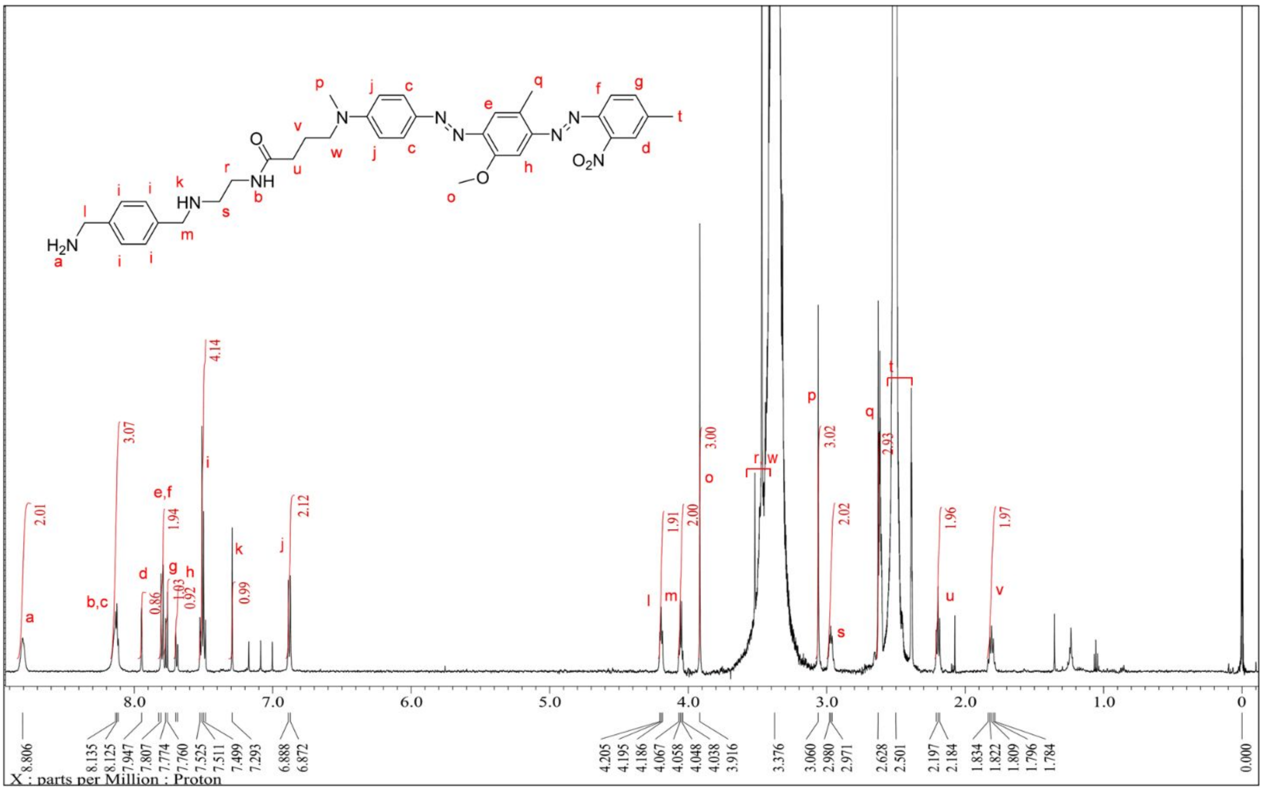

Figure S39: ^{1}H–NMR spectrum of XYL–BHQ1 conjugate (600 MHz, DMSO–d_6).

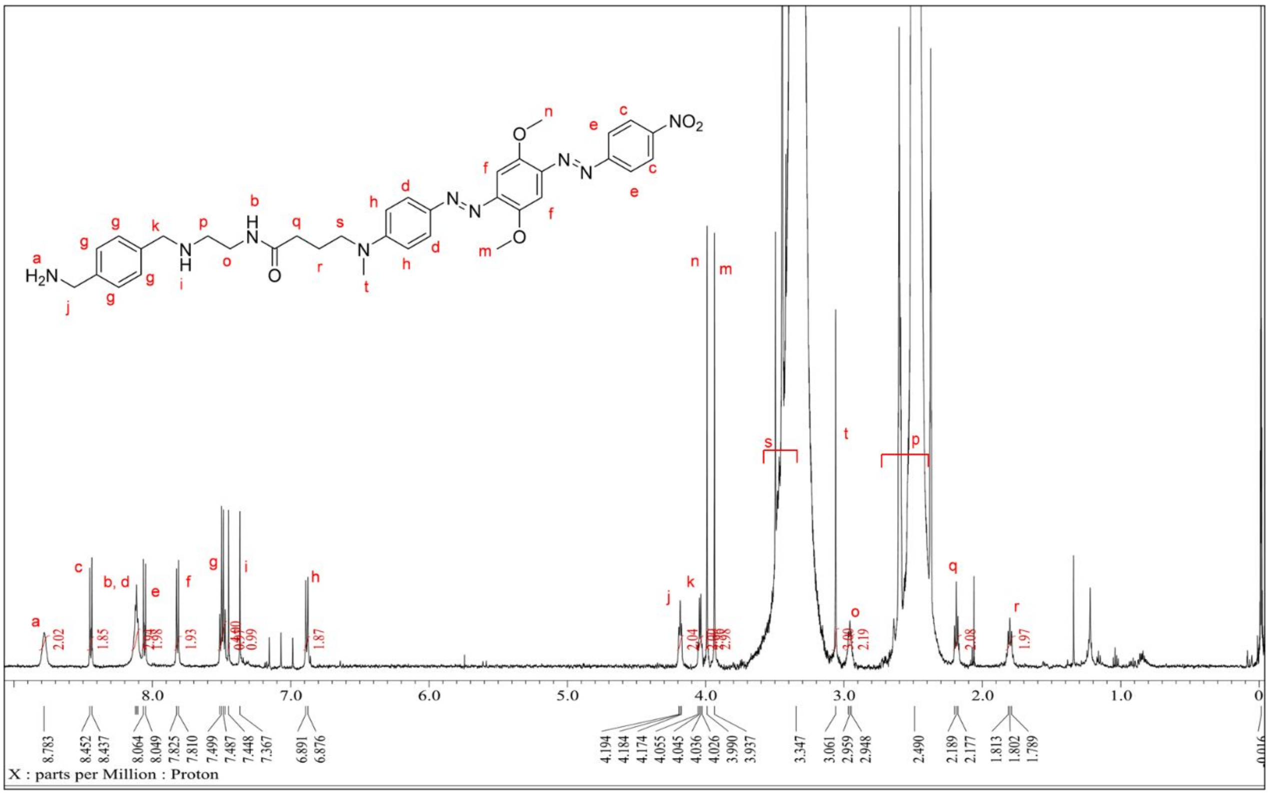

Figure S40: ^{1}H–NMR spectrum of XYL–BHQ2 conjugate (600 MHz, DMSO–d_6).

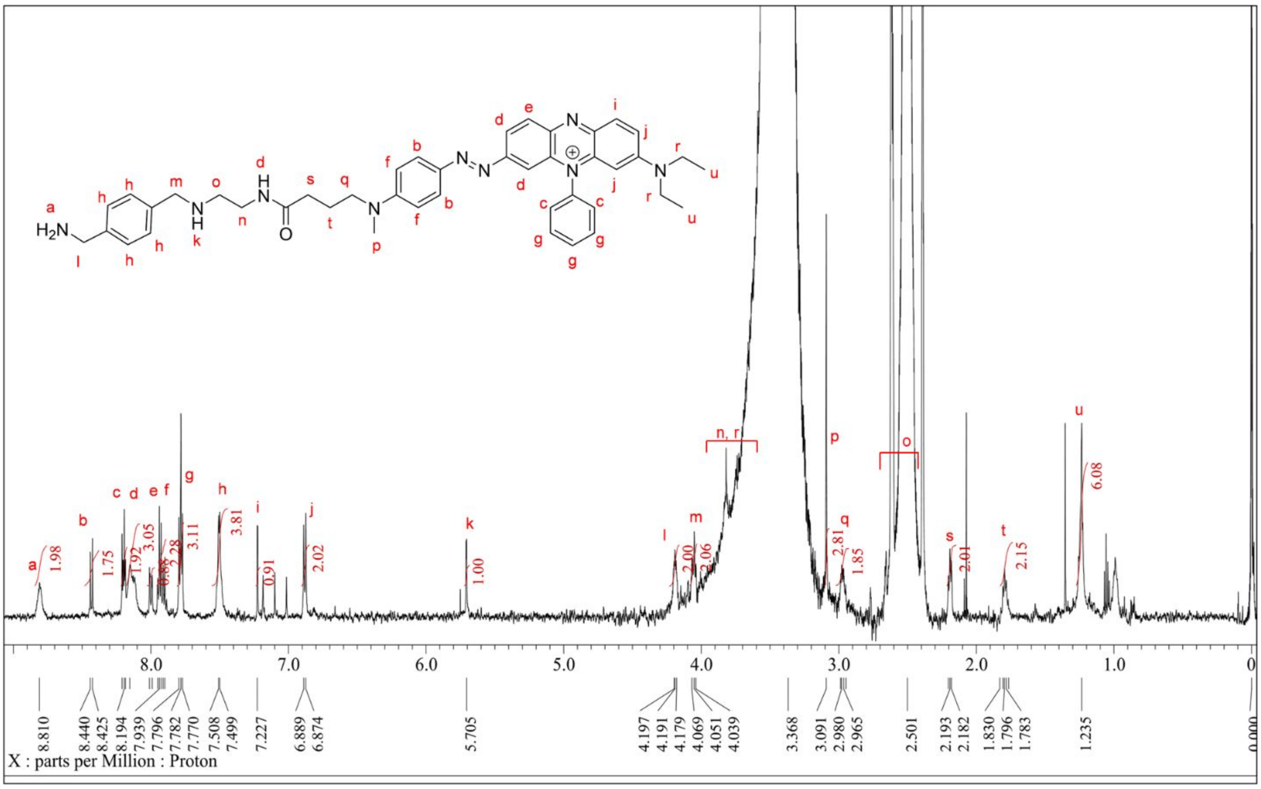

Figure S41: ^{1}H–NMR spectrum of XYL–BHQ3 conjugate (600 MHz, DMSO–d_6).

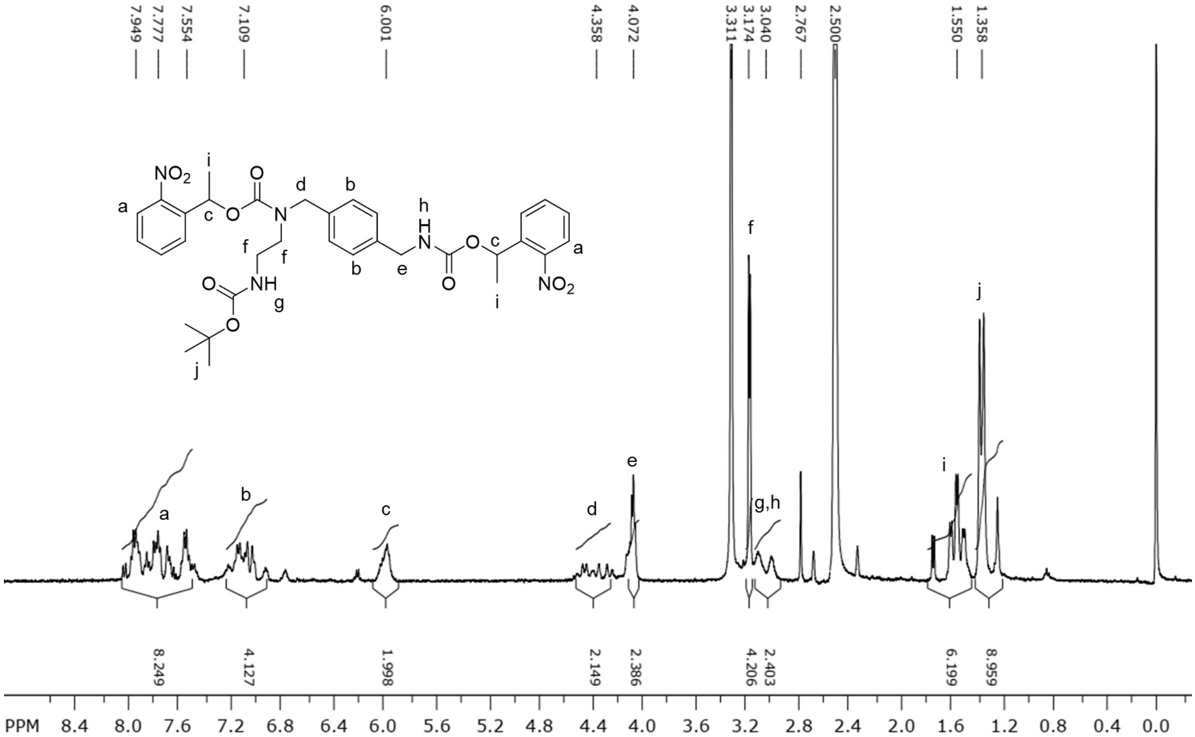

Figure S42: ^{1}H NMR spectrum of PC_2XYL-NHBoc in DMSO-d_6 (400 MHz).

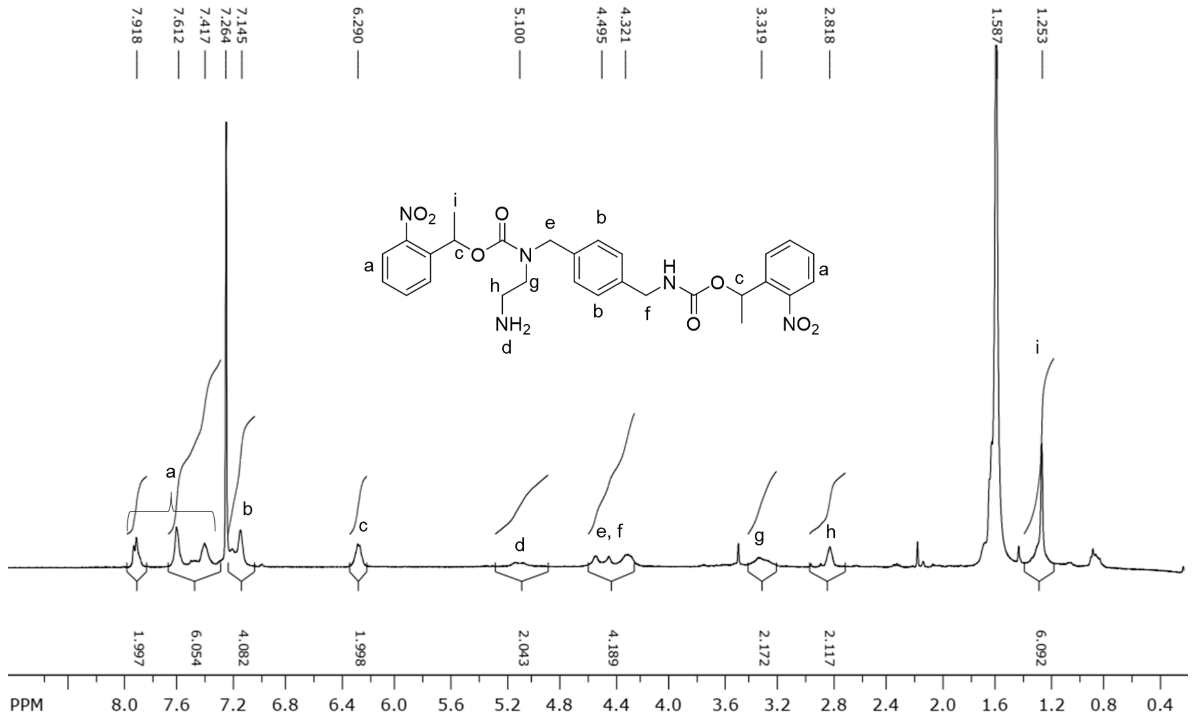

Figure S43. ^{1}H NMR spectrum of $PC_2XYL-NH_2$ in DMSO-d_6 (400 MHz).

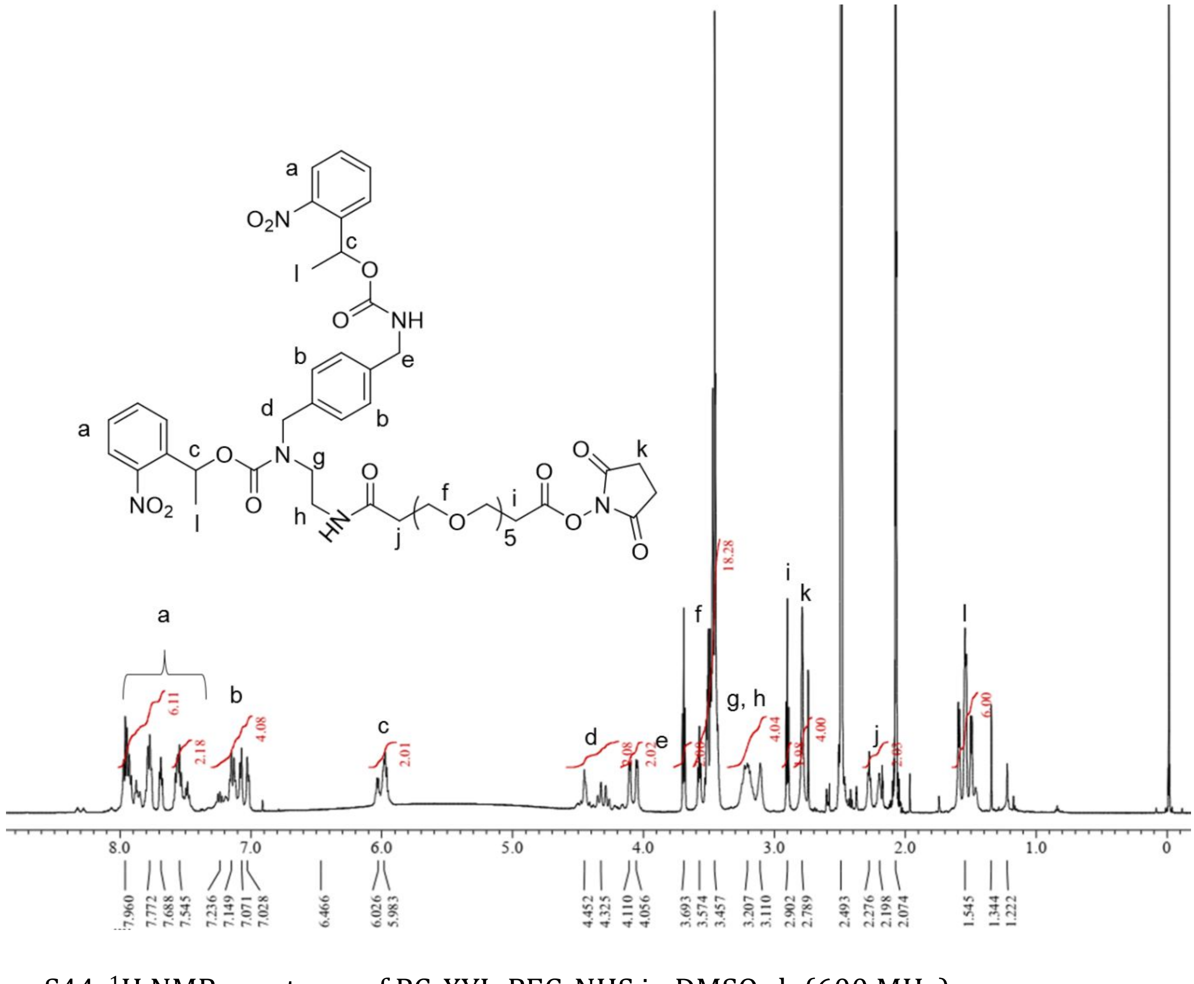

Figure S44. ^{1}H NMR spectrum of PC_2XYL-PEG-NHS in DMSO-d_6 (600 MHz).

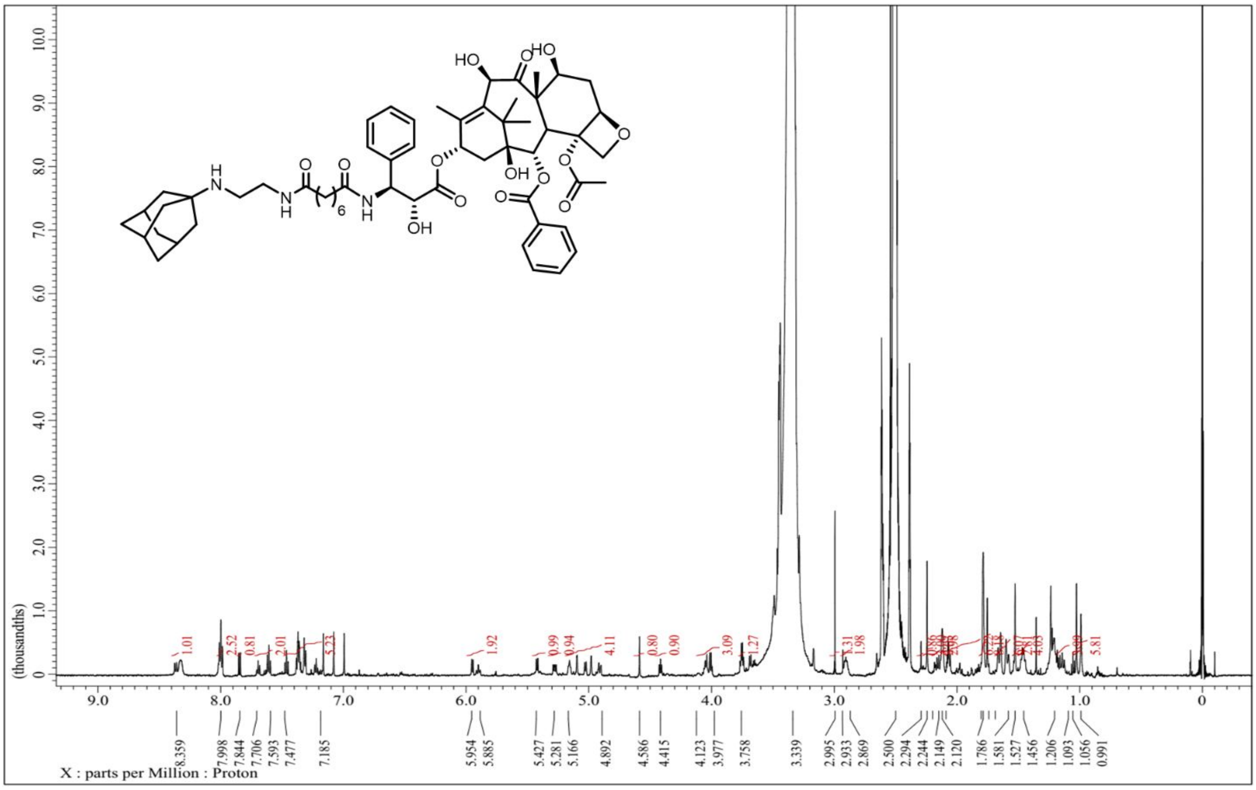

Figure S45: ^{1}H–NMR spectrum of ADA conjugated taxol (600 MHz, DMSO–d_6).

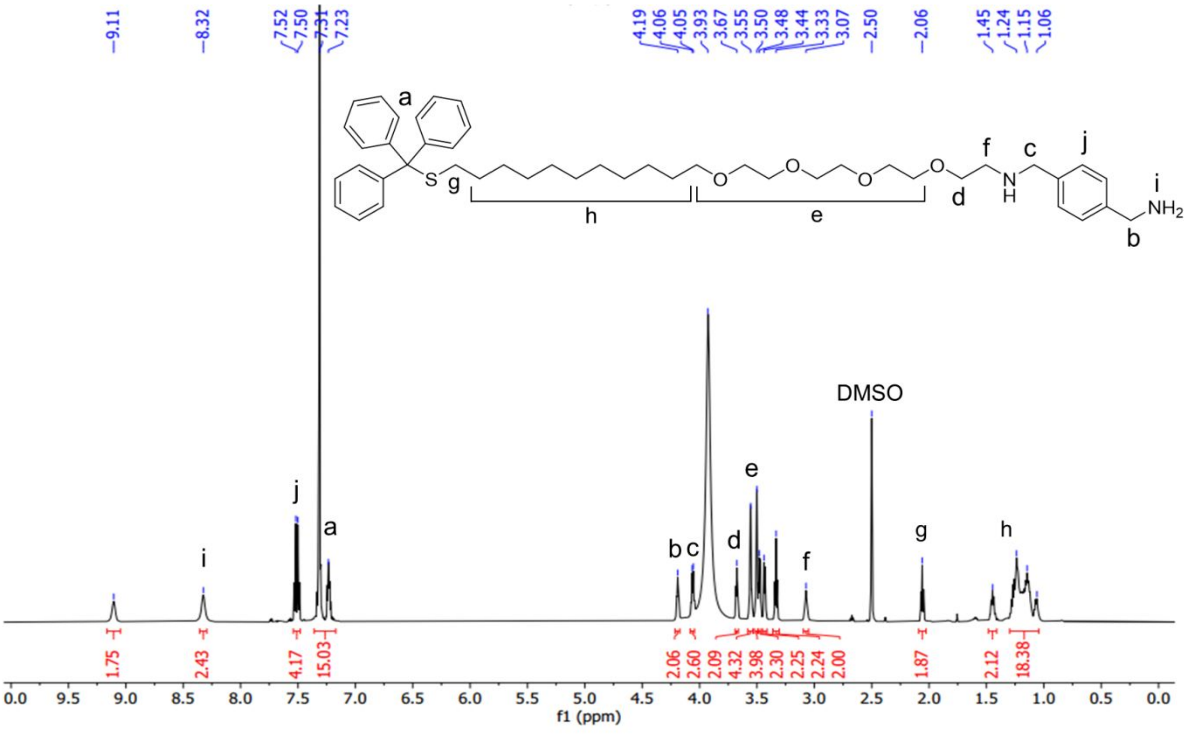

Figure S46: ^{1}H–NMR spectrum of Compound 17**:** Trt-C11-TEG-XYL (600 MHz, DMSO–d_6).

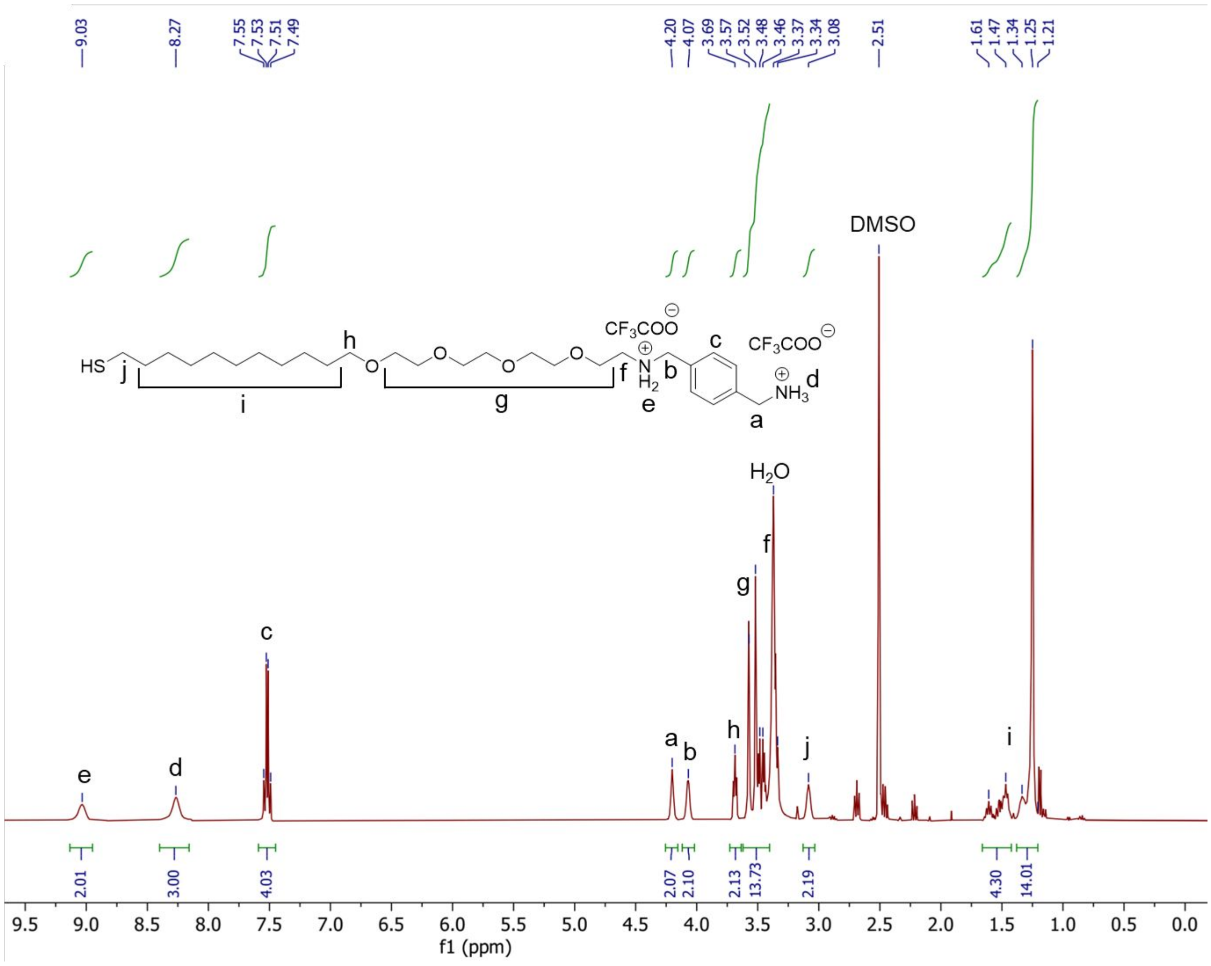

Figure S47: ^{1}H–NMR spectrum of Compound 18: HS-C11-TEG-XYL (400 MHz, DMSO–d_6).

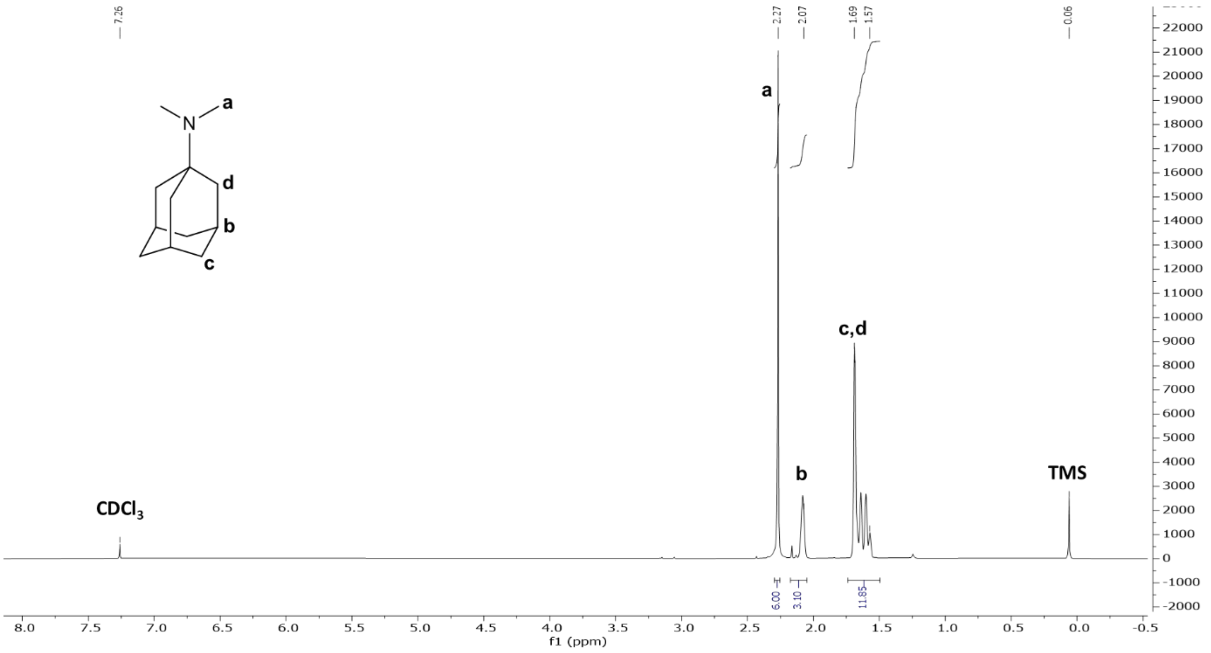

Figure S48: ^{1}H–NMR spectrum of ADA-NMe_2 (400 MHz, $CDCl_3$).

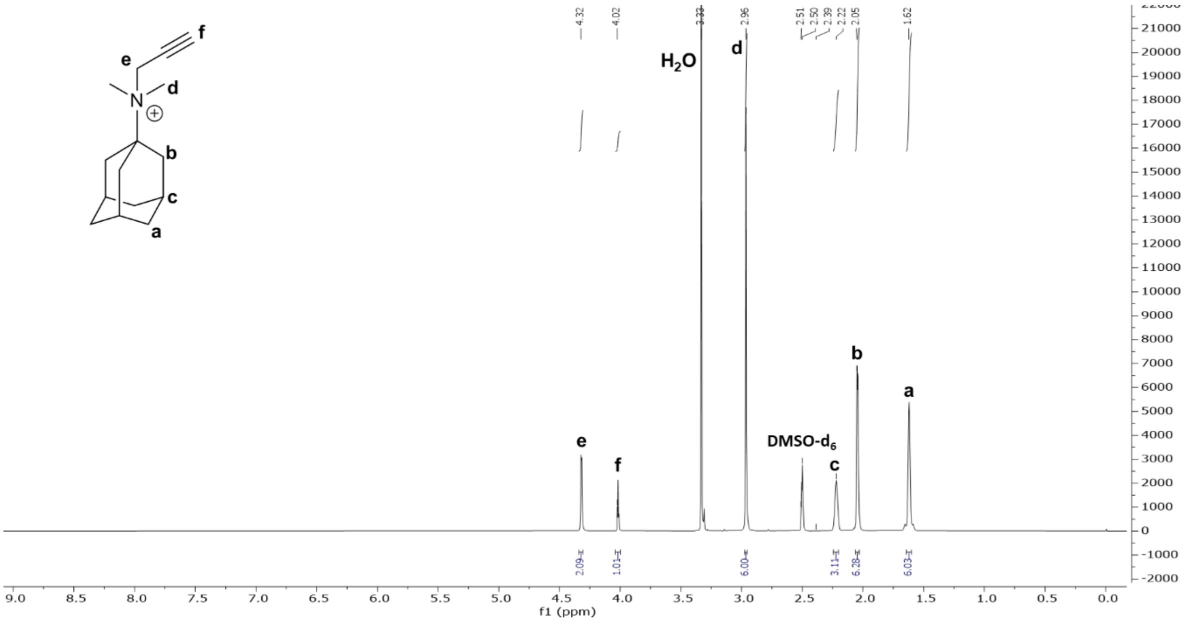

Figure S49: ^{1}H–NMR spectrum of ADA-NMe_2-propargyl (400 MHz, DMSO-d_6).

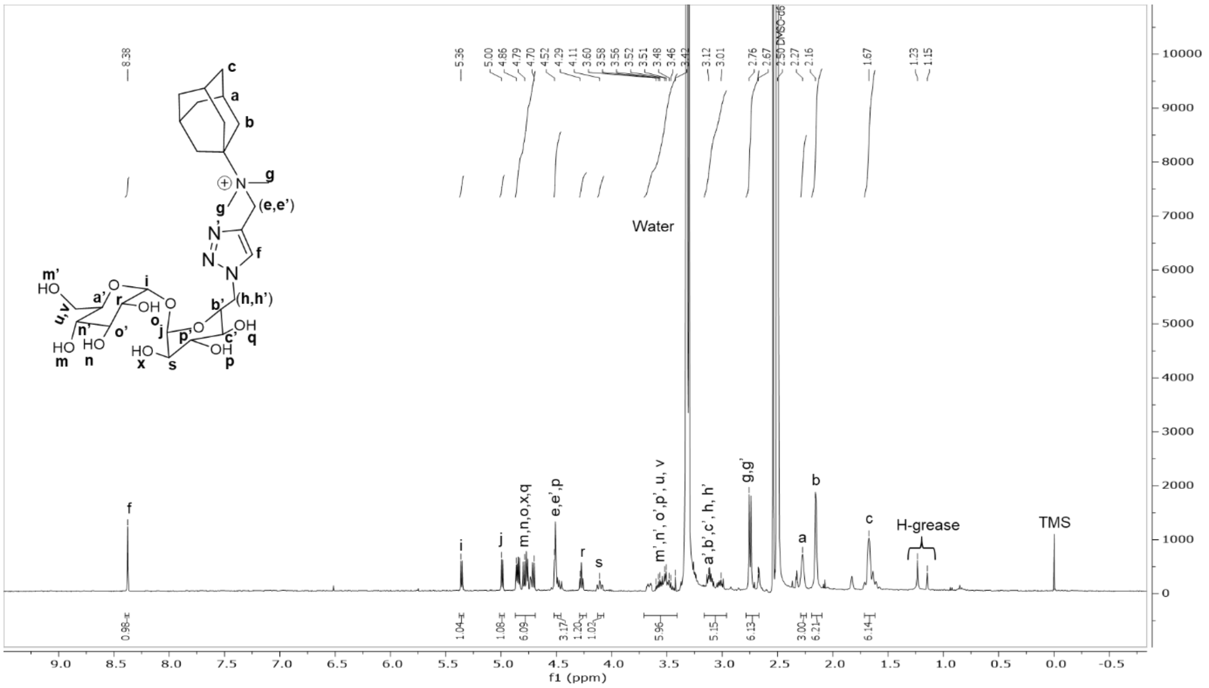

Figure S50:^{1}H–NMR spectrum of Trehalose-ADA (600 MHz, DMSO-d_6).

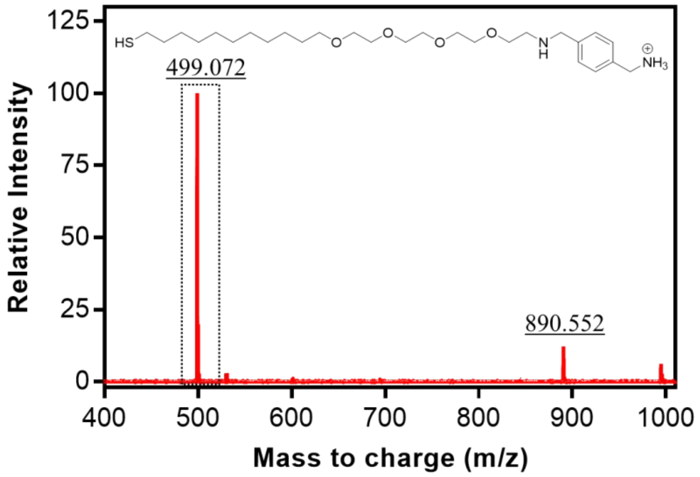

Figure S51: MALDI spectrum of XYL-AuNP showing a peak at m/z = 499.072 (observed) which corresponds to the calculated mass value (m/z = 499.356) of thiol ligand of xylene diamine moiety.

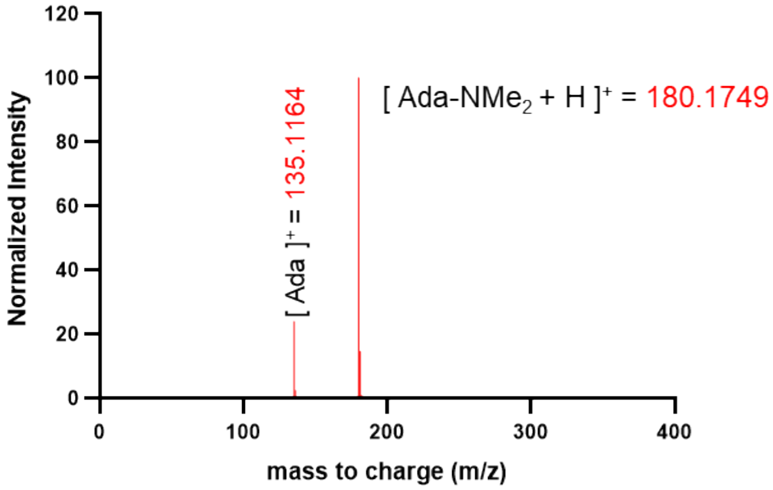

Figure S52: HRMS spectrum of Ada-NMe_2

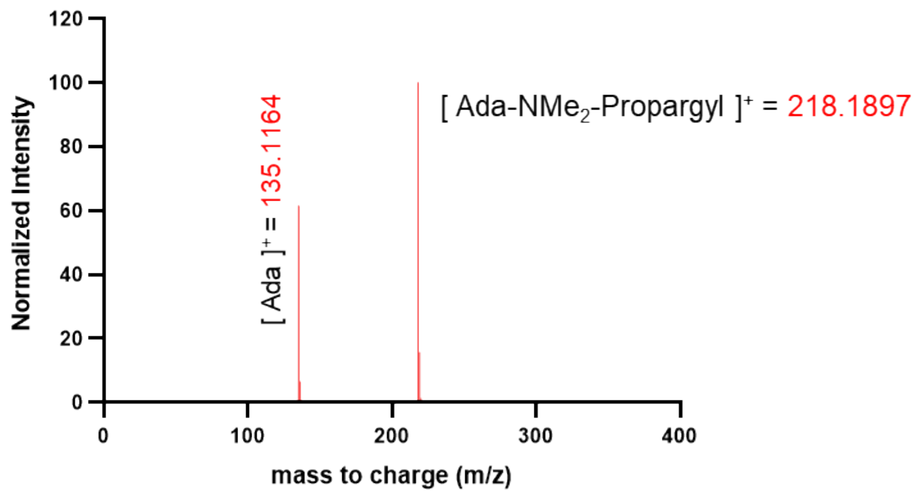

Figure S53: HRMS spectrum of Ada-NMe_2-Propargyl

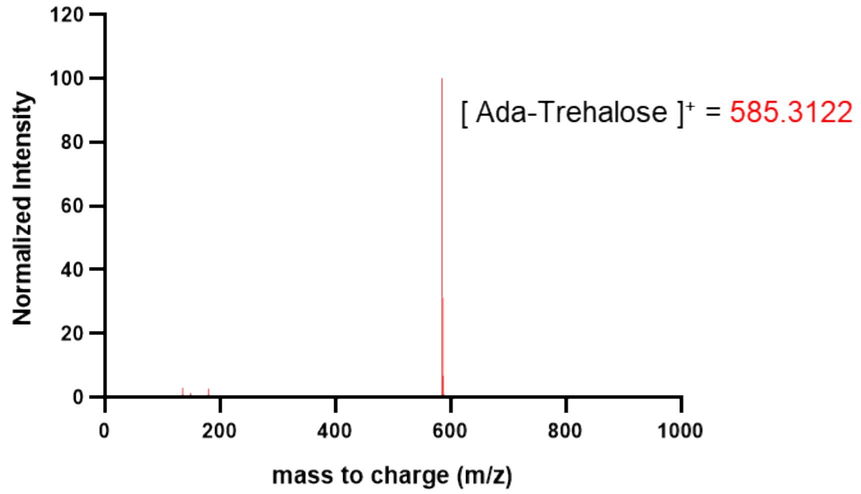

Figure S54: HRMS spectrum of Ada-Trehalose

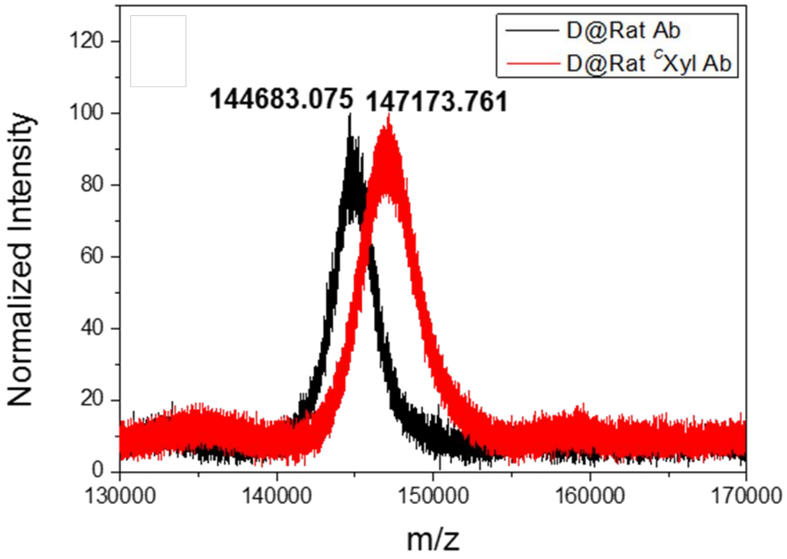

Figure S55. MALDI-MS spectra of PC_2XYL-PEG-secondary antibody (D@Rat-^{C}XYL Ab; Dockey anti-rat antibody). The spectrum in red is the modified antibody while the spectrum in black is the unmodified antibody.

Table S14: List of CB7 conjugated fluorophores that are used in this study[8]

Fluorophores	Synthesis references
CB7-Coumarin	7
CB7-Fluorescein	4
CB7-Bodipy	7
CB7-TAMRA	4
CB7-Cy3	4
CB7-Cy5	7
CB7-SiR	7

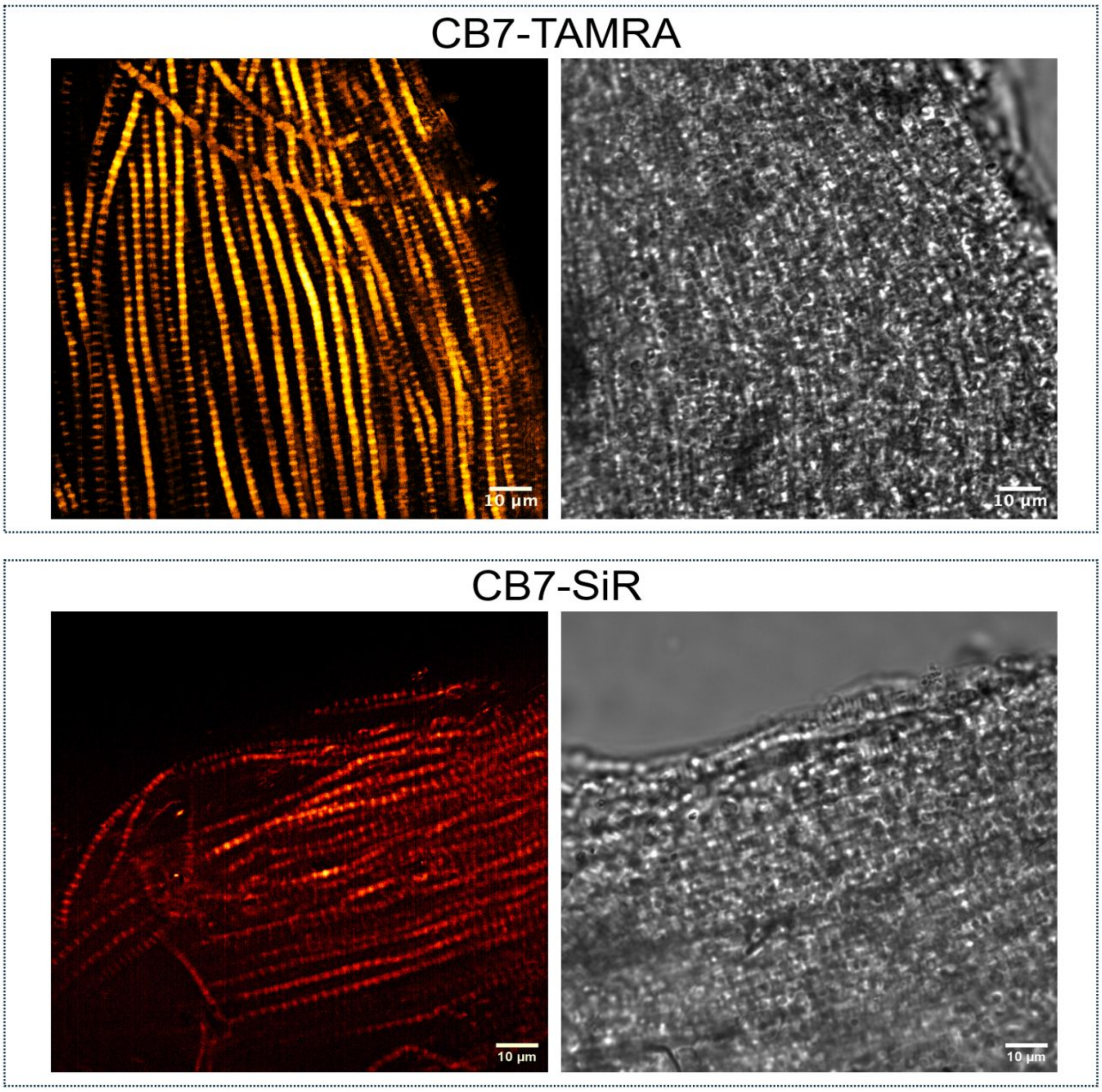

Figure S56. Fluorescence microscopy images along with corresponding bright-field images of the actin structures in fixed thoracic muscle tissue of *Drosophila melanogaster*.

14. References

1. K. Chauhan, A. Datta, A. Adhikari, K. Chuttani, A. K. Singh and A. K. Mishra, ^{68}Ga based probe for Alzheimer's disease: synthesis and preclinical evaluation of homodimeric chalcone in β-amyloid imaging. *Org. Biomol. Chem.*, **2014**, 12, 7328–7337.

2. Y. Kikuchi, J. Nakanishi, T. Shimizu, H. Nakayama, S. Inoue, K. Yamaguchi, H. Iwai, Y. Yoshida, Y. Horiike, T. Takarada and M. Maeda, Arraying Heterotypic Single Cells on Photoactivatable Cell-Culturing Substrates. *Langmuir*, **2008**, 24, 13084–13095.

3. N. Devaraj, S. Hilderbrand, R. Upadhyay, R. Mazitschek and R. Weissleder, Bioorthogonal turn-on probes for imaging small molecules inside living cells. *Angew. Chem., Int. Ed.*, **2010**, 49, 2869 –2872.

4. Som, A.; Pahwa, M.; Bawari, S.; Saha, N. D.; Sasmal, R.; Bosco, M. S.; Mondal, J.; Agasti, S. S., Multiplexed optical barcoding of cells via photochemical programming of bioorthogonal host-guest recognition. *Chem. Sci.* **2021,** *12*, 5484-5494.

5. Miranda, O. R.; Chen, H.-T.; You, C.-C.; Mortenson, D. E.; Yang, X.-C.; Bunz, U. H. F.; Rotello, V. M. Enzyme-Amplified Array Sensing of Proteins in Solution and in Biofluids. *J. Am. Chem. Soc.* **2010**, *132*, 5285–5289.

6. Kulathinte Meethal, S.; Sasmal, R.; Pahwa, M.; C., S.; Das Saha, N.; Agasti, S. S. Cucurbit[7]uril-Directed Assembly of Colloidal Membrane and Stimuli-Responsive Microcapsules at the Liquid–Liquid Interface. *Langmuir* **2018**, *34*, 693–699.

7. Cosma, C. L.; Klein, K.; Kim, R.; Beery, D.; Ramakrishnan, L. Mycobacterium Marinum Erp Is a Virulence Determinant Required for Cell Wall Integrity and Intracellular Survival. *Infection and Immunity* **2006**, *74 (6)*, 3125–3133.

8. Das Saha, N.; Pradhan, S.; Sasmal, R.; Sarkar, A.; Berač, C. M.; Kölsch, J. C.; Pahwa, M.; Show, S.; Rozenholc, Y.; Topçu, Z.; Alessandrini, V.; Guibourdenche, J.; Tsatsaris, V.; Gagey-Eilstein, N.; Agasti, S. S. Cucurbit[7]uril Macrocyclic Sensors for Optical Fingerprinting: Predicting Protein Structural Changes to Identifying Disease-Specific Amyloid Assemblies. *J. Am. Chem. Soc.* **2022**, *144*, 14363-14379.
