# Supplementary material for: Supramolecular Guest Exchange in Cucurbit[7]uril for Bioorthogonal Fluorogenic Imaging across the Visible Spectrum
Source: ACS Cent Sci. 2024 Oct 8;10(10):1945–59. doi: 10.1021/acscentsci.4c01080 (PMC11503495; doi:10.1021/acscentsci.4c01080)
Supplement: Supplementary file 2 — oc4c01080_si_002.pdf [file oc4c01080_si_002.pdf]

oc-2024-01080q.R1

Name: Peer Review Information for "Supramolecular Guest Exchange in Cucurbit[7]uril For Bioorthogonal Fluorogenic Imaging Across the Visible Spectrum"

First Round of Reviewer Comments

Reviewer: 1

Comments to the Author

The manuscript by Sasmal et al. presents an innovative approach to biocompatible fluorogenic labeling. This method is based on displacing a quencher-modified guest molecule from its complex with fluorophore-conjugated cucurbit[7]uril (CB7). The elegance of the system lies in its ability to quench various fluorophores using different quenchers. The applicability of this methodology is convincingly demonstrated through several examples, including intracellular labeling, which is notably achieved by exploiting the transport and quenching capabilities of Au nanoparticles. Additionally, the authors demonstrate the orthogonality of their method to Tz/TCO bioorthogonal labeling.

Given the growing interest in host-guest systems within the field of biology, the results presented in this study are likely to attract broad readership. Although other CB7-based approaches have been documented in the literature (which are appropriately cited in the manuscript), I am unaware of any prior use of the specific principle described here. This contributes significantly to the novelty of the work.

Overall, I find this technology to be highly promising for various imaging applications. Considering that the article has already undergone peer review, I did not identify any major flaws or inconsistencies. Furthermore, I do not believe that additional experiments are necessary to emphasize the potential applications of this approach, as the new experiments included in the latest revision sufficiently achieve this.

Therefore, I recommend this work for publication after minor revisions.

1) Cit. 21, there are a few more examples of bioorthogonal reactions that lead to formation of dyes during the reaction. I encourage the authors to cite a few more. For example:

https://onlinelibrary.wiley.com/doi/epdf/10.1002/anie.200705805?src=getftr

https://onlinelibrary.wiley.com/doi/full/10.1002/anie.201610491

https://onlinelibrary.wiley.com/doi/10.1002/anie.202011544

https://onlinelibrary.wiley.com/doi/10.1002/anie.201205352

https://chemistry-europe.onlinelibrary.wiley.com/doi/full/10.1002/cplu.201900176

2) Cit. 43. I am aware of at least this work which uses the same quencher/fluorophore principle (could be also cited).

https://chemistry-europe.onlinelibrary.wiley.com/doi/full/10.1002/chem.201905446

This fluorogenic reaction is based on the Tz/TCO chemistry, which does not have sluggish kinetics. The sentence describing previous work along these lines (page 2, raw 7, right column) should be changed accordingly.

3) Based on the fluorogenic data presented in Figure 2A, is it possible to quantify the fluorescence turn-on values (e.g. from integrated signal area)? I couldn’t find this information in the manuscript but including it could be valuable for the scientific community. It would enable comparisons with other relevant systems.

4) Could the authors specify which TCO derivative they used in the synthesis of phalloidin and jasplakinolide probes? The axial isomer, equatorial isomer or mixture?

Reviewer: 2

Comments to the Author

The authors present a supramolecular fluorogenic imaging strategy that could be used to complement existing covalent strategies for fluorogenic, i.e. reactive turn-ON targeting, probes. The manuscript conceptualizes the strategy using host-guest displacement to achieve the goal, which is - to the best of my knowledge - conceptually new.

The strategy appears to be widely adaptable to different fluorescent dye-quencher pairs that are established in fluorescence imaging and to different biological assay methods, and the present manuscript is an excellent proof-of-concept.

To further improve the quality of manuscript, I recommend to elaborate in the manuscript on the advantages of the supramolecular method compared to covalent fluorogenic labelling methods. The advantages of the supramolecular method are not sufficiently pin-pointed yet. For example, it

would be beneficial to mention the rapid response times (Fig. 2b), which is highly beneficial, for example, for pulse-chase assays. Perhaps, it would be even possible to roughly assess the kinetics of the supramolecular exchange and compare it with well-established covalent methods.

Also the potential advantages to target small, minimalistic labels with the supramolecular strategy should be compared to existing methods using streptavidin-biotin or covalent fluorogenic probes. Conceivable issues include the limited cell permeability of streptavidin for intracellular imaging or the potential instability of common covalent labels compared to an adamantane label.

References that may be useful to the authors to address this request are listed below:

Nat. Rev. Chem. 2019, 3, 393–400. https://doi.org/10.1038/s41570-019-0095-1

Angew. Chem. Int. Ed. 2023, 62, e202314373. https://doi.org/10.1002/anie.202314373

Nat. Commun. 2024, 15, 1851. https://doi.org/10.1038/s41467-024-46034-z

ACS Med. Chem. Lett. 2011, 2 (12), 885–889. https://doi.org/10.1021/ml200187j

Chem. Commun. 2024, 60, 4810-4813. https://doi.org/10.1039/D4CC00602J

A further minor issue is found on p.10, left column, lines 3-5, where authors state that "excellent quenching of CB7-TAMRA fluorescence upon addition of 0.2 equivalent of XYL-AuNP", which does not match the data in the inset of Fig. 6c.

After addressing these issues, the manuscript is well-suited for publication in ACS Central Science.

Author's Response to Peer Review Comments:

Formatting Needs:

Comment 1: ABSTRACT WORD COUNT: Please make sure the word count does not exceed 200 words.

Ans. The abstract has been shortened to comply with the word limit.

Comment 2: SYNOPSIS MISSING: The synopsis should be no more than 200 characters (including spaces) and should reasonably correlate with the TOC graphic. The synopsis is intended to explain the importance of the article to a broader readership across the sciences. Please place your synopsis in the manuscript file after the TOC graphic, and label it as "Synopsis."

Ans. A synopsis has been added at the end of the manuscript file after the TOC graphic.

Comment 3: TOC MISSING: Provide a TOC image per journal guidelines (3.25 in. × 1.75 in. (8.25 cm × 4.45 cm); on the last page of the Manuscript) with the heading "TOC Graphic" above the graphic. Make sure to designate the file as "Graphic for Manuscript."

Ans. The TOC has been included both on the last page of the manuscript and provided separately as a file titled "Graphic for Manuscript."

Reviewer 1:

We thank the reviewer for evaluating our work and appreciate the positive feedback regarding the manuscript.

Comment 1: Cit. 21, there are a few more examples of bioorthogonal reactions that lead to formation of dyes during the reaction. I encourage the authors to cite a few more. For example:
https://onlinelibrary.wiley.com/doi/epdf/10.1002/anie.200705805?src=getftr
https://onlinelibrary.wiley.com/doi/full/10.1002/anie.201610491
https://onlinelibrary.wiley.com/doi/10.1002/anie.202011544
https://onlinelibrary.wiley.com/doi/10.1002/anie.201205352
https://chemistry-europe.onlinelibrary.wiley.com/doi/full/10.1002/cplu.201900176

Ans. Thank you for the valuable feedback and suggestions. We appreciate your recommendation to include more examples of bioorthogonal reactions that lead to the formation of dyes. We have added the suggested references to the manuscript along with the Ref. 21 (22-26).

Comment 2: Cit. 43. I am aware of at least this work which uses the same quencher/fluorophore principle (could be also cited).
https://chemistry-europe.onlinelibrary.wiley.com/doi/full/10.1002/chem.201905446
This fluorogenic reaction is based on the Tz/TCO chemistry, which does not have sluggish kinetics. The sentence describing previous work along these lines (page 2, raw 7, right column) should be changed accordingly.

Ans. We sincerely thank the reviewer for bringing to our attention the relevant references regarding the use of bioorthogonal reactions with fluorophore-quencher pairs. We have now included the suggested reference (Ref. 49) in our manuscript to properly acknowledge this important work.
We agree that Tz-TCO chemistry offers a highly effective fluorogenic tool with faster kinetics. However, to the best of our knowledge, this approach has not yet been applied to the fluorogenic labeling of biomolecules within cellular environments. This distinction sets the phosphene probe apart from the Tz-TCO probe, particularly in terms of its application in cellular contexts. Therefore, we believe that the point of sluggish kinetics remains appropriate within the scope of our discussion and aligns with the goal of our manuscript.

Comment 3: Based on the fluorogenic data presented in Figure 2A, is it possible to quantify the fluorescence turn-on values (e.g. from integrated signal area)? I couldn't find this information in the manuscript but including it could be valuable for the scientific community. It would enable comparisons with other relevant systems.

Ans. Thank you for the suggestion. We have now calculated the fluorescence turn-on values for each fluorophore based on the fluorescence recovery data. These results have been included in Supporting Information Figure S3 and are also referenced in the main text.

Comment 4: Could the authors specify which TCO derivative they used in the synthesis of phalloidin and jasplakinolide probes? The axial isomer, equatorial isomer or mixture?

Ans. We used two types of TCO derivatives: TCO-NHS (jasplakinolide) and TCO-PEG-NHS (phalloidin), both obtained from Click Chemistry Tools. At the time of procurement, the commercial supplier did not provide any details on the isomeric composition of these TCO derivatives. To ensure clarity for the reader regarding the exact compounds used in this study, we have included the name of the commercial supplier and the exact catalog numbers of the compounds used.

Reviewer 2:

We thank the reviewer for evaluating our work and appreciate the positive feedback regarding the manuscript.

Comment 1: To further improve the quality of manuscript, I recommend toelaborate in the manuscript on the advantages of the supramolecular method compared to covalent fluorogenic labelling methods. The advantages of the supramolecular method are not sufficiently pin-pointed yet. For example, it would be beneficial to mention the rapid response times (Fig. 2b), which is highly beneficial, for example, for pulse-chase assays. Perhaps, it would be even possible to roughly assess the kinetics of the supramolecular exchange and compare it with well-established covalent methods. Also the potential advantages to target small, minimalistic labels with the supramolecular strategy should be compared to existing methods using streptavidin-biotin or covalent fluorogenic probes.

Conceivable issues include the limited cell permeability of streptavidin for intracellular imaging or the potential instability of common covalent labels compared to an adamantane label.

References that may be useful to the authors to address this request are listed below:
Nat. Rev. Chem. 2019, 3, 393–400. https://doi.org/10.1038/s41570-019-0095-1
Angew. Chem. Int. Ed. 2023, 62, e202314373. https://doi.org/10.1002/anie.202314373
Nat. Commun. 2024, 15, 1851. https://doi.org/10.1038/s41467-024-46034-z
ACS Med. Chem. Lett. 2011, 2 (12), 885–889. https://doi.org/10.1021/ml200187j
Chem. Commun. 2024, 60, 4810-4813. https://doi.org/10.1039/D4CC00602J

Ans. In response to the reviewer's suggestion, we have now added a more detailed discussion on the advantages of the supramolecular method compared to covalent or protein-based methods. The suggested references were particularly useful for this discussion and have been cited in the text. The following text has been included:
''The use of synthetic host-guest supramolecular labels for fluorogenic imaging offers several advantages over covalent click labels and well-known protein-based binding pairs, such as streptavidin-biotin.[77-80] For instance, host-guest complexation kinetics are typically diffusion-controlled and occur at a much faster rate (k_{on}~10^9–10^{10} M^{-1} s^{-1}) compared to their covalent counterparts, which are kinetically slower (typically k_{on}~ 1–10^4 M^{-1} s^{-1}). This rapid labeling is particularly beneficial for precise pulse-chase assays, where a high labeling rate is crucial. Unlike covalent reactive groups, which often compromise stability for reactivity, synthetic host-guest labels (e.g., CB7 and 1-adamantylamine) utilize a stable chemical structure that is robust in physiological environments. Additionally, these small synthetic host-guest pairs avoid common issues associated with biotin-streptavidin pairs, such as the large size and potential immunogenicity of proteins, as well as interference from endogenous biotin. The smaller size of synthetic host-guest recognition pairs, compared to protein-based binding pairs, also facilitates efficient cellular uptake. Given these advantages, we believe that these host-guest fluorogenic probes will be a valuable tool for biological imaging and will create new opportunities for microscopic and nanoscopic investigations in vitro, in vivo, and in diagnostic settings.”

Comment 2: A further minor issue is found on p.10, left column, lines 3-5, where authors state that "excellent quenching of CB7-TAMRA fluorescence upon addition of 0.2 equivalent of XYL-AuNP", which does not match the data in the inset of Fig. 6c.

Ans. The 0.2 equivalent was mentioned because the titration was performed up to that amount. However, we agree with the reviewer that quenching was nearly saturated with 0.02 equivalent of XYL-NP. We have, therefore, revised the text and the number accordingly.
